# Variability, Drivers, and Utility of Genetic Diversity‐Area Relationships in Terrestrial Vertebrates

**DOI:** 10.1111/ele.70306

**Published:** 2025-12-31

**Authors:** Chloé Schmidt, Sean Hoban, Deborah M. Leigh, Walter Jetz, Colin J. Garroway

**Affiliations:** ^1^ Department of Biology Dalhousie University Halifax Nova Scotia Canada; ^2^ German Centre for Integrative Biodiversity Research (iDiv) Halle‐Jena‐Leipzig Leipzig Germany; ^3^ Department of Ecology and Evolutionary Biology Yale University New Haven Connecticut USA; ^4^ Center for Biodiversity and Global Change Yale University New Haven Connecticut USA; ^5^ The Center for Tree Science The Morton Arboretum Lisle Illinois USA; ^6^ Swiss Federal Research Institute for Forest, Snow, and Landscape Research WSL Birmensdorf Switzerland; ^7^ LOEWE Centre for Translational Biodiversity Genomics Frankfurt Germany; ^8^ Senckenberg Research Institute Frankfurt Germany; ^9^ Institute of Ecology, Evolution, and Diversity, Faculty of Biosciences Goethe University Frankfurt Frankfurt Germany; ^10^ Department of Biological Sciences University of Manitoba Winnipeg Manitoba Canada

**Keywords:** biodiversity, conservation, *F*_ST_, genetic indicators, macrogenetics, population differentiation, species‐area relationship, vertebrates, zMAR

## Abstract

Maintaining genetic diversity within and among populations is critical for conservation and a prominent goal of the Kunming‐Montreal Global Biodiversity Framework. However, direct estimates of genetic diversity are unavailable for most species, and time and resources are insufficient to fill these substantial data gaps and meet conservation target timelines. We evaluated a proxy‐based prediction of genetic diversity loss, the Genetic Diversity Area Relationship (GDAR), which describes relationships between genetic diversity and the geographic area occupied by a species. We estimated differences in three metrics of genetic diversity relative to sample area using 55 previously published datasets from 51 species. GDARs were highly variable across species and strongly dependent on population structure, with no clear differences across vertebrate classes. Traits correlated with population structure and study area explained 35%–45% of the variation in GDARs. Across genetic diversity metrics, prediction accuracy was highest for GDARs estimated from allele count compared to allelic richness and gene diversity. Our findings suggest there are opportunities for refining taxon‐specific GDARs to predict genetic diversity loss following area loss in the absence of genetic data.

## Graphical abstract

The Genetic Diversity Area Relationship (GDAR) is a proxy‐based prediction of genetic diversity loss. We empirically derived and assessed variation in GDARs across 51 terrestrial vertebrate species, and explored the extent to which GDAR slopes were related to traits that affect population structure. Our findings suggest there are opportunities for refining taxon‐specific GDARs using species attributes to predict genetic diversity loss following area loss in the absence of genetic data.

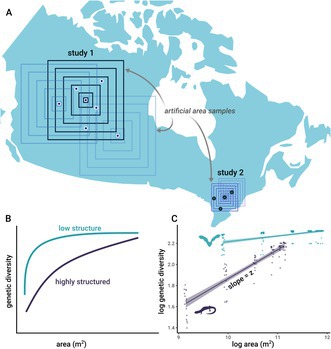

## 1 Introduction

Genetic diversity is a fundamental component of biodiversity that is critical for the long‐term resilience of populations and species (DeWoody et al. 2021; Lande and Shannon 1996). Parties to the Convention on Biological Diversity formally committed to conserving genetic diversity and to measuring the progress toward this goal in 2022 with the signing of the Kunming‐Montreal Global Biodiversity Framework (‘KM GBF’ Goal A, Target 4; CBD 2022; Hoban et al. 2023). However, monitoring changes in genetic diversity is difficult and has been neglected due to cost, expertise barriers in data production, and difficulties in sample collection. Consequently, we lack sufficient genetic data for the assessment and monitoring of most species (Paz‐Vinas et al. 2025), and the substantial resources needed for direct genetic assessments of species at the global scale are not on the horizon (Hogg 2024). With the necessarily ambitious KM GBF target of halting the loss of genetic diversity and restoring conditions to support adaptive capacity by 2030, approximations or indicators of genetic diversity are urgently needed to support genetic diversity monitoring and conservation.

### 1.1 Diversity‐Area Relationships From Species to Alleles

Genetic diversity change could potentially be approximated by extending the concept of species‐area relationships (SAR) to genetic diversity (Exposito‐Alonso et al. 2022; Mimura et al. 2017). The SAR is a simple, widely used relationship in conservation that describes how species richness decreases from larger toward progressively smaller areas (Connor and McCoy 1979; Lomolino 2000; Storch et al. 2012; He and Hubbell 2011). For conservation purposes, the SAR has been used to approximate changes in species richness across regions differing in amounts of habitat over space and time. Applying the SAR at a genetic level could similarly serve as a shorthand for gauging genetic diversity loss as habitat and range sizes shrink (Exposito‐Alonso et al. 2022; Mimura et al. 2017).

**FIGURE 1:**
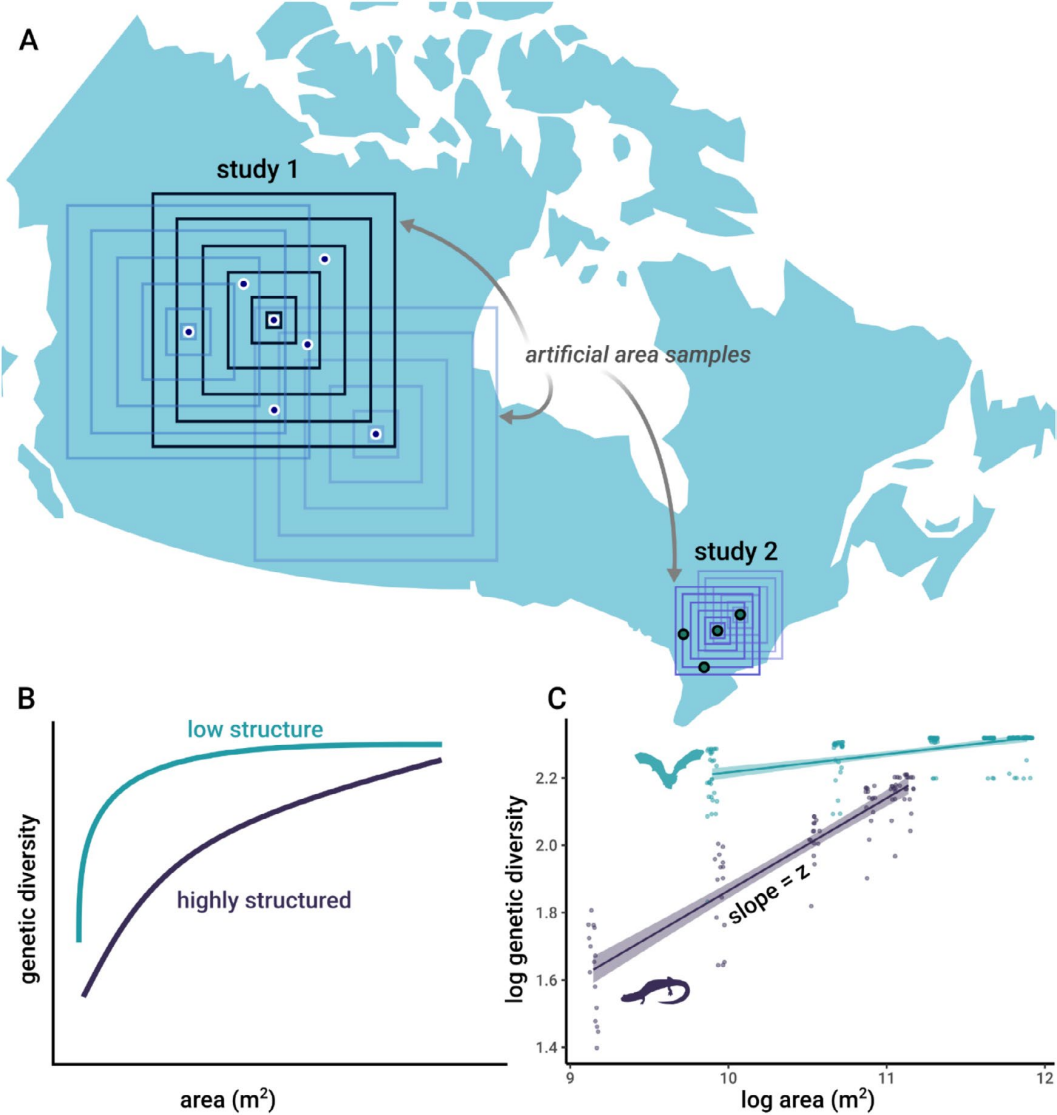
GDAR sampling design and predictions for two hypothetical studies. (A) For each sample location within a study, we assessed genetic diversity as aggregates of all samples contained within each of 6 concentric quadrats of increasing size. (B) We then constructed a study's GDAR as the average relationship with area across all its samples assessed in this way, with an estimated scaling exponent, *z*, describing the relationship between area and genetic diversity in log–log space. In species with little genetic structuring (low *F*_ST_), alleles are more evenly spread over the landscape and even small areas can harbour large proportions of the alleles present in the entire study sample. Thus, GDARs will more rapidly level off (shallow slope, smaller *z*) compared to species that are highly genetically structured (high *F*_ST_, steep slope, high *z*). (C) Example of two species with contrasting movement abilities, the little brown bat ( *Myotis lucifugus* ) and the Mount Lyell salamander ( *Hydromantes platycephalus* ). In this example, genetic diversity is measured using allele count. Shaded regions are 95% confidence intervals of the fitted line. Due to its flight ability, we predict that the little brown bat should exhibit less population structure than a comparatively less vagile salamander species due to higher possibility of gene flow.

The traditional simple power law formulation of the SAR is: log (S) = log(c) + z* log (A), where *S* is the number of species in a region with area *A*, *c* is a constant, and the exponent *z* characterises the linear rate of increase in species number with area. Different approaches for characterising SARs exist (Keil et al. 2015); a traditional one is that of a nested design (Figure 1), where consecutively larger surrounding areas are assessed to estimate the rate at which the number of species sampled increases.

In SARs, *z* describes the spatial turnover of species or portion of species shared between a larger and a nested smaller area—that is, beta diversity (Báldi 2008; Triantis et al. 2003). For specific sampling design cases and geographic range distributions, the connection is mathematically defined (Crist and Veech 2006; Tjørve and Tjørve 2008), and accounting for these beta diversity estimates fully determines *z* values and vice versa. In population genetics, beta diversity is typically quantified using the fixation index, *F*_ST_, a standard measure of allele frequency variation attributable to population genetic structure, which has very well‐understood theoretical underpinnings (Weir and Hill 2002; Wright 1943, 1951). *F*_ST_ specifically captures the change in heterozygosity due to population subdivision. As with the SAR, the exponent *z* in the GDAR describes spatial turnover of alleles and is thus a measure of beta diversity at the genetic level. Because of this, it is expected that *z* should be closely related to *F*_ST_.

Population genetic structure is generally correlated with ecological attributes of species (Gamba and Muchhala 2020; Hillman et al. 2014; Medina et al. 2018) and landscapes (Handel 1983; Schmidt et al. 2022) that affect gene flow and population size. Given our strong theoretical understanding of *F*_ST_, assessing its empirical connection to GDAR *z* values has the potential to link newly emerging perspectives related to GDARs with population genetics to advance conservation. By repurposing publicly available microsatellite genetic datasets from wild populations, we expand the number of terrestrial vertebrates with empirically derived GDARs to 51 species and test putative drivers of *z* and *F*_ST_ to explore whether ecological predictors would support a straightforward use of GDARs by conservation practitioners. We focus on three metrics of genetic diversity (gene diversity, allelic richness, and allele count) that reflect adaptive capacity on different timescales to build on previous work (Allendorf et al. 2024; Exposito‐Alonso et al. 2022). Because *F*_ST_ estimation is a heterozygosity‐based metric, we predict *z* values derived from gene diversity will be most strongly associated with *F*_ST_. If GDAR *z* values were either reasonably similar among species or if their variation were well captured and predictable, an opportunity would exist for employing the GDAR relationship to predict genetic diversity change for individual threatened species, rather than only at a global or regional level, helping conservation prioritisation.

## 2 Results

### 2.1 Variation in Genetic Diversity Area Relationships

**FIGURE 2:**
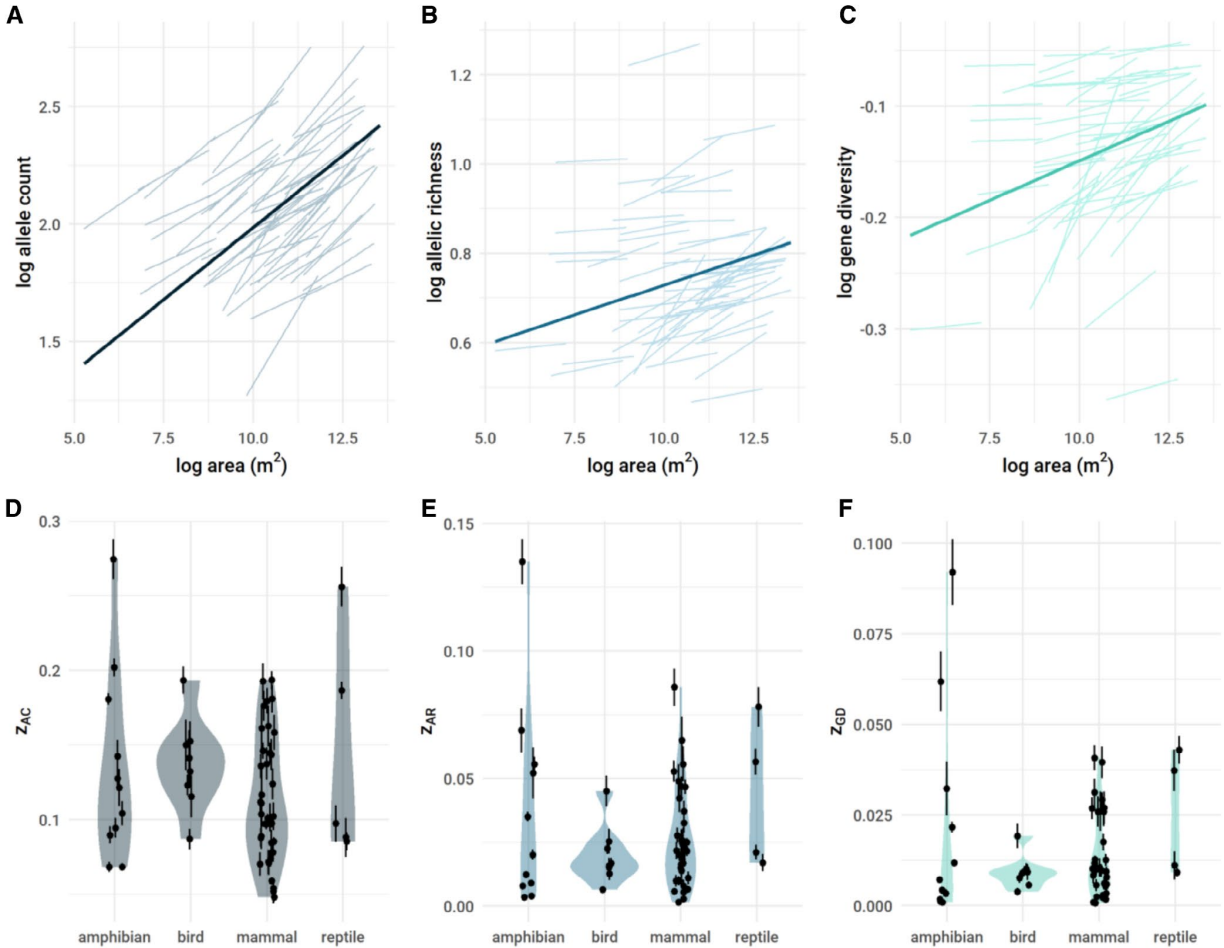
(A–C) Genetic diversity—area relationships in terrestrial vertebrates assessed across three genetic metrics. Lighter lines are log diversity vs. log area for each dataset. Dark lines depict the overall relationship across all datasets. The slope of each line is the scaling exponent *z*. (D–F) Plots of *z* values for each genetic metric (*z*_AC_ = allele count, *z*_AR_ = allelic richness, *z*_GD_ = gene diversity) by vertebrate class. Points represent estimated *z* values and lines denote the standard error.

The GDAR can be estimated identically to the SAR by treating alleles as species. We artificially sampled each genetic dataset using a nested GDAR design (sampling concentric areas of increasing size) and estimated *z* values as the slope of the log–log relationship between the number of alleles sampled and the size of the area from which they were sampled (Figure 1; see Methods). There was considerable variation in the shape and strength of GDARs across the 51 terrestrial vertebrate species we assessed, with no clear differences among taxonomic classes (Figure 2, Tables S1 and S2). For gene diversity, *z*_GD_ in amphibians, mammals, and reptiles was significantly larger than zero, but values for birds were indistinguishable from zero (Table S3). This pattern was weaker for allelic richness, and for allele count, *z* values for all taxa were significantly larger than zero (Table S3). Across metrics, nearly all datasets showed significant area dependence. In general, allelic richness and gene diversity had shallower and statistically weaker relationships with area than those of allele count (Figure 2, Table S2, Figure S2), likely because these metrics are less sensitive to rare alleles.

### 2.2 Ecological Predictors of Population Structure and GDAR

**FIGURE 3:**
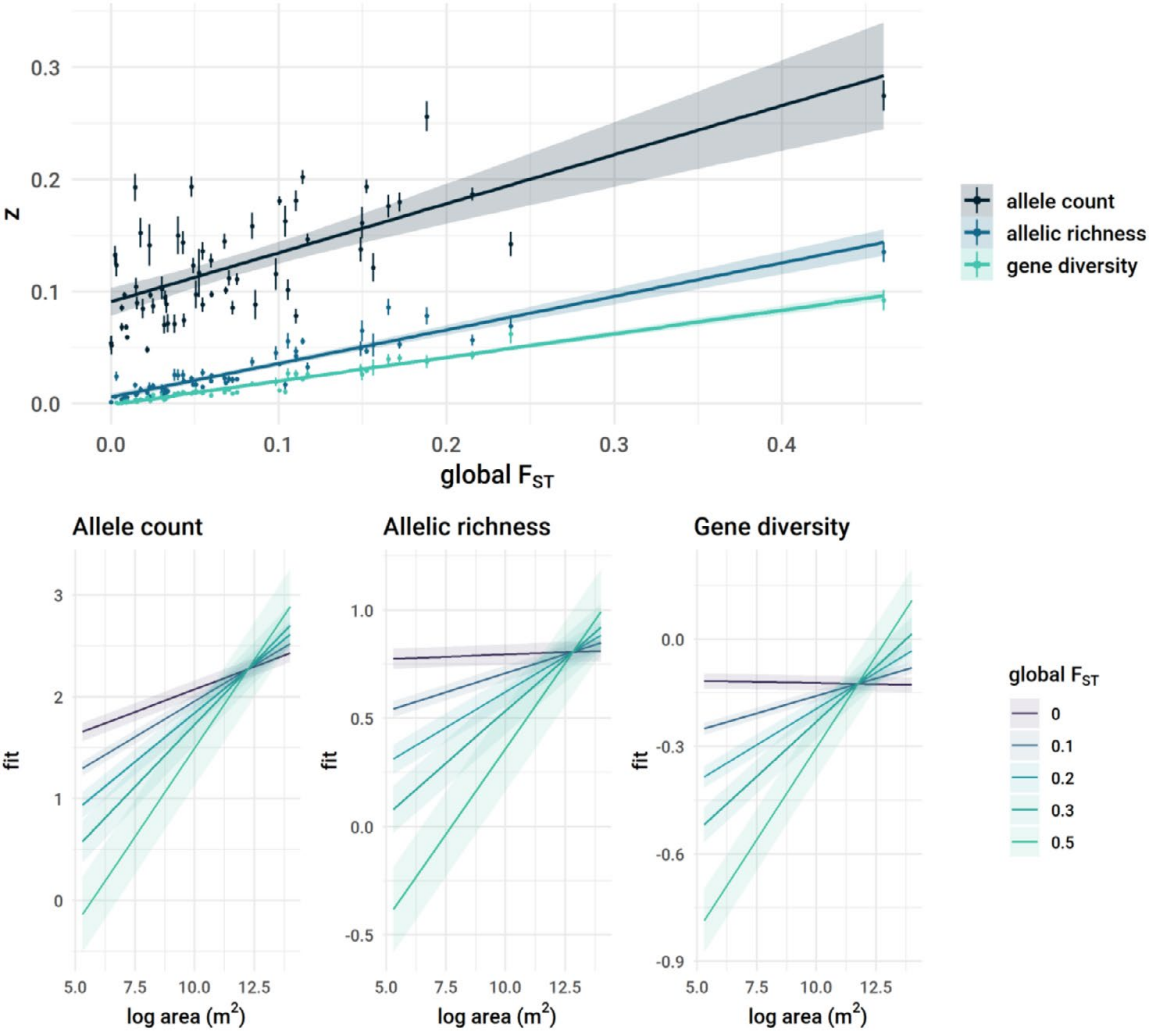
The effect of area on genetic diversity depends on population differentiation (measured with global *F*_ST_). (Top) The top plot shows the strong correlation between study‐specific *z* values and *F*_ST_ for all metrics of genetic diversity. Each point represents a single genetic dataset (some species are represented by multiple datasets), and lines denote the standard error of *z* estimates. (Bottom) Models fitted to the 51 species that included the main effects and interaction of log area and F_ST_ as predictors of genetic diversity. Ribbons denote 95% confidence intervals around the mean effect (solid line). For all diversity metrics, the effect of area strengthens as *F*_ST_ increases. Gene diversity and allelic richness are unrelated to area when there is no population structure (*F*_ST_ = 0). The three metrics are shown in order of decreasing sensitivity to sample size: allele counts, rarefied allelic richness, and gene diversity.

We estimated global *F*_ST_ for each genetic dataset to quantify population genetic structure, and confirmed that population structure moderated relationships between genetic diversity and area (Figure 3). Using a hierarchical regression model combining data across all species, we found species with more population structure (higher *F*_ST_) lost alleles more rapidly when sampling increasingly smaller geographic areas (Figure 3, Table S4). As predicted, *z* for each metric was consistently strongly correlated with *F*_ST_, however, this correlation was strongest for *z* estimated from gene diversity and allelic richness (Pearson r_allele count_ = 0.69 [0.53–0.80, 95% CI], r_allelic richness_ = 0.94 [0.90–0.96]; r_gene diversity_ = 0.98 [0.96–0.99]; Figure 3). These different strengths of relationships with F_ST_ are in line with our predictions because *F*_ST_ estimation is based on varying frequencies of alleles rather than counts.

**FIGURE 4:**
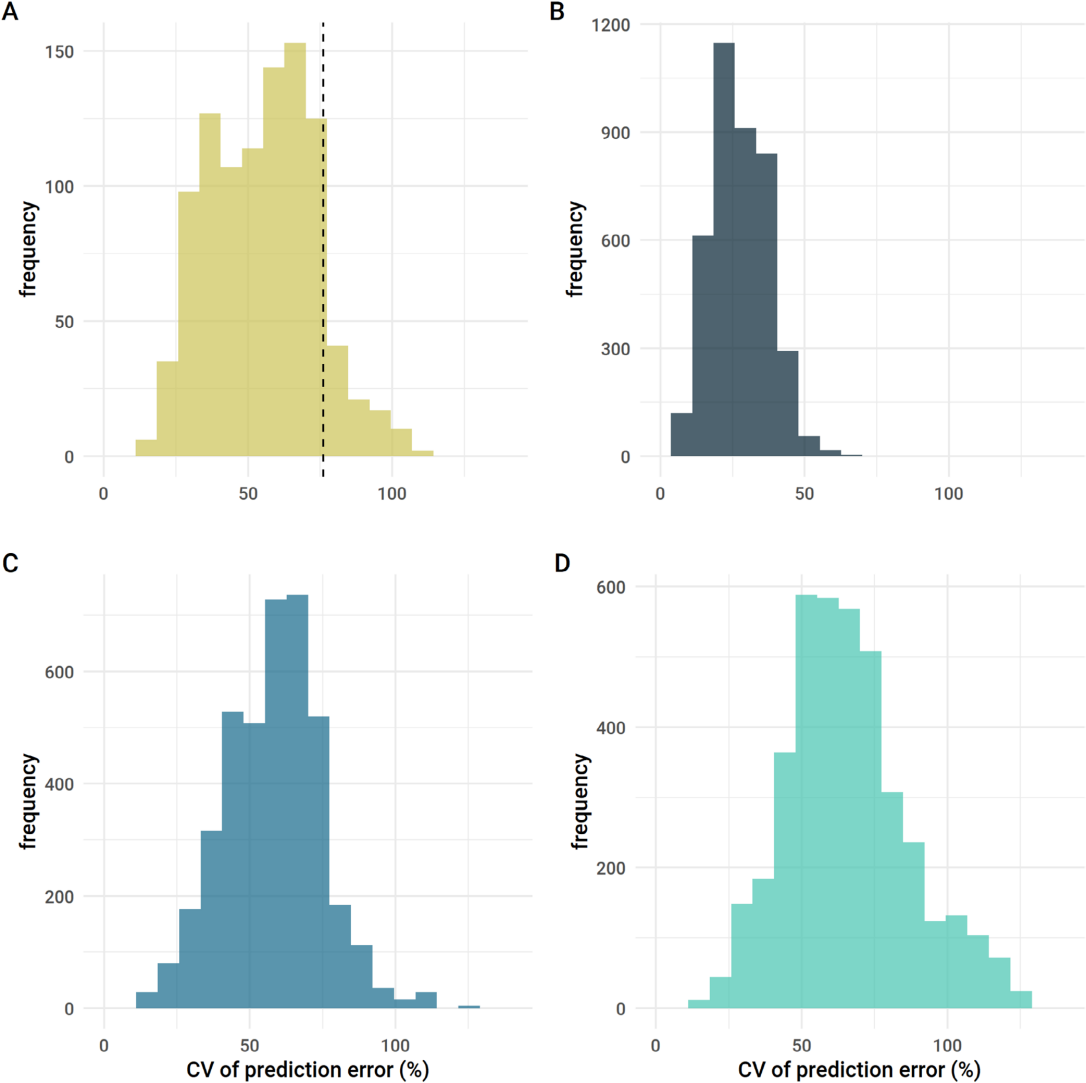
Trait‐based prediction error for *F*_ST_ and *z*. Plots show the coefficient of variation (CV) of the root mean square error (RMSE) summarised across 1000 partitions of test datasets for *F*_ST_ (A), *z* for allele count (B), *z* for allelic richness (C), and *z* for gene diversity (D). The dashed vertical line shows the CV of the RMSE for predictions of *F*_ST_ in an independent dataset, the MacroPopGen dataset, where the average prediction error was approximately 76% of observed species *F*_ST_ values. Prediction error was lowest for *z* based on allele count, where error was on average 29% of observed *z* values.

We next tested the extent to which we could predict *F*_ST_ and *z* from readily available trait data for 33 mammal species. Due to the stronger theoretical and empirical correlation between F_ST_ and z_GD_, we hypothesized that z_GD_ would be better predicted than z_AR_ or z_AC_. We used three traits related to the spatial distribution of individuals and dispersal ability that are generally related to population structure (Bohonak 1999; Loveless and Hamrick 1984): body size, home range size, and geographic range size. We included the total sampling area of each study as an additional predictor. Together, these variables explained approximately half of the variation in species' global *F*_ST_ (58%) and less than half of the variation in *z* values (36%–45% across genetic metrics; Table S5). Partitioning the data into training and test sets, the average relative prediction error was nearly 60% of observed *F*_ST_ values (mean coefficient of variation [CV] = 0.57), and similar for *z*_GD_ (CV = 0.64) and *z*_AR_ (CV = 0.55; Figure 4). Error was lower for allele count‐derived *z*_AC_ predictions, indicating better predictive accuracy (CV = 0.29; Figure 4). Finally, we tested the capacity of this model to predict *F*_ST_ in 37 species from an independently collected literature‐based dataset containing microsatellite *F*_ST_ values, MacroPopGen (Lawrence et al. 2018, 2019), and found low predictive capacity with an average prediction error of 76% of observed values (CV = 0.76; Figure 4).

## 3 Discussion

Our results demonstrate that variation in GDARs across datasets, taxa, and genetic diversity metrics is associated with, but not easily predicted by, species traits. Although variation in population structure (particularly measured by *F*_ST_) was relatively well‐explained by morphological and ecological traits related to dispersal and gene flow, predictions of *F*_ST_ do not appear to generalise well when extended to new data beyond the training set (Figure 4, Table S5). In general, *z* values tended to be less strongly associated with species traits than F_ST_. Our findings from 51 species extend previous work that suggested *z* may not be related to species attributes, including Red List status, kingdom, mode of reproduction, pollination strategy, mobility, or range area in 20 species (Exposito‐Alonso et al. 2022). This suggests the potential to enhance our capacity to predict the value of *z* using larger numbers of species as more data become available.

We detected general patterns that may be informative for future work predicting *z*. Allele‐count based *z* values were more strongly related to area than z_GD_ or z_AR_. This is likely because allele count is highly sample size dependent, and sampling greater areas necessarily means sampling more individuals and thus more rare alleles. Allelic richness (number of alleles standardised using rarefaction; El Mousadik and Petit 1996) and gene diversity (the average probability that two randomly sampled alleles are different in a nonrandom mating population; Nei 1973) are less sensitive to sample size differences and rare alleles (Charlesworth and Charlesworth 2010). Predictive error was also lowest for allele count. This suggests that *z* values to predict changes in raw numbers of alleles can be more readily honed using estimates of abundance or population density if available. In general, there were no clear differences in *z* values across taxonomic groups (Figure 2, Table S2). However, z_GD_ was indistinguishable from zero for birds, and tended to be greater than zero for amphibians, mammals, and reptiles (Table S3). This pattern also held for *F*_ST_ and, more weakly, for allelic richness. This supports the expectation that flying species tend to have less genetically structured populations than walking species. These patterns did not hold for *z* values based on allele count. Home range size and study area were significantly related to z_AC_ in our models, and z_AR_ and z_GD_ were also associated with body size. Additionally, z_AR_ was correlated with geographic range size. Because *z* values for allelic richness and gene diversity were associated with more traits and were more strongly correlated with *F*_ST_, these metrics have the highest potential for increased predictability from trait‐based models with larger data sets.

Decisions about predicting changes in numbers of alleles (allele count, allelic richness) or allele frequencies (gene diversity) will largely depend on the conservation goal. Numbers of alleles respond more quickly to population size decline than allele frequencies, which can remain stable for generations before starting to decline (Allendorf et al. 2024; El Mousadik and Petit 1996; Nei et al. 1975). High gene diversity is beneficial for short‐term adaptive responses to environmental change and avoidance of inbreeding, while higher numbers of alleles determine the adaptive capacity of populations over longer timescales (Allendorf et al. 2024). Predicting losses of alleles or reduced gene diversity with GDARs can be an informative first step for broad surveys of genetic diversity loss, particularly in the short term; however, GDARs likely underestimate long‐term consequences of continued population decline. Range contractions can cause immediate loss of alleles due to the loss of individuals, and habitat fragmentation due to patchy area loss can decrease gene flow across species ranges. Smaller population sizes and reduced gene flow will, in turn, increase the strength of genetic drift. This would accelerate the loss of genetic diversity for generations to come and introduce additional population structure, causing *z* to increase over time (Gargiulo et al. 2024; Mualim et al. 2024; Pflüger et al. 2019; Pinto et al. 2024). GDAR *z* values can be reassessed over time depending on the amount and configuration of area loss to provide more realistic predictions over the short and long term.

The uncertainty in relationships between *z* values, area, and traits we find is likely caused in part by sample distribution. Most species sampled in our dataset and the independent dataset we tested, MacroPopGen, had irregular sampling schemes, different numbers of individuals sampled across sample locations, and were not range‐wide as in previous work (Exposito‐Alonso et al. 2022). Irregular sample placement and sample size variation can introduce variation in relationships between genetic diversity and area. In species with highly structured populations, there are often hot and coldspots of genetic diversity throughout a range, and the area occupied by hot and coldspots will vary (Schmidt et al. 2023). This spatial variation in the distribution of genetic diversity can be seen in the smallest area samples of GDARs and in the variability in the degree of fit of GDAR relationships, where in some species small areas contain high genetic diversity (Figure S2). Simulated area samples may have low diversity due to factors unrelated to area size. When making trait‐based predictions of *z*, haphazardly sampling regional structure may yield more idiosyncratic results than whole‐species population structure, adding statistical noise. It is also possible that the relatively fewer loci typically used with microsatellites compared to genomic datasets may generate uncertainty in GDARs; however, this effect was not apparent in our data (Figure S5). Indeed, the spread of data around GDAR lines of best fit (Figure S2) is similar to that seen with single nucleotide polymorphism data (Exposito‐Alonso et al. 2022), suggesting that species‐specific spatial population structure and sampling design may be the primary sources of uncertainty in GDARs. High‐quality range‐wide training data may enhance the predictability of *z* values; however, these are not currently available for most species (Paz‐Vinas et al. 2025). Broader explorations of the relationship between global F_ST_ and *z* across species and ecological and evolutionary contexts are needed to further assess the practical predictability of *z* in GDARs.

Our work identifies other considerations for standardisation and data collection to support progress toward a more effective use of genetic data for conservation. A standout issue is the very limited availability of genetic data. We used microsatellite data, which is an abundant data type suitable for estimating GDARs that remains widely used for wildlife studies—particularly for species lacking reference genomes. However, *z* values obtained from different types of genetic data, or based on F_ST_ directly, are likely not comparable. All of the *z* values we estimated were below 0.3, the average *z* value across 20 species reported by Exposito‐Alonso et al., derived from allele counts using genomic data (Exposito‐Alonso et al. 2022); and below 0.25, the intermediate *z* value proposed by Mimura et al. (2017) after exploring GDARs in 27 plant species using a previously published amplified fragment length polymorphism (AFLP) dataset (Alsos et al. 2012). This complicates choosing *z* values for species or areas where genetic structure is unknown. A better understanding of the factors that influence the distribution of genetic *z* values across data types, taxa, and sampling strategies, and how these interact with F_ST_, can help refine predictions of genetic diversity loss based on genetic data that are already available.

## 4 Conclusions

Our understanding of GDARs is in its infancy, and further development may improve our ability to use general rules to predict genetic diversity loss. Uncertainty in predicting *z* values from species traits means that additional ecological, evolutionary and environmental information is likely necessary to estimate *z* values for a given area or subset of species. We encourage more work to achieve a greater understanding of the factors that shape GDARs across species before they are incorporated into the conservation genetic toolbox. Access to genetic data and robust proxies of genetic diversity and its loss remain essential for effectively protecting genetic diversity.

## 5 Methods

### 5.1 Genetic Diversity Area Relationships

We used 55 publicly available microsatellite datasets that were previously compiled by Schmidt et al. (for detailed methods on their compilation, see Schmidt et al. 2020, 2023; Schmidt and Garroway 2022). Briefly, we queried DataONE (a platform to access multiple data repositories) and the Dryad Digital Repository between 2017 and 2021 using binomial species names and ‘microsat*’ as keywords. We retained data from wild populations in their native range and set a minimum sample size of 5 individuals per sample location identified in the original publications. The datasets comprised 51 species with a median of 579 individuals per dataset, ranging from 127 to 2232 individuals (Table S1). We set an a priori cutoff of at least 10 sample locations per dataset for this study (median: 22 locations; range: 12–100). We did not set thresholds for the minimum area sampled because we were primarily interested in estimating *z* and *F*_ST_ from each dataset and testing relationships between them, which does not require sample sites to be representative of the species as a whole. We quantified population structure across the entire species sample using global *F*_ST_. We estimated global *F*_ST_ for each dataset in R (R Core Team 2021) with the basic stats() function in the hierfstat package (Goudet and Jombart 2015), which estimates *F*_ST_ as (H_t_—H_s_)/H_t_, where H_t_ = total heterozygosity and H_s_ = mean heterozygosity of subpopulations.

To construct a GDAR for each dataset, we took a nested approach to artificially sample each study area. We sampled a square area centered on each genetic sample location with half‐side lengths that were 10%, 25%, 50%, 75%, 90% and 99% of the study extent.

For each area sample, we recorded (1) the total number of alleles summed across loci; (2) rarefied allelic richness (hereafter *allelic richness*; rarefied to the smallest sample size of a sample location for a given dataset) (El Mousadik and Petit 1996); and (3) gene diversity (evenness; Nei 1973). These metrics differ in their sensitivity to the presence of rare alleles and thus to the number of individuals sampled. Allele count weights all alleles equally and is most strongly affected by sample size. It is also the most representative of the extinction of a ‘mutation’ or allele, which has been argued to be a key focus of genetic conservation (Allendorf et al. 2024). Gene diversity is most affected by the frequencies of common alleles, while rarefied allelic richness is intermediately affected by rare alleles. Allele count is analogous to the number of single nucleotide polymorphisms, or allelic variants, used in (Exposito‐Alonso et al. 2022). We estimated *z* of the power‐law GDAR (genetic diversity = cA^z^) for each genetic metric by taking the slope estimated for the relationship between log area and log genetic diversity. For datasets where this slope was not significant (linear regression, *p* < 0.05), we recorded slope (*z*) values as NA. This resulted in the removal of 4 datasets for allelic richness (zAR) and 6 datasets for gene diversity (zGD) for downstream analyses.

To quantify the dependence between the effects of area and population structure on genetic diversity, we ran a multiple regression with log area, global *F*_ST_, and their interaction as predictors of each diversity metric (allele counts, allelic richness, and gene diversity) for a total of 3 models. We included all datasets in a single model with study as a random intercept. Models were fit in the lme4 package (Bates et al. 2015) in R. We then compared the effect sizes of area to models with only area as a predictor.

### 5.2 Trait‐Based Predictions of *z*

We then explored the predictability of global *F*_ST_ and *z* values from readily accessible species‐level trait data. We tested 3 traits that are related to population structure: body mass (g), individual home range area (km^2^), and geographic range size (km^2^). We restricted our analysis to mammals because they had the most data, and we could make consistent predictions about the relationships between traits and population structure. Generally, larger species tend to disperse further, have larger individual home ranges and larger geographic ranges (Bowman et al. 2002; Jetz et al. 2004). Additionally, species with larger home ranges generally have less spatially structured populations, while structure due to isolation by distance is likely higher in species with larger distributional ranges.

We compiled adult body mass data from the PanTHERIA database (Jones et al. 2009) via the R package traitdata (RS‐eco 2022). We used home range sizes available from the HomeRange dataset (Broekman et al. 2023; Hoeks 2023), using individual, adult home ranges estimated from wild animals. Finally, we obtained data on geographic range sizes from species distribution maps available from the IUCN (IUCN 2021). We filled in missing data values from the literature where possible. For species with missing home range data, we used the mean value of the genus if available. Because our focus was on model predictive capacity, we disregarded collinearity among predictor variables. We also included the sample extent of each dataset (i.e., the area of the bounding box covering all sample sites) as an additional predictor. All three traits were available for 33 of the species in our data set. We related these predictors to global *F*_ST_ and *z* values using multiple linear regressions. For models with global *F*_ST_ as a response variable, which varies between 0 and 1, we fit beta regressions with a logit link function in the betareg package (Cribari‐Neto and Zeileis 2010). We used normally distributed errors for models with *z* values as response variables because *z* is not by definition bound by 0 and 1.

We then tested model predictive accuracy by partitioning our dataset into training and test datasets (80% and 20% of the data, respectively). We created 1000 partitions and recorded the coefficient of variation (root mean squared error divided by the mean observed *z* or *F*_ST_) to measure the relative error rates of each model.

As an additional test, we assessed whether our model maintained similar predictive capacity for mammal *F*_ST_ values derived from an independent dataset, the MacroPopGen database (Lawrence et al. 2018, 2019). MacroPopGen reports pairwise or global *F*_ST_ values for terrestrial vertebrates and freshwater fish across the Americas compiled from literature reports and is based on studies that used microsatellite data. After filtering for mammal species, we applied additional filters for sample locations with more than 5 individuals and species with 10 or more sample locations for consistency with our compiled dataset. We note *F*_ST_ estimates from data with fewer individuals sampled or more local sampling are less likely to be associated with species‐level traits. When global *F*_ST_ was not reported, we calculated mean pairwise *F*_ST_ values for populations within a study, which are correlated with global *F*_ST_. In total, 53 *F*_ST_ estimates from MacroPopGen and trait data were available for 36 species (Table S6). We assessed predictability using the same approach described above.

## Author Contributions

All authors developed the project idea. Chloé Schmidt curated data and performed the analyses with input from Colin J. Garroway and Walter Jetz. Chloé Schmidt wrote the first draft of the manuscript and all authors contributed substantially to revisions.

## Funding

This work was supported by Deutsche Forschungsgemeinschaft. Natural Sciences and Engineering Research Council of Canada.

## Supporting information


**Table S1:** Data summary. The number of sites, total individuals across all sites, estimated global F_ST_, estimated *z* values for each dataset (rows).
**Table S2:**. GDAR scaling exponents (*z*‐values) for terrestrial vertebrates summarised across taxonomic groups (overall values) and for each taxonomic class.
**Table S3:**. Model summaries testing whether *F*_ST_ or *z* values differ from zero across taxa.
**Table S4:** Comparison of area effect sizes between models with area alone, and global *F*_ST_ and an area × *F*_ST_ interaction as predictors.
**Table S5:** Model summaries for relationships between *F*_ST_, *z* values derived from allele count (zAC), allelic richness (zAR), and gene diversity (zGD), and predictor variables including: home range size (km^2^), species range size (km^2^), species body mass (g), and the area of the spatial extent of the sample locations in each dataset (km^2^).
**Table S6:** *F*_ST_ estimates from the MacroPopGen database and associated trait data.
**Figure S1:** Box plots of variance in genetic diversity explained by area (*R* ^2^) for each genetic metric.
**Figure S2:** Genetic diversity versus area plotted on a log–log scale for all 61 datasets.
**Figure S3:** Comparison of *F*_ST_ values from the data analysed here versus mean *F_ST_* estimates per species within the mammal MacroPopGen dataset for 12 species in common across both datasets.
**Figure S4:** Observed MacroPopGen *F*_ST_ versus *F_ST_* values predicted based on a model derived from the data analyzed here.
**Figure S5:** GDAR fit (*R* ^2^ of log–log relationships between area and each genetic metric) versus the number of loci and number of alleles per dataset.

## Data Availability

All data underlying this work are publicly available (see Table S1). Scripts for querying the Dryad Data Repository are available here: https://github.com/chloewsch/dryadkeyword. Data and code used in analyses are available on Figshare (DOI: 10.6084/m9.figshare.28883060) and GitHub: https://github.com/chloewsch/GDAR.
