# Supplementary material for: Variability, Drivers, and Utility of Genetic Diversity‐Area Relationships in Terrestrial Vertebrates
Source: Ecol Lett. 2025 Dec 31;29(1):e70306. doi: 10.1111/ele.70306 (PMC12755187; doi:10.1111/ele.70306)

Supplementary information for: Variability, drivers, and utility of genetic diversity-area relationships in terrestrial vertebrates

Chloé Schmidt, Sean Hoban, Deborah M. Leigh, Walter Jetz, Colin J. Garroway

Contents:

Tables S1 – S6

Figures S1 – S5

Table S1. Data summary. The number of sites, total individuals across all sites, estimated global F_{ST}, estimated z values for each genetic metric, and references are given for each dataset (rows). NA values for *z* indicate that the effect of area on genetic diversity was not significant and corresponding datasets were thus omitted from further analyses of *z*.

class	species	sites	individuals	global F_{ST}	zAC	zAR	zGD	references
amphibian	*Ambystoma maculatum*	56	1292	0.0092	0.07	0.00	0.00	[1,2]
amphibian	*Lithobates sylvaticus*	39	888	0.0064	0.07	0.00	0.00	[1,2]
amphibian	*Lithobates sylvaticus*	29	766	0.0598	0.13	0.02	0.01	[3,4]
amphibian	*Ambystoma maculatum*	19	489	0.1564	0.12	0.05	0.03	[5,6]
amphibian	*Ascaphus montanus*	100	1968	0.1143	0.20	0.06	0.02	[7,8]
amphibian	*Ambystoma barbouri*	76	1601	0.1005	0.18	0.03	0.01	[9,10]
amphibian	*Plethodon albagula*	21	343	0.0149	0.10	0.01	0.00	[11,12]
amphibian	*Ambystoma maculatum*	22	626	0.0324	0.09	0.01	0.00	[13,14]
amphibian	*Lithobates sylvaticus*	22	469	0.0156	0.09	0.01	0.00	[13,14]
amphibian	*Rana draytonii*	17	298	0.2384	0.14	0.07	0.06	[15,16]
amphibian	*Hydromantes platycephalus*	15	195	0.4605	0.27	0.14	0.09	[17,18]
bird	*Poecile atricapillus*	32	911	0.0480	0.19	0.02	0.01	[19,20]
bird	*Campylorhynchus brunneicapillus*	12	363	0.0984	0.12	0.05	0.02	[21,22]
bird	*Geospiza fortis*	11	202	0.0401	0.15	0.03	0.01	[23,24]
bird	*Geospiza fuliginosa*	11	198	0.0229	0.14	0.02	0.01	[23,24]
bird	*Geospiza fuliginosa*	21	517	0.0025	0.13	0.01	NA	[25,26]
bird	*Aphelocoma californica*	30	463	0.0492	0.12	0.02	0.01	[27,28]
bird	*Poecile hudsonicus*	13	260	0.0176	0.15	0.01	0.00	[29,30]
bird	*Strix occidentalis*	17	423	0.0251	0.09	0.02	0.01	[31]
mammal	*Sus scrofa*	18	551	0.1040	0.16	0.02	0.01	[32,33]
mammal	*Capreolus capreolus*	13	371	0.1489	0.14	0.05	0.03	[34,35]
mammal	*Pekania pennanti*	34	722	0.0685	0.10	0.02	0.01	[36,37]
mammal	*Nyctalus leisleri*	14	183	0.0190	0.08	NA	NA	[38,39]
mammal	*Myotis lucifugus*	15	735	0.0005	0.05	NA	NA	[40,41]
mammal	*Vicugna vicugna*	14	374	0.1498	0.16	0.06	0.03	[42,43]
mammal	*Tamiasciurus douglasii*	14	186	0.0305	0.10	0.01	0.01	[44,45]
mammal	*Tamiasciurus hudsonicus*	12	188	0.0525	0.12	NA	NA	[44,45]

mammal	*Lepus americanus*	39	853	0.1524	0.19	0.05	0.03	[46,47]
mammal	*Ursus arctos*	16	831	0.1055	0.10	0.06	0.03	[48,49]
mammal	*Ursus maritimus*	11	318	0.0435	0.07	0.02	0.01	[48,49]
mammal	*Myotis lucifugus*	21	1054	0.0145	0.19	0.02	0.00	[50,51]
mammal	*Ovis canadensis*	14	579	0.1102	0.08	0.04	0.03	[52,53]
mammal	*Meles meles*	30	675	0.1721	0.18	0.05	0.04	[54,55]
mammal	*Microtus arvalis*	53	855	0.0064	0.09	0.00	0.00	[56,57]
mammal	*Canis latrans*	41	303	0.0236	0.10	0.01	0.00	[58,59]
mammal	*Rousettus aegyptiacus*	34	490	0.0705	0.11	0.02	0.01	[60,61]
mammal	*Myotis lucifugus*	29	1310	0.0000	0.05	0.00	NA	[62,63]
mammal	*Myotis septentrionalis*	15	896	0.0032	0.12	0.02	0.00	[62,63]
mammal	*Martes americana*	29	653	0.0217	0.05	0.01	0.01	[64,65]
mammal	*Lemmus lemmus*	13	276	0.0304	0.10	0.01	0.01	[66,67]
mammal	*Odocoileus hemionus*	60	1714	0.1173	0.15	0.03	0.03	[68,69]
mammal	*Rangifer tarandus*	18	634	0.0319	0.07	0.01	0.00	[70,71]
mammal	*Lynx canadensis*	28	702	0.0097	0.06	0.01	0.00	[72,73]
mammal	*Felis silvestris*	15	620	0.0841	0.16	0.04	0.02	[74,75]
mammal	*Alces alces*	16	694	0.0429	0.14	0.03	0.01	[76,77]
mammal	*Ursus maritimus*	14	2232	0.0332	0.09	0.01	0.01	[78,79]
mammal	*Ursus americanus*	28	504	0.1104	0.18	0.05	0.03	[80,81]
mammal	*Myotis escalerai*	15	442	0.0378	0.07	0.03	0.01	[82,83]
mammal	*Lynx rufus*	52	1646	0.0600	0.10	0.03	0.01	[84,85]
mammal	*Odocoileus virginianus*	64	2069	0.0081	0.10	0.01	0.00	[86,87]
mammal	*Rangifer tarandus*	27	508	0.0752	0.11	0.02	0.01	[88,89]
mammal	*Rhinolophus ferrumequinum*	27	950	0.0339	0.07	0.01	0.01	[90,91]
mammal	*Rangifer tarandus*	27	802	0.0676	0.14	0.02	0.01	[92,93]
mammal	*Miniopterus schreibersii*	22	312	0.0548	0.09	0.01	0.01	[94,95]
mammal	*Sorex antinorii*	17	213	0.0546	0.14	0.03	0.01	[96,97]
mammal	*Cervus elaphus*	27	638	0.1654	0.18	0.09	0.04	[98,99]
reptile	*Gopherus polyphemus*	46	933	0.2153	0.19	0.06	0.04	[100,101]
reptile	*Amblyrhynchus cristatus*	13	467	0.0860	0.09	NA	NA	[102,103]
reptile	*Liolaemus tenius*	15	127	0.1883	0.26	0.08	0.04	[104,105]

reptile	*Dipsosaurus dorsalis*	19	308	0.0725	0.09	0.02	0.01	[106,107]
reptile	*Uma inornata*	13	268	0.0507	0.10	0.02	0.01	[108,109]

References

1. Coster, S. S., Babbitt, K. J., Cooper, A. & Kovach, A. I. Data from: Limited influence of local and landscape factors on finescale gene flow in two pond-breeding amphibians. *Dryad* (2015) doi:10.5061/dryad.4903q.
2. Coster, S. S., Babbitt, K. J., Cooper, A. & Kovach, A. I. Limited influence of local and landscape factors on finescale gene flow in two pond-breeding amphibians. *Molecular Ecology* **24**, 742–758 (2015).
3. Duncan, S. I., Crespi, E. J., Mattheus, N. M. & Rissler, L. J. History matters more when explaining genetic diversity within the context of the core-periphery hypothesis. *Molecular Ecology* **24**, 4323–4336 (2015).
4. Duncan, S. I., Crespi, E. J., Mattheus, N. M. & Rissler, L. J. Data from: History matters more when explaining genetic diversity within the context of the core-periphery hypothesis. *Dryad* (2015) doi:10.5061/dryad.fp1k7.
5. Johnson, B. B., White, T. A., Phillips, C. A. & Zamudio, K. R. Data from: Asymmetric introgression in a spotted salamander hybrid zone. *Dryad* (2015) doi:10.5061/dryad.v23r2.
6. Johnson, B. B., White, T. A., Phillips, C. A. & Zamudio, K. R. Asymmetric Introgression in a Spotted Salamander Hybrid Zone. *Journal of Heredity* 608–617 (2015) doi:10.1093/jhered/esv042.
7. Metzger, G., Espindola, A., Waits, L. P. & Sullivan, J. Genetic structure across broad spatial and temporal scales: Rocky Mountain tailed frogs (*Ascaphus montanus*; Anura: Ascaphidae) in the inland temperate rainforest. *Journal of Heredity* 700–710 (2015) doi:10.1093/jhered/esv061.
8. Metzger, G., Espindola, A., Waits, L. P. & Sullivan, J. Data from: Genetic structure across broad spatial and temporal scales: Rocky Mountain tailed frogs (Ascaphus montanus;

Anura: Ascaphidae) in the inland temperate rainforest. *Dryad* (2015) doi:10.5061/dryad.2pb57.

9. Micheletti, S. J. & Storfer, A. Data from: An approach for identifying cryptic barriers to gene flow that limit species' geographic ranges. *Dryad* (2016) doi:10.5061/dryad.c6kj2.
10. Micheletti, S. J. & Storfer, A. An approach for identifying cryptic barriers to gene flow that limit species' geographic ranges. *Molecular Ecology* **26**, 490–504 (2017).
11. Peterman, W. E., Connette, G. M., Semlitsch, R. D. & Eggert, L. S. Ecological resistance surfaces predict fine-scale genetic differentiation in a terrestrial woodland salamander. *Molecular Ecology* **23**, 2402–2413 (2014).
12. Peterman, W. E., Connette, G. M., Semlitsch, R. D. & Eggert, L. S. Data from: Ecological resistance surfaces predict fine scale genetic differentiation in a terrestrial woodland salamander Dryad. *Dryad* (2014) doi:10.5061/dryad.m4f17.
13. Richardson, J. L. Data from: Divergent landscape effects on population connectivity in two co-occurring amphibian species. *Dryad* (2012) doi:10.5061/dryad.51b94.
14. Richardson, J. L. Divergent landscape effects on population connectivity in two co-occurring amphibian species. *Molecular Ecology* **21**, 4437–4451 (2012).
15. Richmond, J. Q., Barr, K. R., Backlin, A. R., Vandergast, A. G. & Fisher, R. N. Evolutionary dynamics of a rapidly receding southern range boundary in the threatened California Red-Legged Frog (Rana draytonii). *Evolutionary Applications* **6**, 808–822 (2013).
16. Richmond, J. Q., Barr, K. R., Backlin, A. R., Vandergast, A. G. & Fisher, R. N. Data from: Evolutionary dynamics of a rapidly receding southern range boundary in the threatened California Red-Legged Frog (Rana draytonii). *Dryad* (2013).

17. Rovito, S. M. & Schoville, S. D. Testing models of refugial isolation, colonization and population connectivity in two species of montane salamanders. *Heredity* **119**, 265–274 (2017).

18. Rovito, S. M. & Schoville, S. D. Data from: Testing models of refugial isolation, colonization and population connectivity in two species of montane salamanders. *Dryad* (2017) doi:10.5061/dryad.c197n.

19. Adams, R. V. & Burg, T. M. Data from: Influence of ecological and geological features on rangewide patterns of genetic structure in a widespread passerine. *Heredity* (2014) doi:10.5061/dryad.k086v.

20. Adams, R. V. & Burg, T. M. Influence of ecological and geological features on rangewide patterns of genetic structure in a widespread passerine. *Heredity* **114**, 143–154 (2015).

21. Barr, K. R. *et al.* Habitat fragmentation in coastal southern California disrupts genetic connectivity in the cactus wren (Campylorhynchus brunneicapillus). *Molecular Ecology* **24**, 2349–2363 (2015).

22. Barr, K. R. *et al.* Data from: Habitat fragmentation in coastal southern California disrupts genetic connectivity in the Cactus Wren (Campylorhynchus brunneicapillus). *Molecular Ecology* (2015) doi:10.5061/dryad.j5h92.

23. Farrington, H. L., Lawson, L. P., Clark, C. M. & Petren, K. The Evolutionary History of Darwin's Finches: Speciation, Gene Flow, and Introgression in a Fragmented Landscape. *Evolution* **68**, 2932–2944 (2014).

24. Farrington, H. L., Lawson, L. P., Clark, C. M. & Petren, K. Data from: The evolutionary history of Darwin's finches: speciation, gene flow, and introgression in a fragmented landscape. 227028 bytes (2014) doi:10.5061/DRYAD.J92FS.

25. Galligan, T. H. *et al.* Panmixia supports divergence with gene flow in Darwin's small ground finch, Geospiza fuliginosa, on Santa Cruz, Galápagos Islands. *Molecular Ecology* **21**, 2106–2115 (2012).

26. Galligan, T. H. *et al.* Data from: Panmixia supports divergence with gene flow in Darwin's small ground finch, Geospiza fuliginosa, on Santa Cruz, Galápagos Islands. 45489 bytes (2012) doi:10.5061/DRYAD.4SB063BN.

27. Gowen, F. C. *et al.* Speciation in Western Scrub-Jays, Haldane's rule, and genetic clines in secondary contact. *BMC Evolutionary Biology* **14**, 1–15 (2014).

28. Gowen, F. C. *et al.* Data from: Speciation in Western Scrub-Jays, Haldane's rule, and genetic clines in secondary contact. *BMC Evolutionary Biology* (2014) doi:10.5061/dryad.57f48.

29. Lait, L. A. & Burg, T. M. When east meets west: Population structure of a high-latitude resident species, the boreal chickadee (Poecile hudsonicus). *Heredity* **111**, 321–329 (2013).

30. Lait, L. A. & Burg, T. M. Data from: When east meets west: population structure of a high-latitude resident species, the boreal chickadee (Poecile hudsonicus). *Heredity* (2013) doi:10.5061/dryad.82hs7.

31. Miller, M. P., Davis, R. J., Mullins, T. D., Haig, S. M. & Forsman, E. D. Microsatellite markers, habitat quality, and sample location data for Northern Spotted Owls (Strix occidentalis caurina). (2017) doi:10.5066/F7J67FVW.

32. Alexandri, P. *et al.* Distinguishing migration events of different timing for wild boar in the Balkans. *Journal of Biogeography* **44**, 259–270 (2017).

33. Alexandri, P. *et al.* Data from: Distinguishing migration events of different timing for wild boar in the Balkans. 38412420 bytes (2017) doi:10.5061/DRYAD.T722H.

34. Baker, K. H. & Rus Hoelzel, A. Data from: Evolution of population genetic structure of the British roe deer by natural and anthropogenic processes (Capreolus capreolus). 131072 bytes (2013) doi:10.5061/DRYAD.V90P5.

35. Baker, K. H. & Rus Hoelzel, A. Evolution of population genetic structure of the British roe deer by natural and anthropogenic processes (*Capreolus capreolus*). *Ecol Evol* **3**, 89–102 (2013).

36. Bertrand, P., Bowman, J., Dyer, R., Manseau, M. & PJ, W. Sex-specific graphs: Relating group-specific topology to demographic and landscape data. *Molecular Ecology* **26**, 3898–3912 (2017).

37. Bertrand, P., Bowman, J., Dyer, R., Manseau, M. & PJ, W. Data from: Sex-specific graphs: Relating group-specific topology to demographic and landscape data. *Molecular Ecology* (2017) doi:10.5061/dryad.167d5.

38. Boston, E. S. M., Montgomery, W. I., Hynes, R. & Prodöhl, P. A. New insights on postglacial colonization in western Europe: the phylogeography of the Leisler's bat (*Nyctalus leisleri*). *Proc. R. Soc. B.* **282**, 20142605 (2015).

39. Boston, E. S. M., Montgomery, W. I., Hynes, R. & Prodöhl, P. A. Data from: New insights on postglacial colonisation in Western Europe: the phylogeography of the Leisler's bat (Nyctalus leisleri). 34926 bytes (2015) doi:10.5061/DRYAD.6G6R6.

40. Burns, L. E., Frasier, T. R. & Broders, H. G. Genetic connectivity among swarming sites in the wide ranging and recently declining little brown bat (Myotis lucifugus). *Ecology and Evolution* **4**, 4130–4149 (2014).

41. Burns, L. E., Frasier, T. R. & Broders, H. G. Data from: Genetic connectivity among swarming sites in the wide ranging and recently declining little brown bat (Myotis lucifugus). *Ecology and Evolution* (2014) doi:10.5061/dryad.hs37s.

42. Casey, C. S. *et al.* Comparing genetic diversity and demographic history in co-distributed wild South American camelids. *Heredity* **121**, 387–400 (2018).

43. Casey, C. S. *et al.* Data from: Comparing genetic diversity and demographic history in co-distributed wild South American camelids. 59614 bytes (2018) doi:10.5061/DRYAD.G8D77FT.

44. Chavez, A. S., Saltzberg, C. J. & Kenagy, G. J. Genetic and phenotypic variation across a hybrid zone between ecologically divergent tree squirrels (Tamiasciurus). *Molecular Ecology* **20**, 3350–3366 (2011).

45. Chavez, A. S., Saltzberg, C. J. & Kenagy, G. J. Data from: Genetic and phenotypic variation across a hybrid zone between ecologically divergent tree squirrels (Tamiasciurus). *Molecular Ecology* (2011) doi:10.5061/dryad.195qg.

46. Cheng, E., Hodges, K. E., Melo-Ferreira, J., Alves, P. C. & Mills, L. S. Conservation implications of the evolutionary history and genetic diversity hotspots of the snowshoe hare. *Molecular Ecology* **23**, 2929–2942 (2014).

47. Cheng, E., Hodges, K. E., Melo-Ferreira, J., Alves, P. C. & Mills, L. S. Data from: Conservation implications of the evolutionary history and genetic diversity hotspots of the snowshoe hare. *Molecular Ecology* (2014) doi:10.5061/dryad.dh63p.

48. Cronin, M. A. & MacNeil, M. D. Genetic relationships of extant brown bears (Ursus arctos) and polar bears (Ursus maritimus). *Journal of Heredity* **103**, 873–881 (2012).

49. Cronin, M. A. & MacNeil, M. D. Data from: Genetic relationships of extant brown bears (Ursus arctos) and polar bears (Ursus maritimus). *Journal of Heredity* (2012) doi:10.5061/dryad.q30rt.

50. Davy, C. M. *et al.* Prelude to a panzootic: gene flow and immunogenetic variation in northern little brown myotis vulnerable to bat white-nose syndrome. *FACETS* **2**, 690–714 (2017).

51. Davy, C. M. *et al.* Data from: Prelude to a panzootic: gene flow and immunogenetic variation in northern little brown myotis vulnerable to bat white-nose syndrome. *FACETS* (2017) doi:10.5061/dryad.h7n25.

52. Epps, C. W., Crowhurst, R. S. & Nickerson, B. S. Assessing changes in functional connectivity in a desert bighorn sheep metapopulation after two generations. *Molecular Ecology* **27**, 2334–2346 (2018).

53. Epps, C. W., Crowhurst, R. S. & Nickerson, B. S. Data from: Assessing changes in functional connectivity in a desert bighorn sheep metapopulation after two generations. *Molecular Ecology* (2018) doi:10.5061/dryad.mp71t50.

54. Frantz, A. C. *et al.* Revisiting the phylogeography and demography of European badgers (Meles meles) based on broad sampling, multiple markers and simulations. *Heredity* **113**, 443–453 (2014).

55. Frantz, A. C. *et al.* Data from: Re-visiting the phylogeography and demography of European badgers (Meles meles) based on broad sampling, multiple markers and simulations. 140089 bytes (2014) doi:10.5061/DRYAD.5NM5G.

56. Gauffre, B. *et al.* Short-term variations in gene flow related to cyclic density fluctuations in the common vole. *Molecular Ecology* **23**, 3214–3225 (2014).

57. Gauffre, B. *et al.* Data from: Short-term variations in gene flow related to cyclic density fluctuations in the common vole. 197880 bytes (2014) doi:10.5061/DRYAD.JF7SN.

58. Heppenheimer, E. *et al.* Demographic history influences spatial patterns of genetic diversityin recently expanded coyote (Canis latrans) populations. *Heredity* 1–13 (2017) doi:10.1038/s41437-017-0014-5.

59. Heppenheimer, E. *et al.* Data from: Demographic history influences spatial patterns of genetic diversity in recently expanded coyote (Canis latrans) populations. 92698 bytes (2017) doi:10.5061/DRYAD.2T965.

60. Hulva, P. *et al.* Data from: Environmental margin and island evolution in Middle Eastern populations of the Egyptian fruit bat. 228070 bytes (2012) doi:10.5061/DRYAD.K68K8.

61. Hulva, P. *et al.* Environmental margin and island evolution in M iddle E astern populations of the E gyptian fruit bat. *Molecular Ecology* **21**, 6104–6116 (2012).

62. Johnson, L. N. L. *et al.* Population Genetic Structure Within and among Seasonal Site Types in the Little Brown Bat (Myotis lucifugus) and the Northern Long-Eared Bat (M. septentrionalis). *PLOS ONE* **10**, 1–18 (2015).

63. Johnson, L. N. L. *et al.* Data from: Population genetic structure within and among seasonal site types in the little brown bat (Myotis lucifugus) and the northern long-eared bat (M. septentrionalis). *PLOS ONE* (2015) doi:10.5061/dryad.47nm0.

64. Koen, E. L., Bowman, J. & Wilson, P. J. Data from: Node-based measures of connectivity in genetic networks. *Molecular Ecology Resources* (2015) doi:10.5061/dryad.4tg23.

65. Koen, E. L., Bowman, J. & Wilson, P. J. Node-based measures of connectivity in genetic networks. *Molecular Ecology Resources* **16**, 69–79 (2016).

66. Lagerholm, V. K. *et al.* Data from: Run to the hills: gene flow among mountain areas leads to low genetic differentiation in the Norwegian lemming. 70687 bytes (2016) doi:10.5061/DRYAD.KR966.

67. Lagerholm, V. K. *et al.* Run to the hills: gene flow among mountain areas leads to low genetic differentiation in the Norwegian lemming. *Biological Journal of the Linnean Society* **121**, 1–14 (2017).

68. Latch, E. K., Reding, D. M., Heffelfinger, J. R., Alcalá-Galván, C. H. & Rhodes, O. E. Range-wide analysis of genetic structure in a widespread, highly mobile species (Odocoileus hemionus) reveals the importance of historical biogeography. *Molecular Ecology* **23**, 3171–3190 (2014).

69. Latch, E. K., Reding, D. M., Heffelfinger, J. R., Alcalá-Galván, C. H. & Rhodes, O. E. Data from: Range-wide analysis of genetic structure in a widespread, highly mobile species (Odocoileus hemionus) reveals the importance of historical biogeography. *Molecular Ecology* (2014) doi:10.5061/dryad.ns2jn.

70. Mager, K. H., Colson, K. E., Groves, P. & Hundertmark, K. J. Population structure over a broad spatial scale driven by nonanthropogenic factors in a wide-ranging migratory mammal, Alaskan caribou. *Molecular Ecology* **23**, 6045–6057 (2014).

71. Mager, K. H., Colson, K. E., Groves, P. & Hundertmark, K. J. Data from: Population structure over a broad spatial scale driven by non-anthropogenic factors in a wide-ranging migratory mammal, Alaskan caribou. *Molecular Ecology* (2014) doi:10.5061/dryad.3hp5v.

72. Marrotte, R. R. *et al.* Multi-species genetic connectivity in a terrestrial habitat network. *Movement Ecology* **5**, 1–11 (2017).

73. Marrotte, R. R. *et al.* Data from: Multi-species genetic connectivity in a terrestrial habitat network. *Movement Ecology* (2017) doi:10.5061/dryad.qn4kq.

74. Mattucci, F., Oliveira, R., Lyons, L. A., Alves, P. C. & Randi, E. European wildcat populations are subdivided into five main biogeographic groups: consequences of Pleistocene climate changes or recent anthropogenic fragmentation? *Ecology and Evolution* **6**, 3–22 (2016).

75. Mattucci, F., Oliveira, R., Lyons, L. A., Alves, P. C. & Randi, E. Data from: European wildcat populations are subdivided into five main biogeographic groups: consequences of Pleistocene climate changes or recent anthropogenic fragmentation? 681984 bytes (2016) doi:10.5061/DRYAD.KB13M.

76. Niedziałkowska, M. *et al.* Data from: The contemporary genetic pattern of European moose is shaped by postglacial recolonization, bottlenecks, and the geographical barrier of the Baltic Sea. 102689 bytes (2015) doi:10.5061/DRYAD.0TC6Q.

77. Niedziałkowska, M. *et al.* The contemporary genetic pattern of European moose is shaped by postglacial recolonization, bottlenecks, and the geographical barrier of the Baltic Sea. *Biol. J. Linn. Soc.* **117**, 879–894 (2016).

78. Peacock, E. *et al.* Implications of the circumpolar genetic structure of polar bears for their conservation in a rapidly warming Arctic. *PLoS ONE* **10**, 1–30 (2015).

79. Peacock, E. *et al.* Data from: Implications of the circumpolar genetic structure of polar bears for their conservation in a rapidly warming Arctic. 595858 bytes (2015) doi:10.5061/DRYAD.V2J1R.

80. Puckett, E. E., Etter, P. D., Johnson, E. A. & Eggert, L. S. Phylogeographic analyses of American black bears (Ursus americanus) suggest four glacial refugia and complex patterns of postglacial admixture. *Molecular Biology and Evolution* **32**, 2338–2350 (2015).

81. Puckett, E. E., Etter, P. D., Johnson, E. A. & Eggert, L. S. Data from: Phylogeographic analyses of American black bears (Ursus americanus) suggest four glacial refugia and complex patterns of post-glacial admixture. *Molecular Biology and Evolution* (2015) doi:10.5061/dryad.dc02b.

82. Razgour, O., Salicini, I., Ibáñez, C., Randi, E. & Juste, J. Unravelling the evolutionary history and future prospects of endemic species restricted to former glacial refugia. *Molecular Ecology* **24**, 5267–5283 (2015).

83. Razgour, O., Salicini, I., Ibáñez, C., Randi, E. & Juste, J. Data from: Unravelling the evolutionary history and future prospects of endemic species restricted to former glacial refugia. 2802205 bytes (2015) doi:10.5061/DRYAD.V1V47.

84. Reding, D. M., Bronikowski, A. M., Johnson, W. E. & Clark, W. R. Pleistocene and ecological effects on continental-scale genetic differentiation in the bobcat (Lynx rufus). *Molecular Ecology* **21**, 3078–3093 (2012).

85. Reding, D. M., Bronikowski, A. M., Johnson, W. E. & Clark, W. R. Data from: Pleistocene and ecological effects on continental-scale genetic differentiation in the bobcat (Lynx rufus). *Molecular Ecology* (2012) doi:10.5061/dryad.d3t16pd2.

86. Robinson, S. J., Samuel, M. D., Lopez, D. L. & Shelton, P. The walk is never random: Subtle landscape effects shape gene flow in a continuous white-tailed deer population in the Midwestern United States. *Molecular Ecology* **21**, 4190–4205 (2012).

87. Robinson, S. J., Samuel, M. D., Lopez, D. L. & Shelton, P. Data from: The walk is never random: subtle landscape effects shape gene flow in a continuous white-tailed deer population in the Midwestern United States. *Molecular Ecology* (2012) doi:10.5061/dryad.p7639.

88. Serrouya, R. *et al.* Population size and major valleys explain microsatellite variation better than taxonomic units for caribou in western Canada. *Molecular Ecology* **21**, 2588–2601 (2012).

89. Serrouya, R. *et al.* Data from: Population size and major valleys explain microsatellite variation better than taxonomic units for caribou in western Canada. 114056 bytes (2012) doi:10.5061/DRYAD.250C3S47.

90. Tournayre, O. *et al.* Integrating population genetics to define conservation units from the core to the edge of *Rhinolophus ferrumequinum* western range. *Ecology and Evolution* **9**, 12272–12290 (2019).

91. Tournayre, O. *et al.* Data from: Integrating population genetics to define conservation units from the core to the edge of Rhinolophus ferrumequinum western range. 104872 bytes (2020) doi:10.5061/DRYAD.R44T5DK.

92. Weckworth, B. V., Musiani, M., McDevitt, A. D., Hebblewhite, M. & Mariani, S. Reconstruction of caribou evolutionary history in Western North America and its implications for conservation. *Molecular Ecology* **21**, 3610–3624 (2012).

93. Weckworth, B. V., Musiani, M., McDevitt, A. D., Hebblewhite, M. & Mariani, S. Data from: Reconstruction of caribou evolutionary history in Western North America and its implications for conservation. *Molecular Ecology* (2012) doi:10.5061/dryad.gn22271h.

94. Witsenburg, F. *et al.* How a haemosporidian parasite of bats gets around: the genetic structure of a parasite, vector and host compared. *Molecular Ecology* **24**, 926–940 (2015).

95. Witsenburg, F. *et al.* Data from: How a haemosporidian parasite of bats gets around: the genetic structure of a parasite, vector and host compared. 418639 bytes (2015) doi:10.5061/DRYAD.2M1P0.

96. Yannic, G., Basset, P., Büchi, L., Hausser, J. & Broquet, T. Data from: Scale-specific sex-biased dispersal in the Valais shrew unveiled by genetic variation on the Y chromosome, autosomes, and mitochondrial DNA. 86016 bytes (2011) doi:10.5061/DRYAD.8K3423TS.

97. Yannic, G., Basset, P., Büchi, L., Hausser, J. & Broquet, T. Scale-specific sex-biased dispersal in the Valais shrew unveiled by genetic variation on the Y chromosome, autosomes, and mitochondrial DNA. *Evolution* **66**, 1737–1750 (2012).

98. Zachos, F. E. *et al.* Genetic Structure and Effective Population Sizes in European Red Deer (Cervus elaphus) at a Continental Scale: Insights from Microsatellite DNA. *Journal of Heredity* **107**, 318–326 (2016).

99. Zachos, F. E. *et al.* Data from: Genetic structure and effective population sizes in European red deer (Cervus elaphus) at a continental scale: insights from microsatellite DNA. 98504 bytes (2016) doi:10.5061/DRYAD.1V6P1.

100. Gaillard, D. *et al.* Range-wide and regional patterns of population structure and genetic diversity in the gopher tortoise. *Journal of Fish and Wildlife Management* **8**, 497–512 (2017).

101. Gaillard, D. *et al.* Data from: Range-wide and regional patterns of population structure and genetic diversity in the gopher tortoise. 2527036 bytes (2017) doi:10.5061/DRYAD.NK064.

102. MacLeod, A. *et al.* Hybridization masks speciation in the evolutionary history of the Galápagos marine iguana. *Proceedings of the Royal Society B: Biological Sciences* **282**, 20150425 (2015).

103. MacLeod, A. *et al.* Data from: Hybridization masks speciation in the evolutionary history of the Galápagos marine iguana. 19278179 bytes (2015) doi:10.5061/DRYAD.PP6BM.

104. Muñoz-Mendoza, C. *et al.* Geography and past climate changes have shaped the evolution of a widespread lizard from the Chilean hotspot. *Molecular Phylogenetics and Evolution* **116**, 157–171 (2017).

105. Muñoz-Mendoza, C. *et al.* Data from: Geography and past climate changes have shaped the evolution of a widespread lizard from the Chilean hotspot. 273270 bytes (2017) doi:10.5061/DRYAD.JK183.

106. Valdivia-Carrillo, T., García-De León, F. J., Blázquez, Ma. C., Gutiérrez-Flores, C. & González Zamorano, P. Phylogeography and Ecological Niche Modeling of the Desert Iguana (Dipsosaurus dorsalis, Baird & Girard 1852) in the Baja California Peninsula. *Journal of Heredity* **108**, 640–649 (2017).

107. Valdivia-Carrillo, T., García-De León, F. J., Blázquez, Ma. C., Gutiérrez-Flores, C. & González Zamorano, P. Data from: Phylogeography and ecological niche modelling of the desert iguana (Dipsosaurus dorsalis, Baird & Girard 1852) in the Baja California Peninsula. 13137255 bytes (2017) doi:10.5061/DRYAD.6R7QN.

108. Vandergast, A. G. *et al.* Drifting to oblivion? Rapid genetic differentiation in an endangered lizard following habitat fragmentation and drought. *Diversity and Distributions* **22**, 344–357 (2016).

109. Vandergast, A. G. *et al.* Dryad Data -- Drifting to oblivion? Rapid genetic differentiation in an endangered lizard following habitat fragmentation and drought. (2016) doi:10.5061/dryad.30t5b.

Table S2. GDAR scaling exponents (z-values) for terrestrial vertebrates summarized across taxonomic groups (overall values) and for each taxonomic class. Means and standard deviations of z-values are given for allele counts (z_{AC}), allelic richness (z_{AR}), and gene diversity GDARs (z_{GD}).

	z_{AC}	z_{AR}	z_{GD}
overall	0.12 ± 0.05	0.03 ± 0.02	0.02 ± 0.02
amphibian	0.13 ± 0.06	0.04 ± 0.04	0.02 ± 0.03
bird	0.14 ± 0.03	0.02 ± 0.01	0.01 ± 0.00
mammal	0.11 ± 0.04	0.03 ± 0.02	0.01 ± 0.01
reptile	0.14 ± 0.08	0.04 ± 0.03	0.03 ± 0.02

Table S3. Model summaries testing whether F_{ST} or *z* values differ from zero across taxa. Columns are model coefficients and 95% confidence interval for each metric (F_{ST}, and *z* values estimated based on gene diversity, allelic richness, and allele count). Here, model intercepts were set to zero so slope coefficients estimate the mean value of the genetic metric per taxonomic class in a series of four models (one per response variable). Confidence intervals overlapping zero indicate that taxonomic means are indistinguishable from zero. Values for F_{ST} and z_{GD} metrics were greater than zero for amphibians, mammals, and reptiles, and indistinguishable from zero for birds. This pattern supports the expectation that flying species tend to have less genetically structured populations than walking species. This pattern was weaker for *z* values based on allelic richness and did not hold for *z* values based on allele count.

	F_{ST}	z_{GD}	z_{AR}	z_{AC}
amphibian	0.11 (0.06 – 0.16)	0.02 (0.01 – 0.03)	0.04 (0.02 – 0.05)	0.13 (0.10 – 0.16)
bird	0.04 (-0.01 – 0.10)	0.01 (-0.00 – 0.02)	0.02 (0.00 – 0.04)	0.14 (0.10 – 0.17)
mammal	0.07 (0.04 – 0.09)	0.01 (0.01 – 0.02)	0.03 (0.02 – 0.04)	0.12 (0.10 – 0.13)
reptile	0.13 (0.06 – 0.021)	0.03 (0.01 – 0.04)	0.04 (0.02 – 0.07)	0.16 (0.11 – 0.21)

Table S4. Comparison of area effect sizes between models with area alone, and global F_{ST} and an area*F_{ST} interaction as predictors. Effect sizes and standard errors are given for area, F_{ST}, and the interaction between area and F_{ST} where applicable. All effects were significant (p<0.05) in all models. The area effect is strongly reduced when F_{ST} and their interaction are included in the model. For gene diversity and allelic richness, when populations are panmictic (F_{ST} = 0), the effect of area is negligible (bold values). The coefficient for the interaction terms are orders of magnitude larger than the area effect, demonstrating that the effect of area is strongly mediated by population structure (see also Figure S2 for plotted interaction effects). We show the effect size of F_{ST} only for complete understanding of the magnitude of the interactive effect with area. We caution against causal interpretations of F_{ST} on genetic diversity because genetic diversity (alpha diversity) and genetic differentiation (beta diversity) reflect two measures of diversity that influence each other and are products of the same processes.

**genetic metric**	**model**	**area**	**F_{ST}**	**area*F_{ST} interaction**
gene diversity	area	0.01 ± 0.0003	--	--
	interaction	**-0.001 ± 0.0004**	-2.44 ± 0.11	0.21 ± 0.004
allelic richness	area	0.02 ± 0.0005	--	--
	interaction	**0.004 ± 0.0007**	-3.95 ± 0.24	0.31 ± 0.007
allele count	area	0.13 ± 0.001	--	--
	interaction	0.09 ± 0.001	-6.33 ± 0.46	0.50 ± 0.01

Table S5. Model summaries for relationships between F_{ST}, *z* values derived from allele count (zAC), allelic richness (zAR), and gene diversity (zGD), and predictor variables including: home range size (km^2), species range size (km^2), species body mass (g), and the area of the spatial extent of the sample locations in each dataset (km^2). All predictors were log-transformed prior to analysis. Because F_{ST} is bounded by 0 and 1, we used a beta regression and report pseudo-R^2 to summarize explained variation. *Z* values are not bounded by 0 and 1 thus we used linear regressions with normally distributed errors and report adjusted R^2 (R^2_{adj}), which corrects for the number of predictors included in the model, to summarize explained variation. Estimated slopes for each predictor are given with 95% confidence intervals.

	**predictor**	**estimate**	**95% CI**
**F_{ST}**	home range	-0.17	-0.31 – -0.02
pseudo R^2 = 0.58	range size	-0.58	-1.02 – -0.14
	body mass	0.32	0.17 – 0.47
	sampled area size	0.47	0.25 – 0.69
**zAC**	home range	-0.02	-0.03 – -0.01
R^2_{adj} = 0.36	range size	0.01	-0.02 – 0.03
	body mass	0.01	-0.00 – 0.02
	sampled area size	0.02	0.01 – 0.04
**zAR**	home range	-0.01	-0.01 – -0.00
R^2_{adj} = 0.42	range size	-0.02	-0.03 – -0.00
	body mass	0.01	0.00 – 0.01
	sampled area size	0.01	0.00 – 0.02
**zGD**	home range	-0.00	-0.01 – -0.00
R^2_{adj} = 0.45	range size	-0.01	-0.01 – -0.00
	body mass	0.00	0.00 – 0.01
	sampled area size	0.01	0.00 – 0.01

Table S6. F_{ST} estimates from the MacroPopGen database and associated trait data. Species on multiple rows represent data from multiple studies.

species	F_{ST}	mass (g)	species range size (km2)	home range size (km2)	sample area (km2)
Alces alces	0.14	461900.76	23096272.63	40.49	175366.25
Alces alces	0.11	461900.76	23096272.63	40.49	1771220.04
Bison bison	0.12	624577.07	140673.08	605.92	2570587.13
Ctenomys lami	0.28	240.00	11405.52	0.0006	3197.90
Ctenomys minutus	0.20	92.00	54952.67	0.0003	1164.51
Ctenomys torquatus	0.43	209.49	240794.29	0.0006	157533.70
Cynomys ludovicianus	0.12	797.05	1820824.74	0.0015	319.71
Cynomys ludovicianus	0.11	797.05	1820824.74	0.0015	280212.01
Cynomys ludovicianus	0.20	797.05	1820824.74	0.0015	280212.01
Cynomys parvidens	0.28	899.98	31805.77	0.0015	13315.57
Didelphis virginiana	0.07	2442.08	6033229.98	0.44	1098.05
Gulo gulo	0.07	12792.49	24394335.16	553.13	10655064.79
Lepus americanus	0.18	1568.42	9597254.59	0.08	19945447.02
Lontra canadensis	0.07	8087.42	19086449.76	0.17	191718.98
Lycalopex vetulus	0.03	4233.47	2052494.18	3.85	2323208.40
Lynx rufus	0.07	6374.47	9879513.02	19.88	9189982.82
Lynx rufus	0.10	6374.47	9879513.02	19.88	10394522.78
Lynx rufus	0.02	6374.47	9879513.02	19.88	168693.39
Marmota flaviventris	0.13	3709.73	1718256.72	0.04	159109.65
Martes americana	0.06	873.69	7637867.17	4.36	11660211.07
Martes americana	0.25	873.69	7637867.17	4.36	6760157.05
Microtus californicus	0.22	57.42	287643.44	0.002	76221.24
Neotamias ruficaudus	0.08	60.06	147850.63	0.01	72050.94
Ochotona princeps	0.22	157.63	894668.90	0.001	1434.21
Odocoileus virginianus	0.01	75901.25	14410637.40	1.39	1048733.76
Odocoileus virginianus	0.03	75901.25	14410637.40	1.39	522253.74
Ondatra zibethicus	0.18	991.31	14688570.91	0.01	11328640.61
Oreamnos americanus	0.19	72105.40	786933.83	39.50	4725770.76
Ovis canadensis	0.16	74644.87	532974.90	19.56	63031.27
Ovis canadensis	0.26	74644.87	532974.90	19.56	1837485.47
Ovis dalli	0.16	70194.13	816225.07	22.38	2537227.55
Panthera onca	0.05	83943.09	9026172.30	156.62	33531390.54
Panthera onca	0.11	83943.09	9026172.30	156.62	26001315.13
Pekania pennanti	0.14	3750.00	3711380.82	28.68	5743415.25
Peromyscus leucopus	0.04	18.07	5831351.77	0.002	64.76
Peromyscus leucopus	0.02	18.07	5831351.77	0.002	69760.20
Peromyscus maniculatus	0.16	19.98	13030881.64	0.0009	71652.19
Puma concolor	0.13	53954.05	21145719.52	210.26	530384.78
Puma concolor	0.09	53954.05	21145719.52	210.26	802285.37
Puma concolor	0.09	53954.05	21145719.52	210.26	49202.25
Puma concolor	0.09	53954.05	21145719.52	210.26	920939.26

Rangifer tarandus	0.35	109088.50	18660780.32	426.50	514089.62
Rangifer tarandus	0.04	109088.50	18660780.32	426.50	2002962.38
Rangifer tarandus	0.09	109088.50	18660780.32	426.50	1494784.44
Rangifer tarandus	0.13	109088.50	18660780.32	426.50	15220188.89
Rattus norvegicus	0.07	282.89	18825760.66	0.02	63.83
Tamias striatus	0.05	90.50	4197616.71	0.002	24743.56
Urocitellus brunneus	0.26	300.00	460.17	0.01	31682.52
Ursus americanus	0.24	110500.00	10469239.38	155.83	481369.34
Ursus americanus	0.11	110500.00	10469239.38	155.83	62483.39
Ursus americanus	0.19	110500.00	10469239.38	155.83	110521.75
Ursus arctos	0.16	196287.50	24216941.84	338.03	3104094.30
Ursus arctos	0.05	196287.50	24216941.84	338.03	706656.94
Ursus maritimus	0.03	371703.81	22605413.56	77331.93	9409086.03
Vicugna vicugna	0.14	47499.61	618702.83	0.03	469293.97
Vulpes velox	0.08	2088.00	599398.12	11.80	956737.27

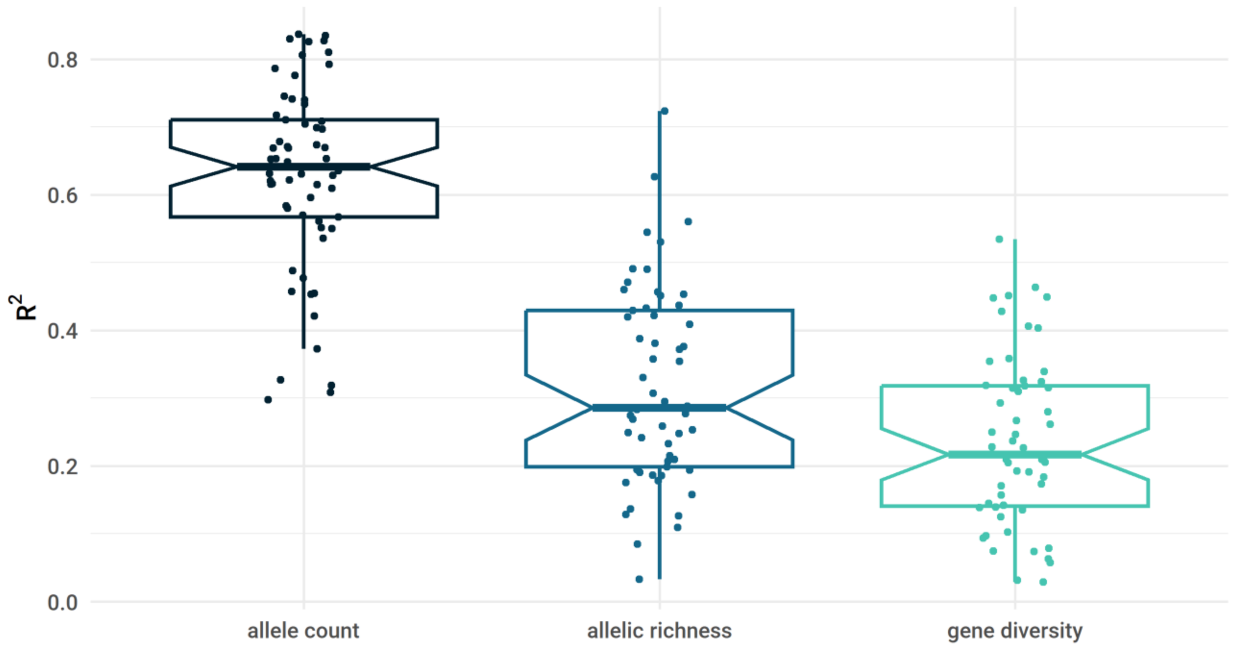

Figure S1. Box plots of variance in genetic diversity explained by area (R^2) for each genetic metric. Points are the R^2 of a simple linear model of the log-log relationship between each genetic metric (x axis) and area for each dataset. As expected, the variance in diversity explained by area is higher in genetic diversity metrics that are more strongly affected by sample size. This general pattern was consistent across all taxonomic groups.

Figure S2. Genetic diversity vs area plotted on a log-log scale for all 61 datasets. Red-shaded regions are 95% confidence intervals for the effect of area on genetic diversity. The slopes of these relationships are the scaling exponents *z* (values are reported in each plot title; they are NA when the relationship was not significant). Plots for allele count, allelic richness, and gene diversity are shown consecutively: note the genetic metric on the y axis. Point color denotes sample size, with lighter colors indicating greater numbers of individuals sampled.

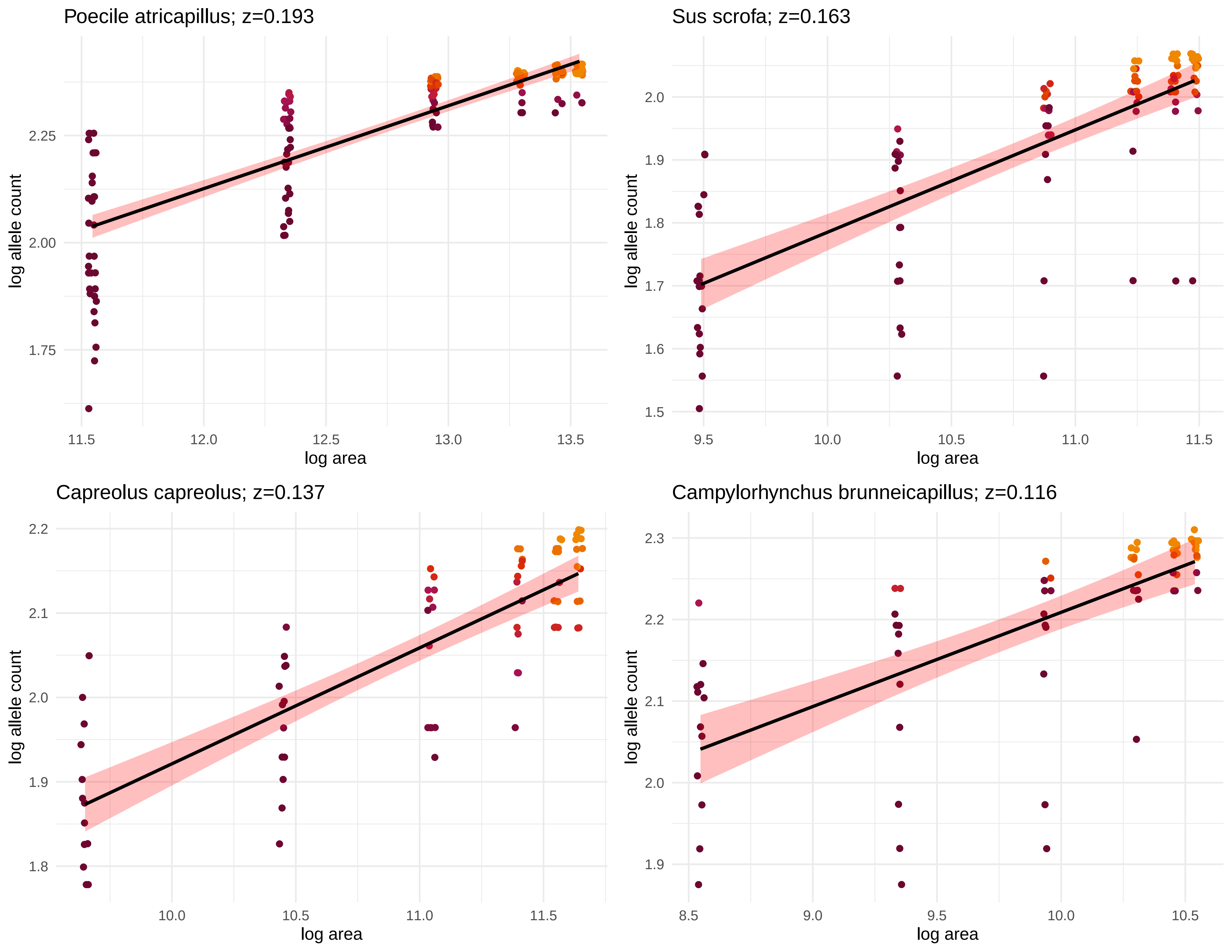
Poecile atricapillus; z=0.193
log allele count
2.25
2.00
1.75
11.5
12.0
12.5
13.0
13.5
log area
Sus scrofa; z=0.163
log allele count
2.0
1.9
1.8
1.7
1.6
1.5
9.5
10.0
10.5
11.0
11.5
log area
Capreolus capreolus; z=0.137
log allele count
2.2
2.1
2.0
1.9
1.8
10.0
10.5
11.0
11.5
log area
Campylorhynchus brunneicapillus; z=0.116
log allele count
2.3
2.2
2.1
2.0
1.9
8.5
9.0
9.5
10.0
10.5
log area

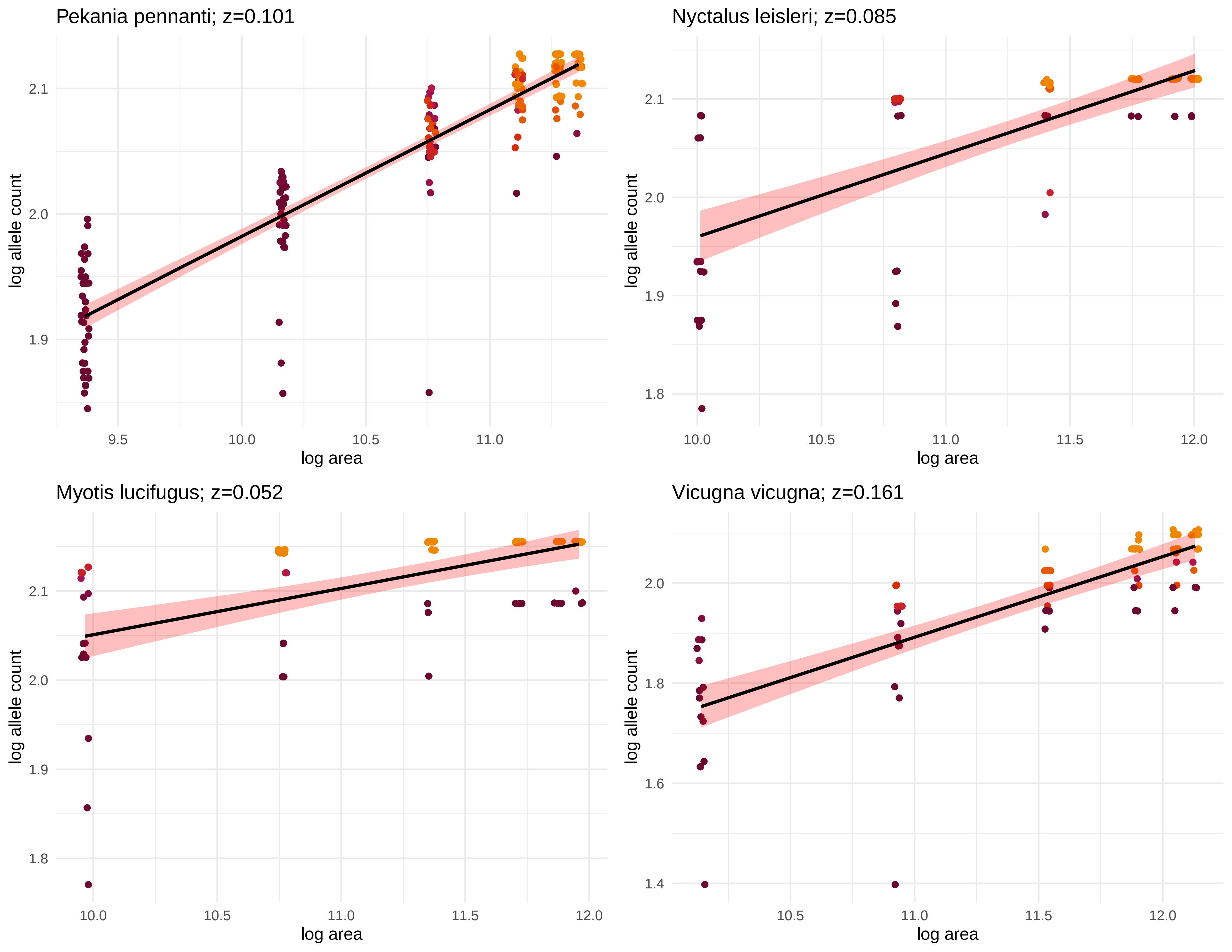
Pekania pennanti; z=0.101
Nyctalus leisleri; z=0.085
Myotis lucifugus; z=0.052
Vicugna vicugna; z=0.161
log allele count
log area

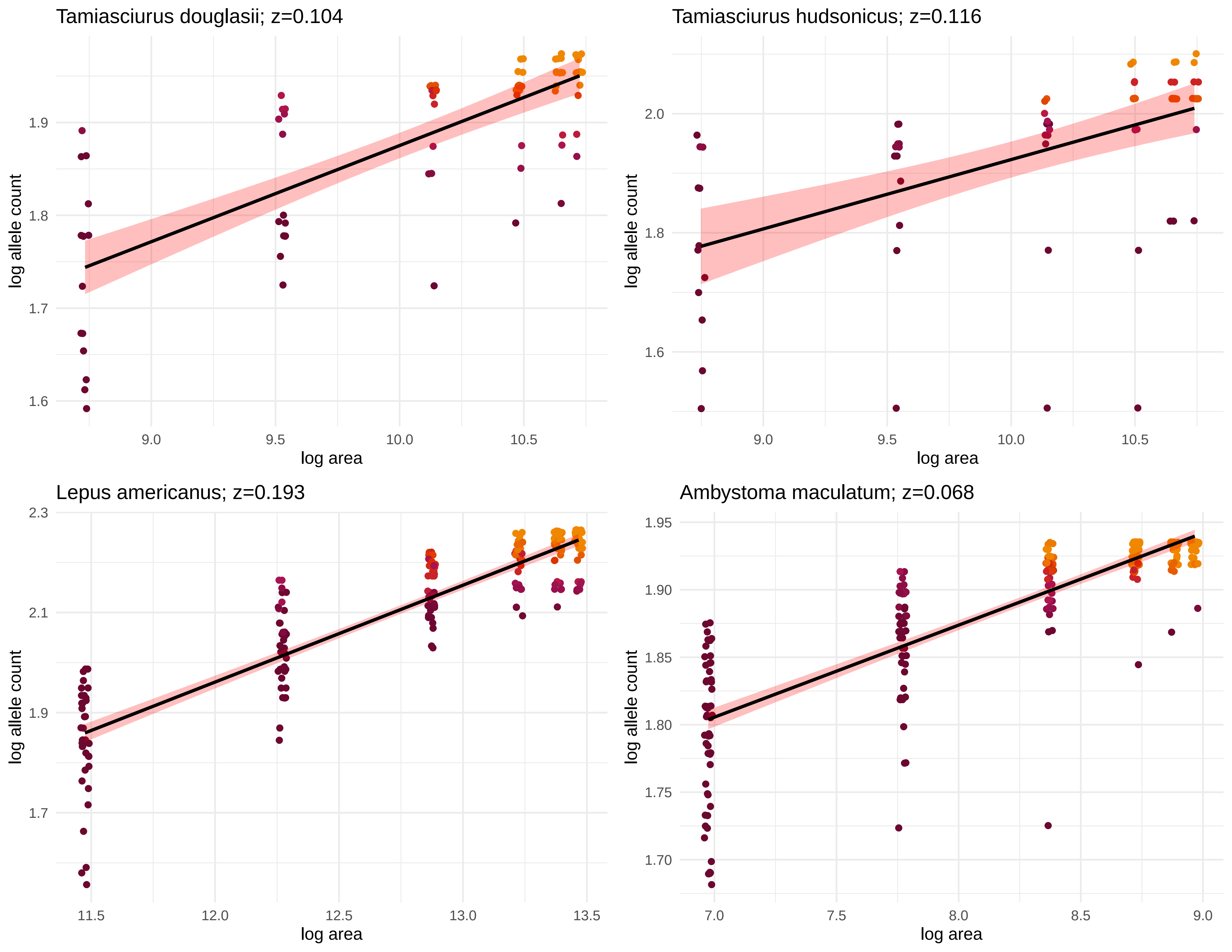
Tamiasciurus douglasii; z=0.104
log allele count
1.6
1.7
1.8
1.9
9.0
9.5
10.0
10.5
log area
Tamiasciurus hudsonicus; z=0.116
log allele count
1.6
1.8
2.0
9.0
9.5
10.0
10.5
log area
Lepus americanus; z=0.193
log allele count
1.7
1.9
2.1
2.3
11.5
12.0
12.5
13.0
13.5
log area
Ambystoma maculatum; z=0.068
log allele count
1.70
1.75
1.80
1.85
1.90
1.95
7.0
7.5
8.0
8.5
9.0
log area

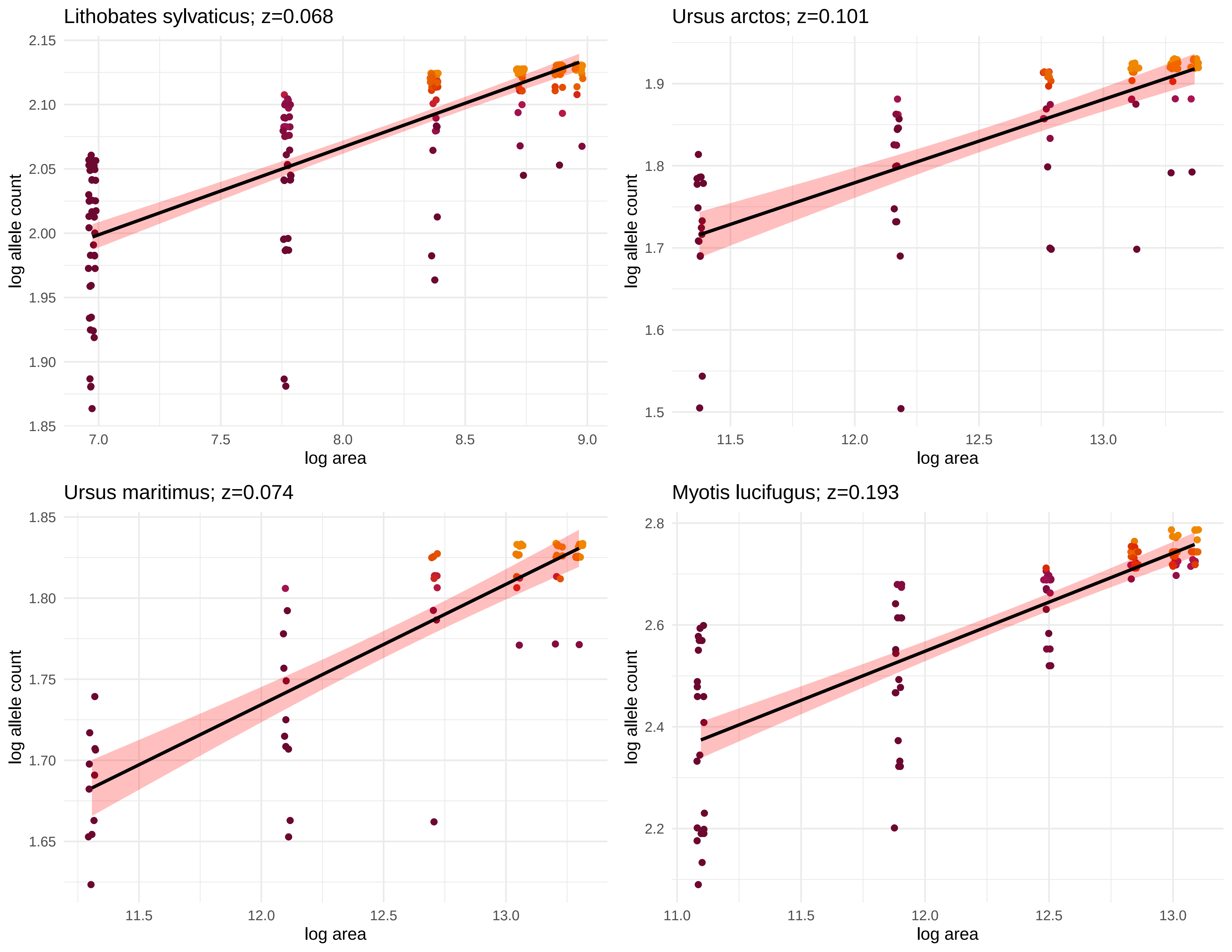

Lithobates sylvaticus; z=0.068
Ursus arctos; z=0.101
Ursus maritimus; z=0.074
Myotis lucifugus; z=0.193
log allele count
log area

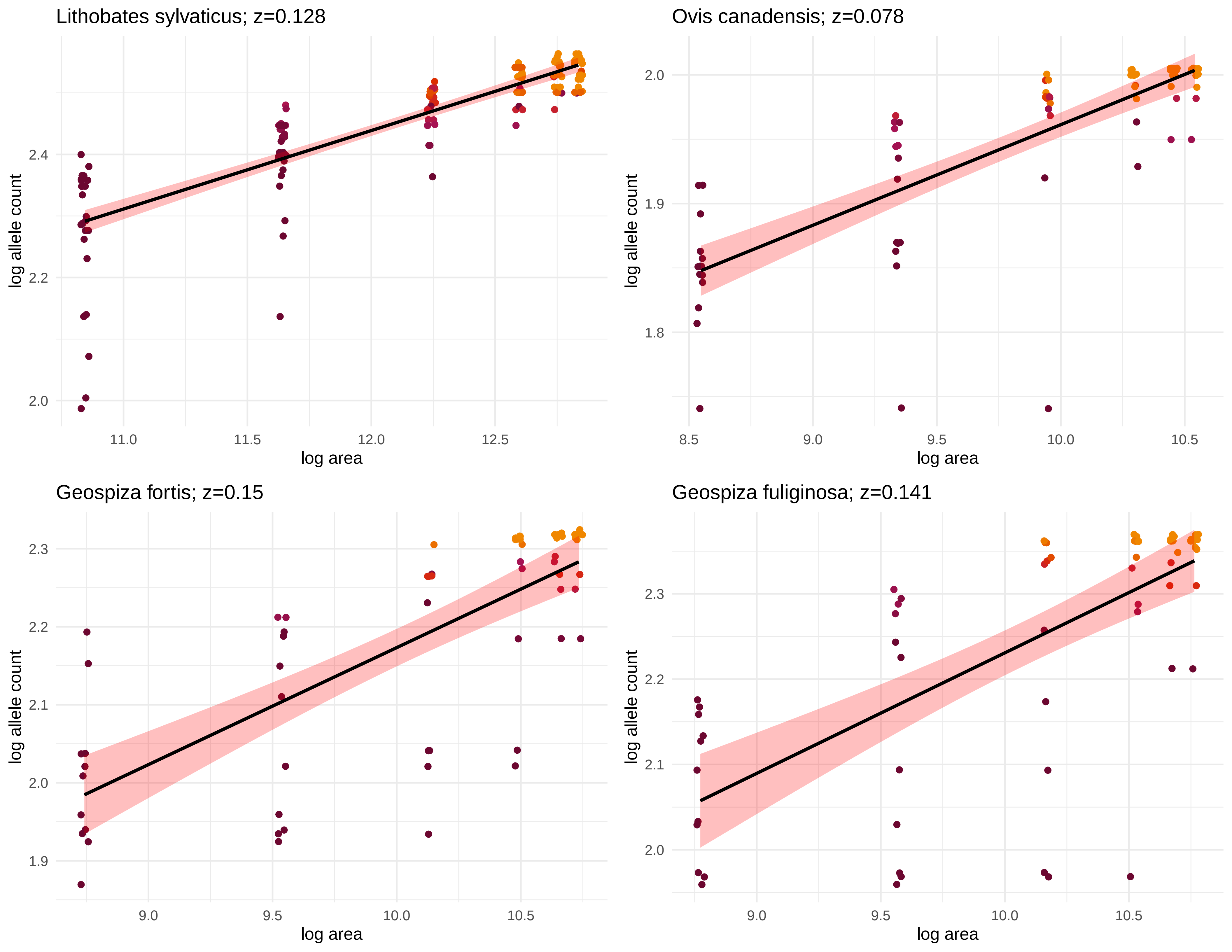

Lithobates sylvaticus; z=0.128
Ovis canadensis; z=0.078
Geospiza fortis; z=0.15
Geospiza fuliginosa; z=0.141
log allele count
log area

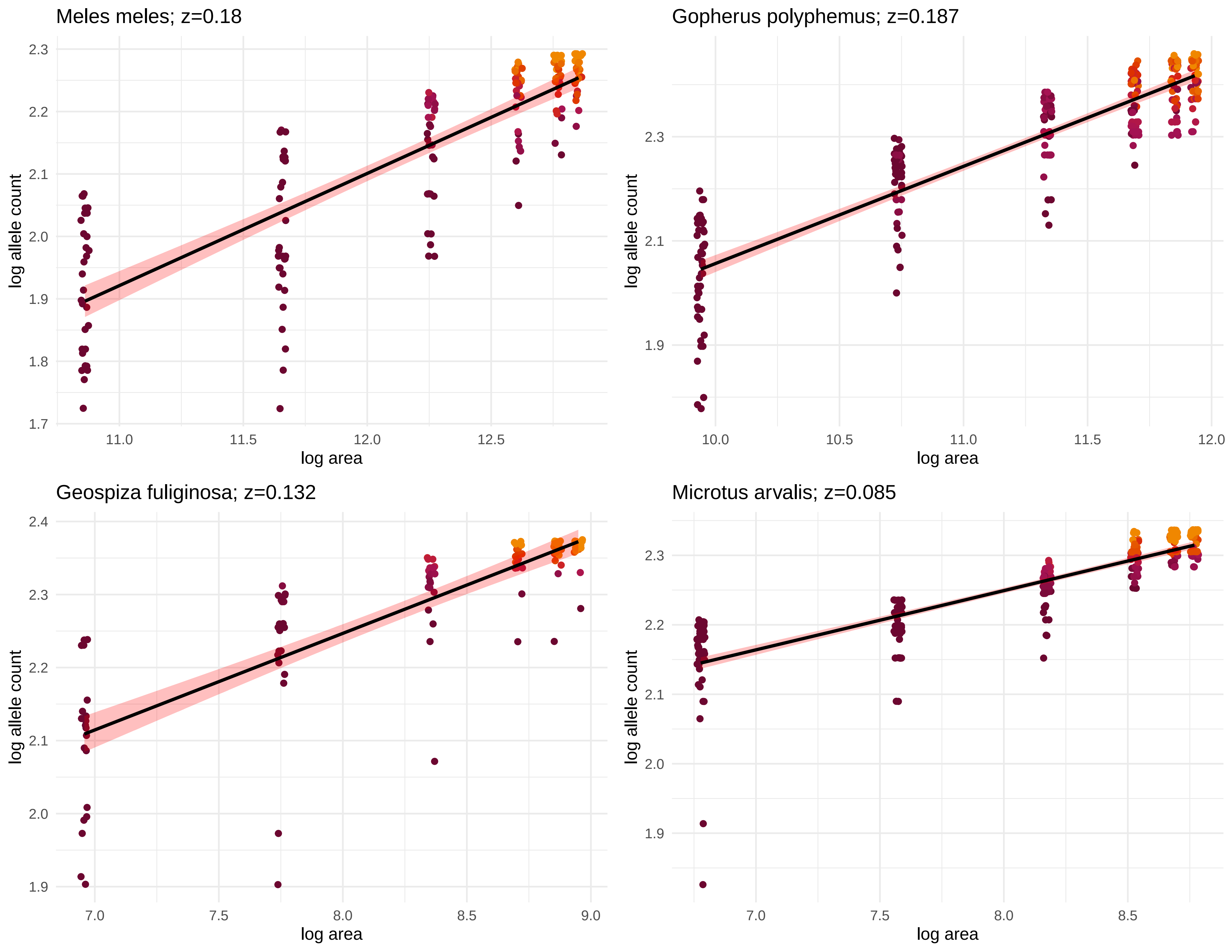

Meles meles; z=0.18
Gopherus polyphemus; z=0.187
Geospiza fuliginosa; z=0.132
Microtus arvalis; z=0.085
log allele count
log area

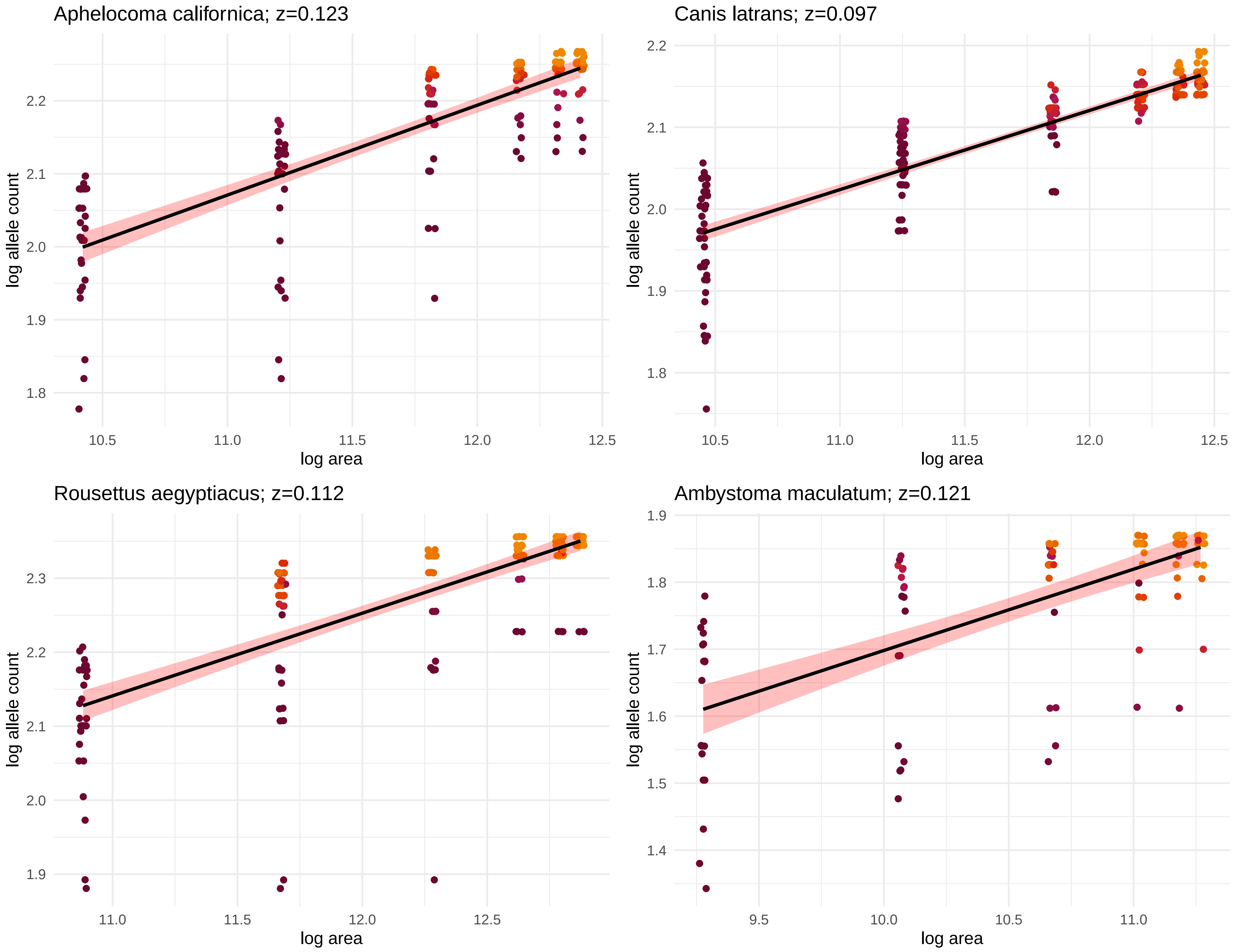
Aphelocoma californica; z=0.123
log allele count
2.2
2.1
2.0
1.9
1.8
10.5
11.0
11.5
12.0
12.5
log area
Canis latrans; z=0.097
log allele count
2.2
2.1
2.0
1.9
1.8
10.5
11.0
11.5
12.0
12.5
log area
Rousettus aegyptiacus; z=0.112
log allele count
2.3
2.2
2.1
2.0
1.9
11.0
11.5
12.0
12.5
log area
Ambystoma maculatum; z=0.121
log allele count
1.9
1.8
1.7
1.6
1.5
1.4
9.5
10.0
10.5
11.0
log area

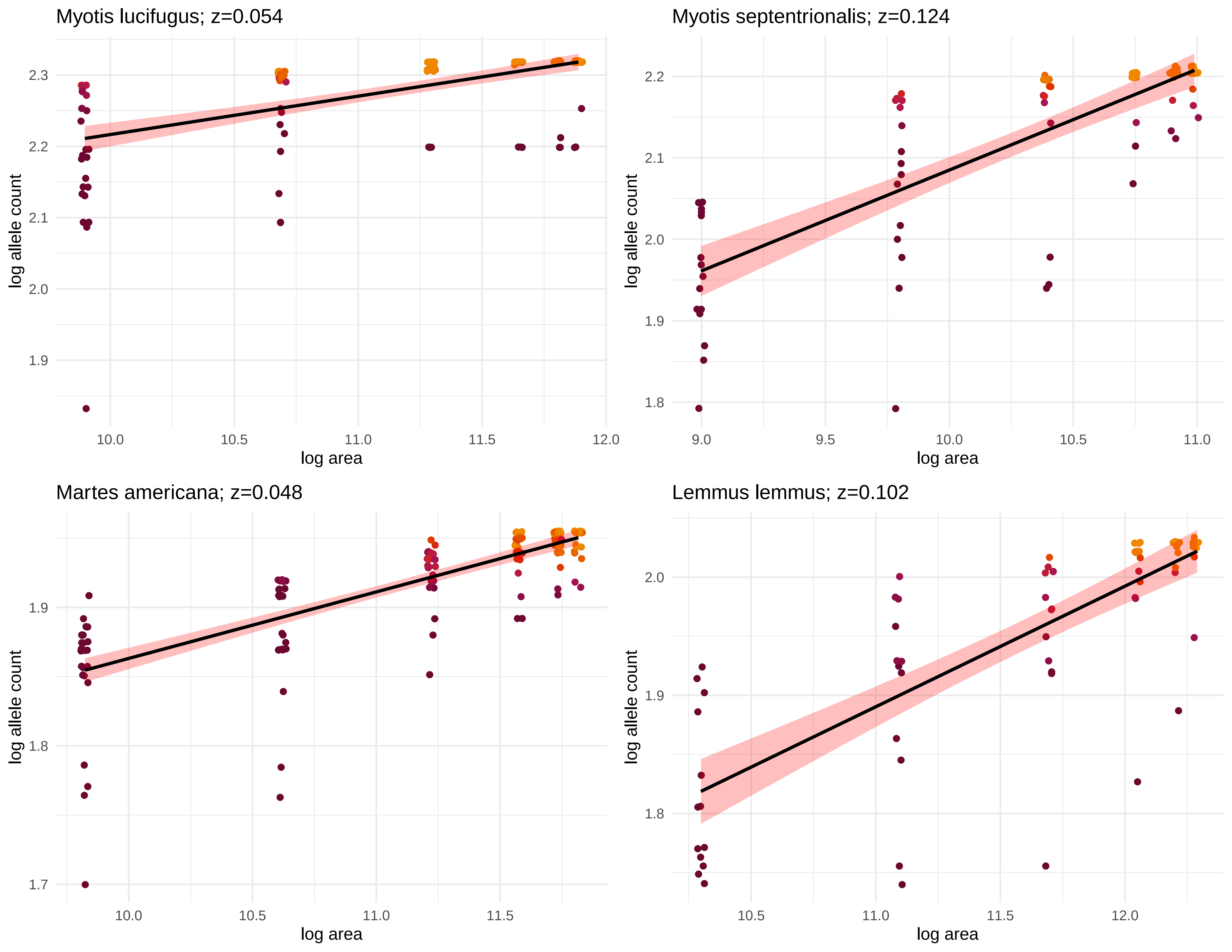
Myotis lucifugus; z=0.054
log allele count
log area
Myotis septentrionalis; z=0.124
log allele count
log area
Martes americana; z=0.048
log allele count
log area
Lemmus lemmus; z=0.102
log allele count
log area

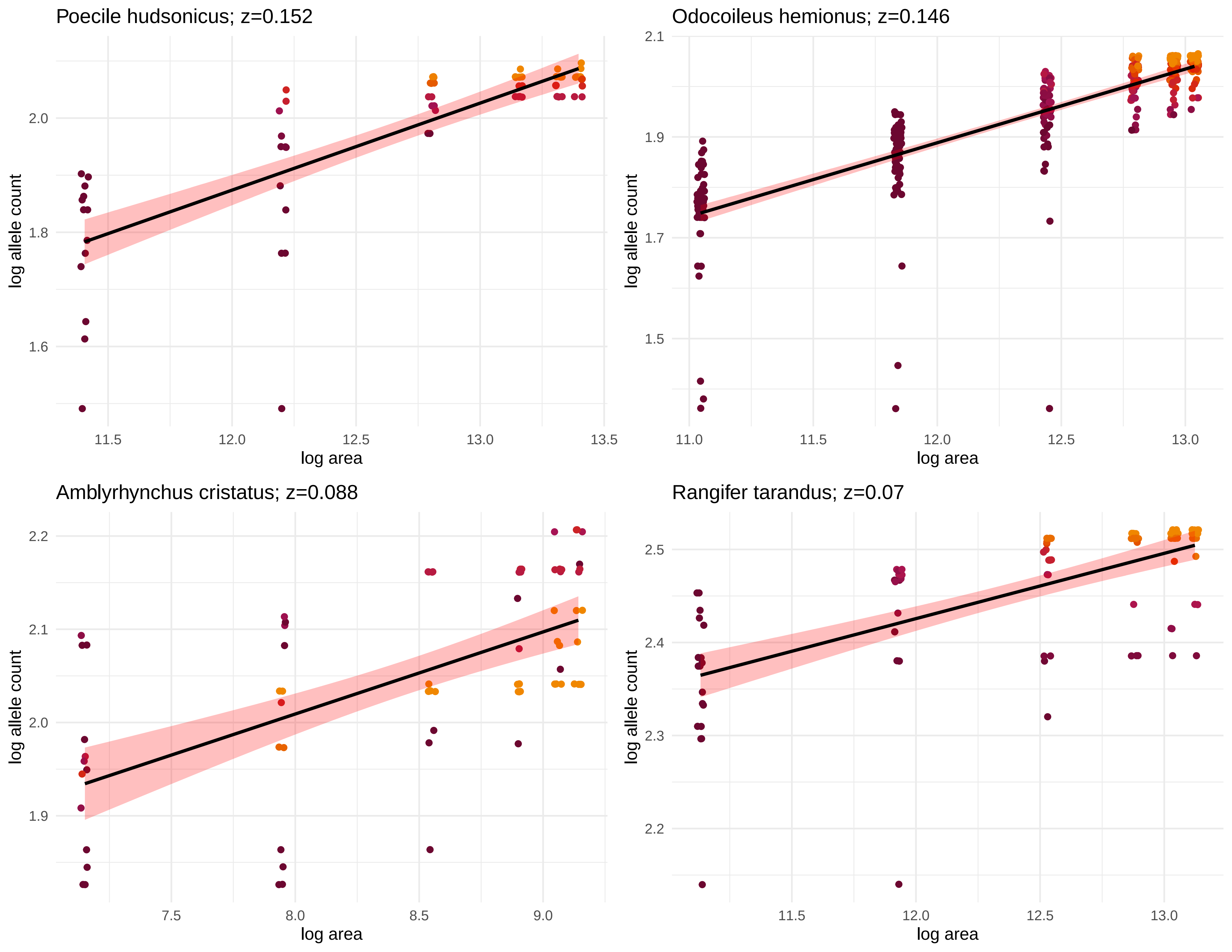
Poecile hudsonicus; z=0.152
log allele count
log area
1.6
1.8
2.0
11.5
12.0
12.5
13.0
13.5
Odocoileus hemionus; z=0.146
log allele count
log area
1.5
1.7
1.9
2.1
11.0
11.5
12.0
12.5
13.0
Amblyrhynchus cristatus; z=0.088
log allele count
log area
1.9
2.0
2.1
2.2
7.5
8.0
8.5
9.0
Rangifer tarandus; z=0.07
log allele count
log area
2.2
2.3
2.4
2.5
11.5
12.0
12.5
13.0

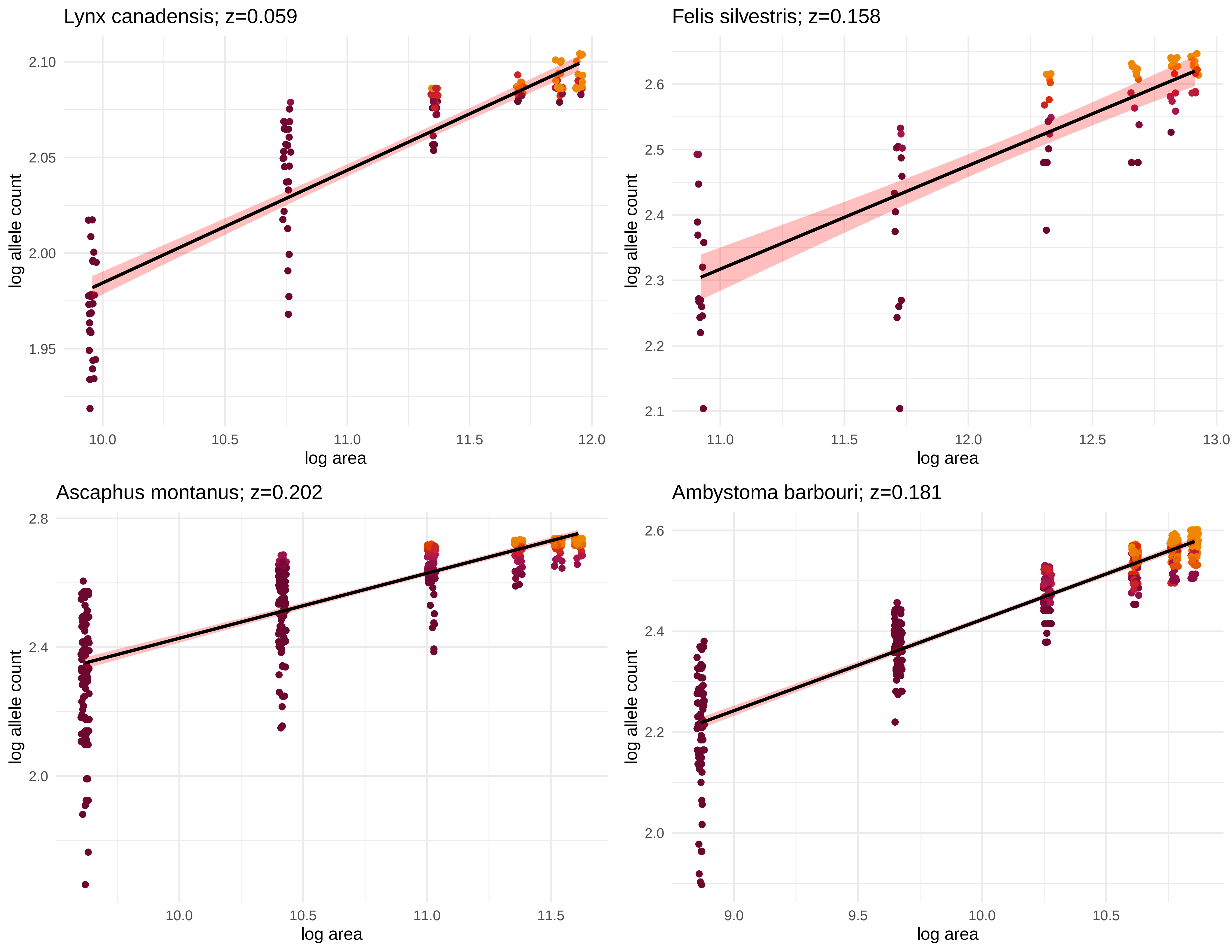

Lynx canadensis; z=0.059
Felis silvestris; z=0.158
Ascaphus montanus; z=0.202
Ambystoma barbouri; z=0.181
log allele count
log area
2.10
2.05
2.00
1.95
10.0
10.5
11.0
11.5
12.0
2.6
2.5
2.4
2.3
2.2
2.1
12.5
13.0
2.8
9.0
9.5

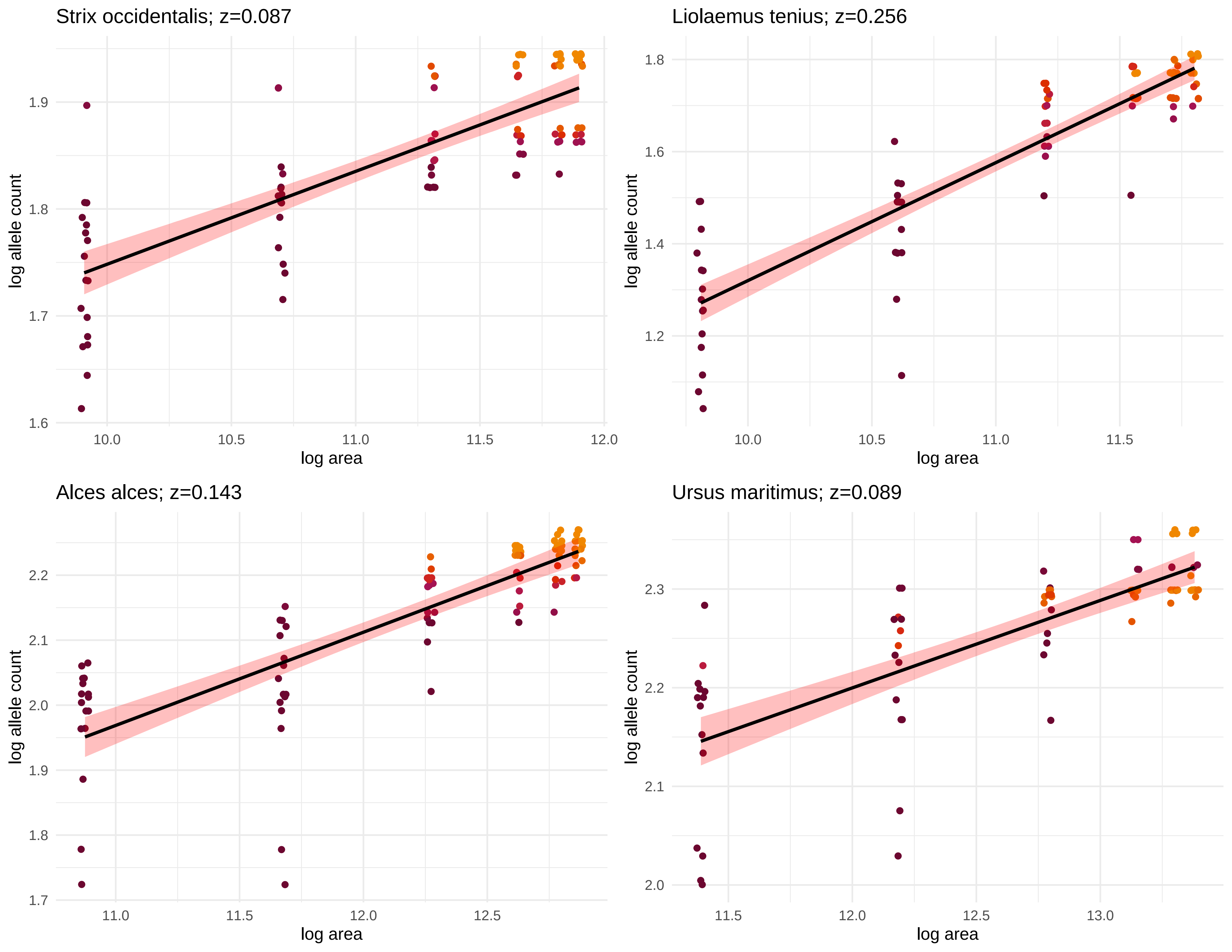
Strix occidentalis; z=0.087
Liolaemus tenius; z=0.256
Alces alces; z=0.143
Ursus maritimus; z=0.089
log allele count
log area
1.6
1.7
1.8
1.9
10.0
10.5
11.0
11.5
12.0
1.2
1.4
1.6
1.8
10.0
10.5
11.0
11.5
1.7
1.8
1.9
2.0
2.1
2.2
11.0
11.5
12.0
12.5
2.0
2.1
2.2
2.3
11.5
12.0
12.5
13.0

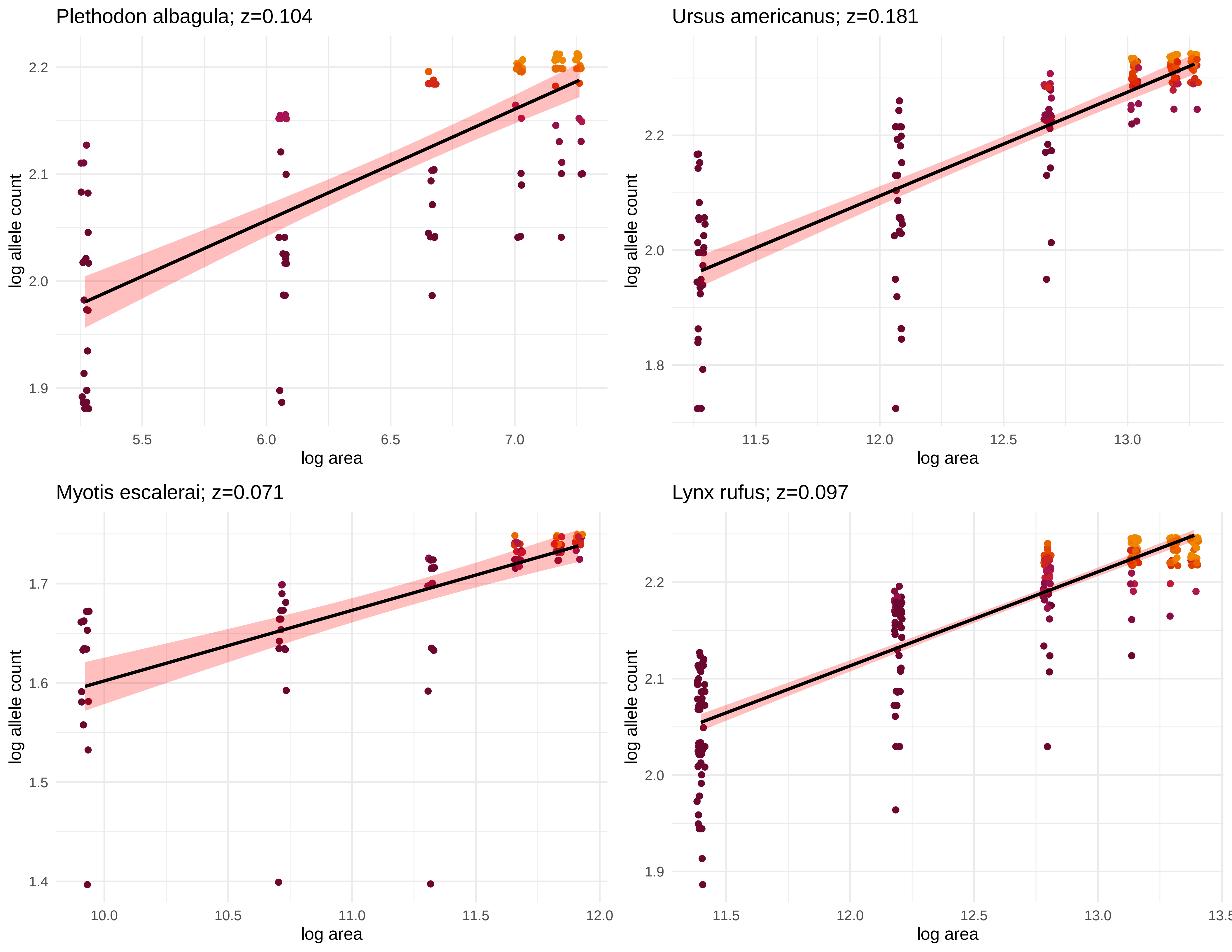
Plethodon albagula; z=0.104
log allele count
log area
Ursus americanus; z=0.181
log allele count
log area
Myotis escalerai; z=0.071
log allele count
log area
Lynx rufus; z=0.097
log allele count
log area

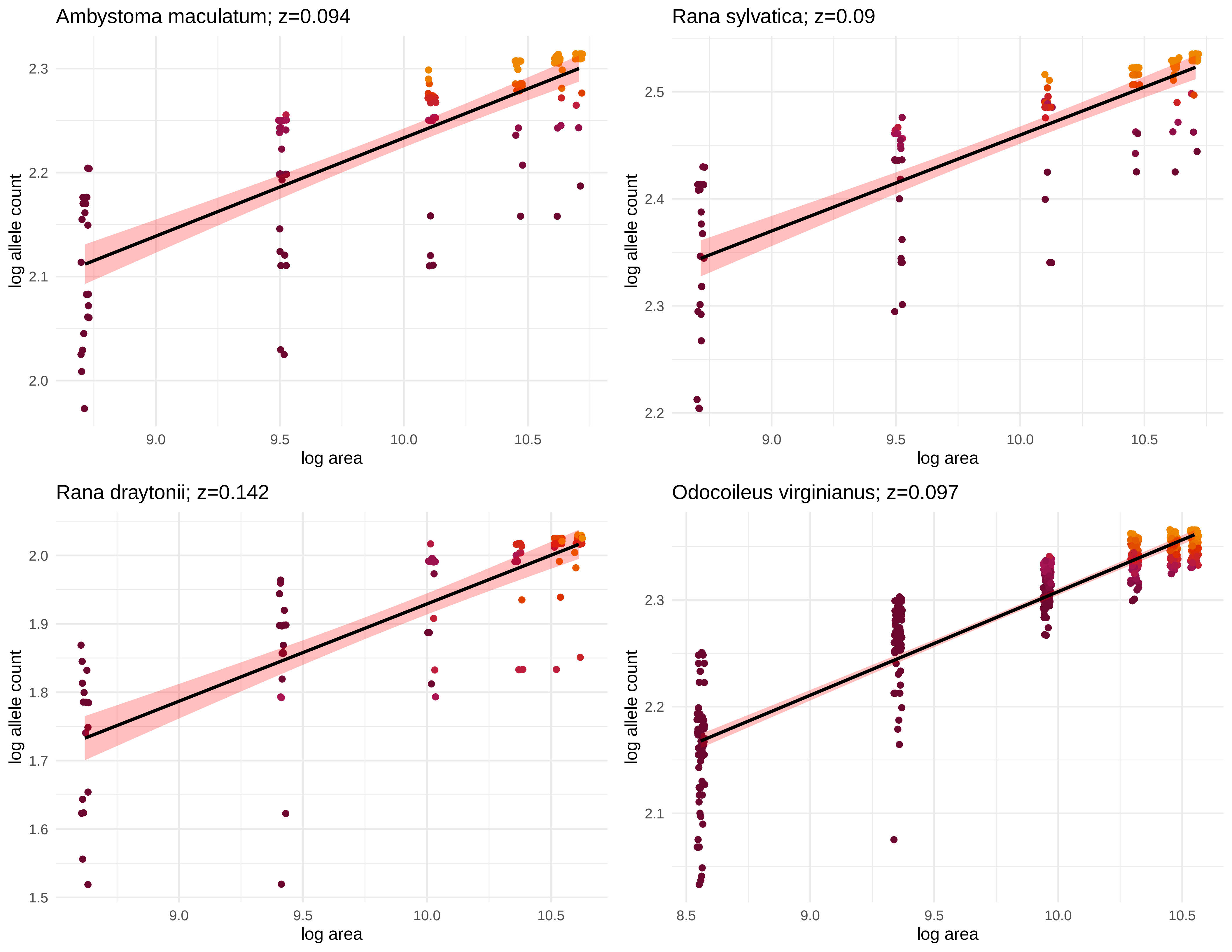

Ambystoma maculatum; z=0.094
Rana sylvatica; z=0.09
Rana draytonii; z=0.142
Odocoileus virginianus; z=0.097
log allele count
log area

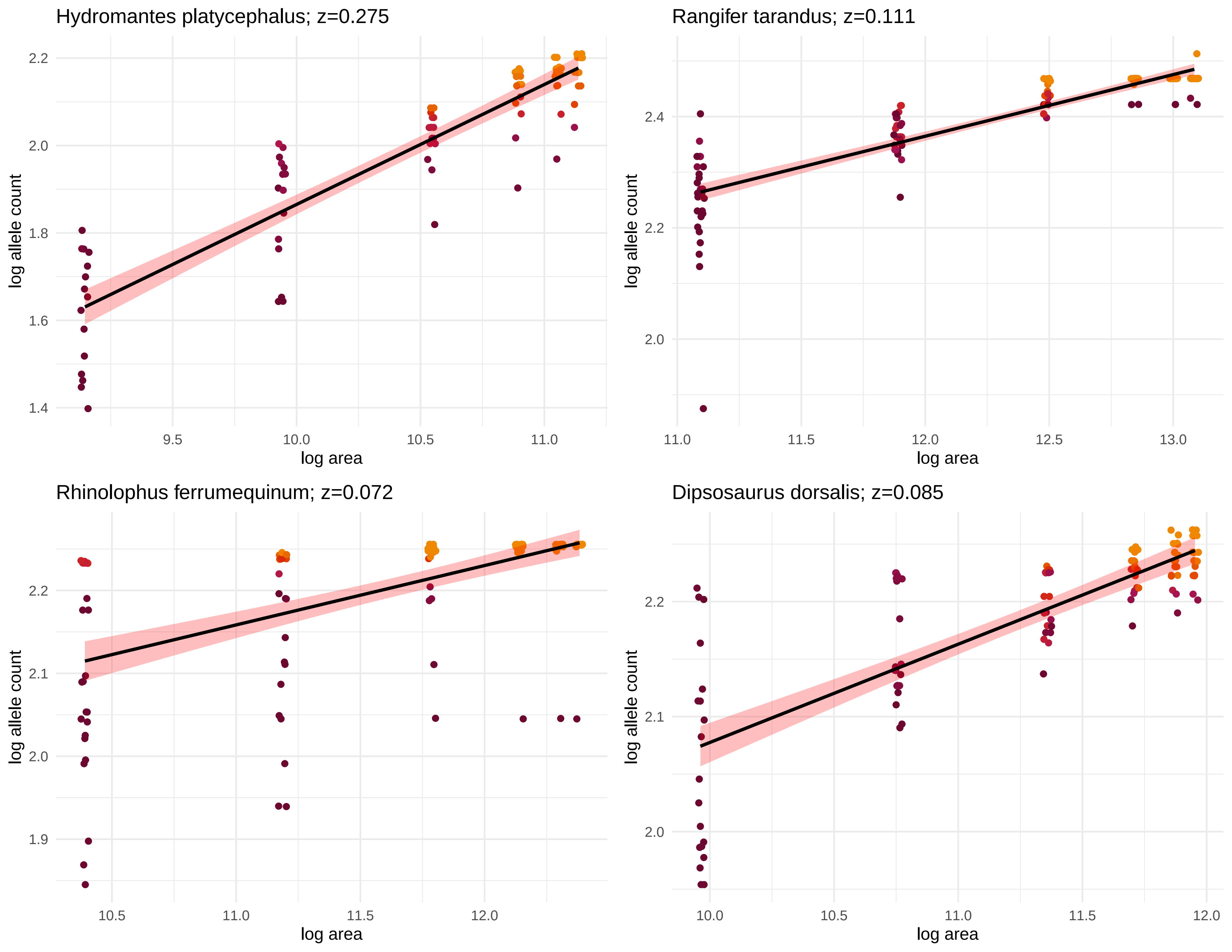

Hydromantes platycephalus; z=0.275
Rangifer tarandus; z=0.111
Rhinolophus ferrumequinum; z=0.072
Dipsosaurus dorsalis; z=0.085
log allele count
log area
1.4
1.6
1.8
2.0
2.2
9.5
10.0
10.5
11.0
11.5
12.0
12.5
13.0
1.9
2.1

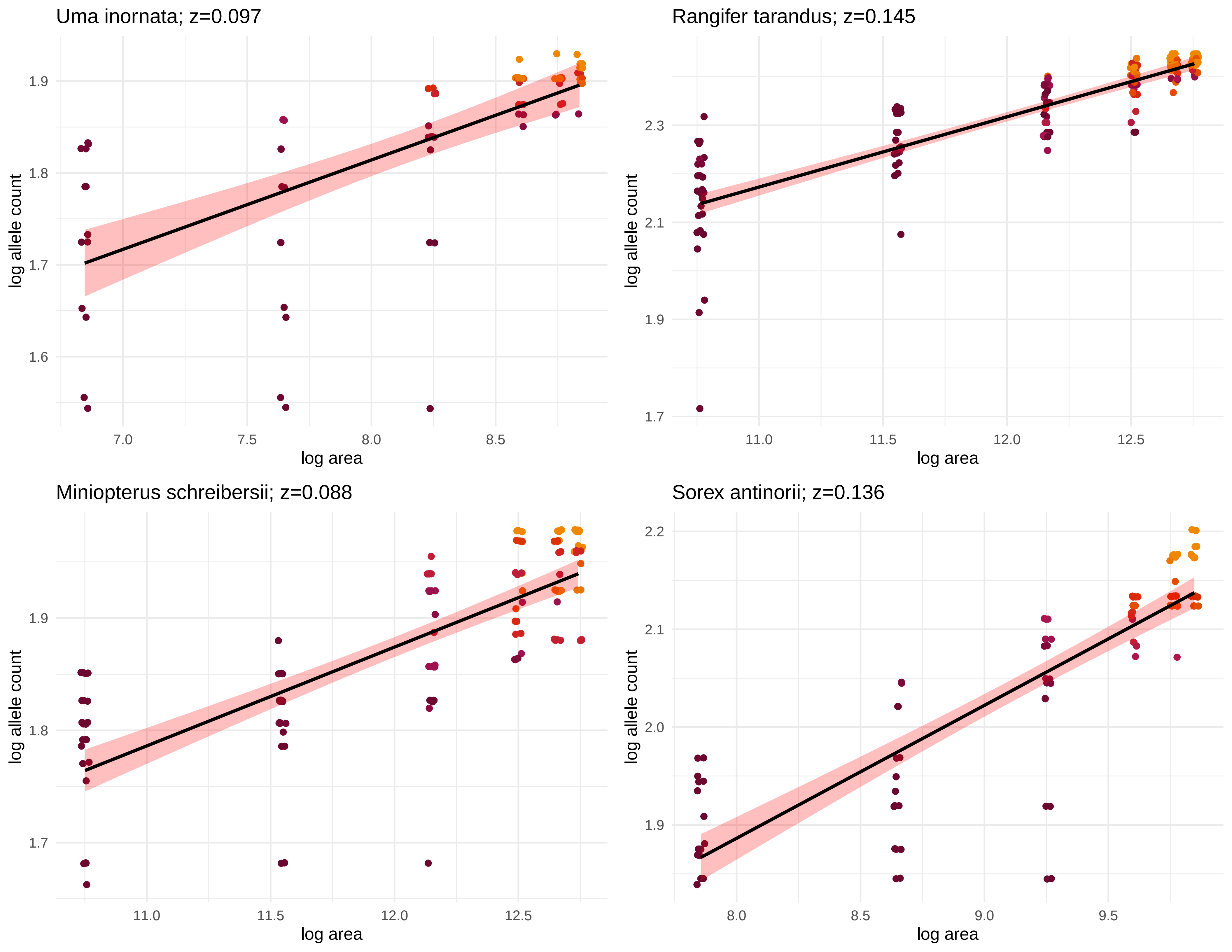

Uma inornata; z=0.097
Rangifer tarandus; z=0.145
Miniopterus schreibersii; z=0.088
Sorex antinorii; z=0.136
log allele count
log area

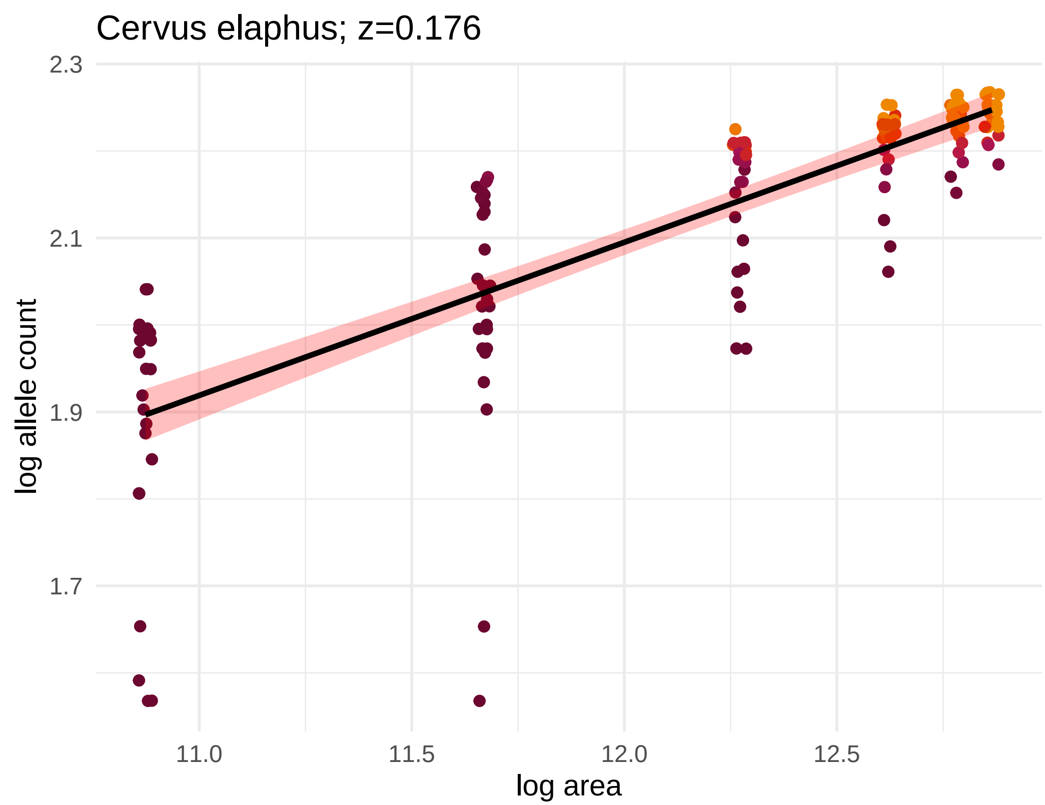
Cervus elaphus; z=0.176
2.3
2.1
1.9
1.7
log allele count
11.0
11.5
12.0
12.5
log area

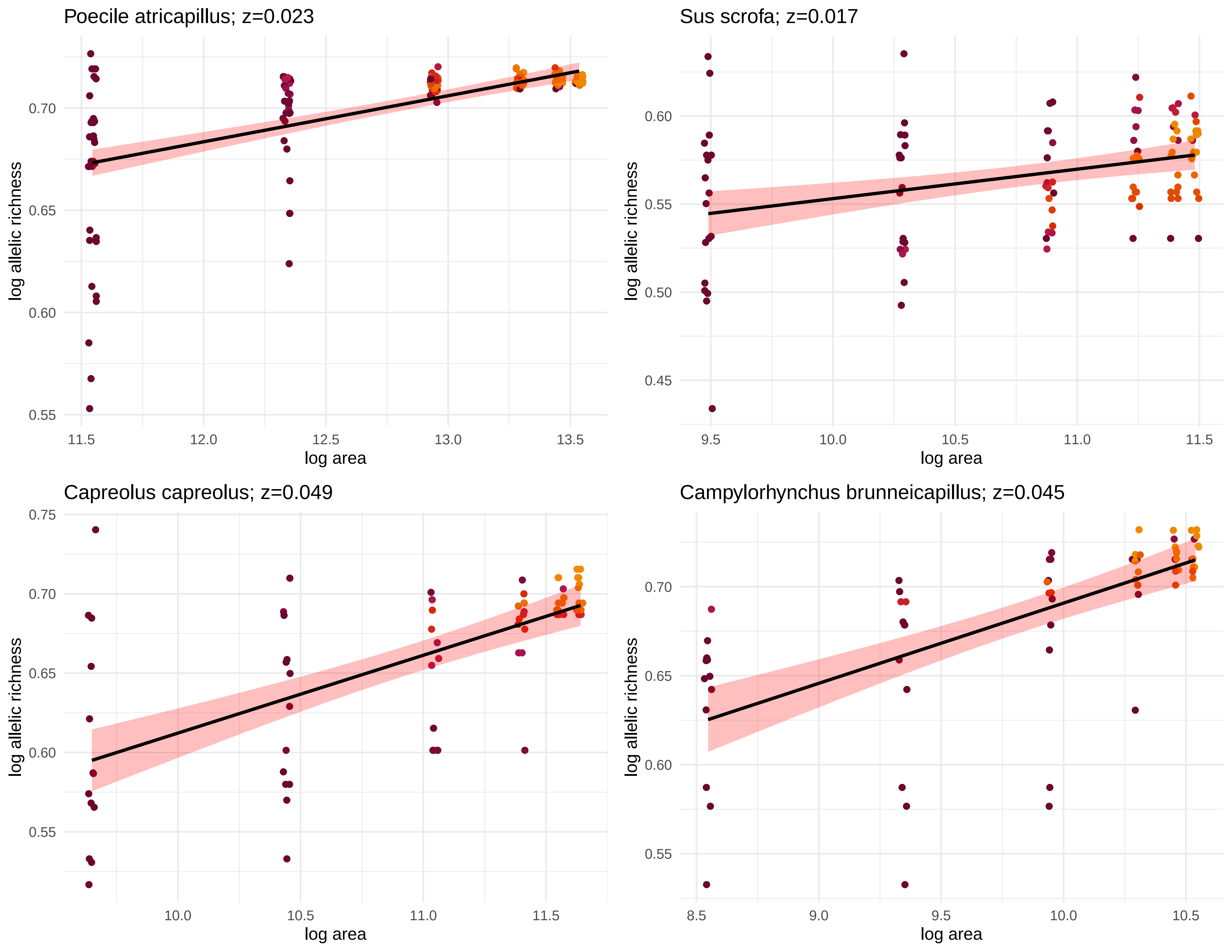
Poecile atricapillus; z=0.023
Sus scrofa; z=0.017
Capreolus capreolus; z=0.049
Campylorhynchus brunneicapillus; z=0.045
log allelic richness
log area
0.55
0.60
0.65
0.70
0.45
0.50
0.75
11.5
12.0
12.5
13.0
13.5
9.5
10.0
10.5
11.0
8.5
9.0

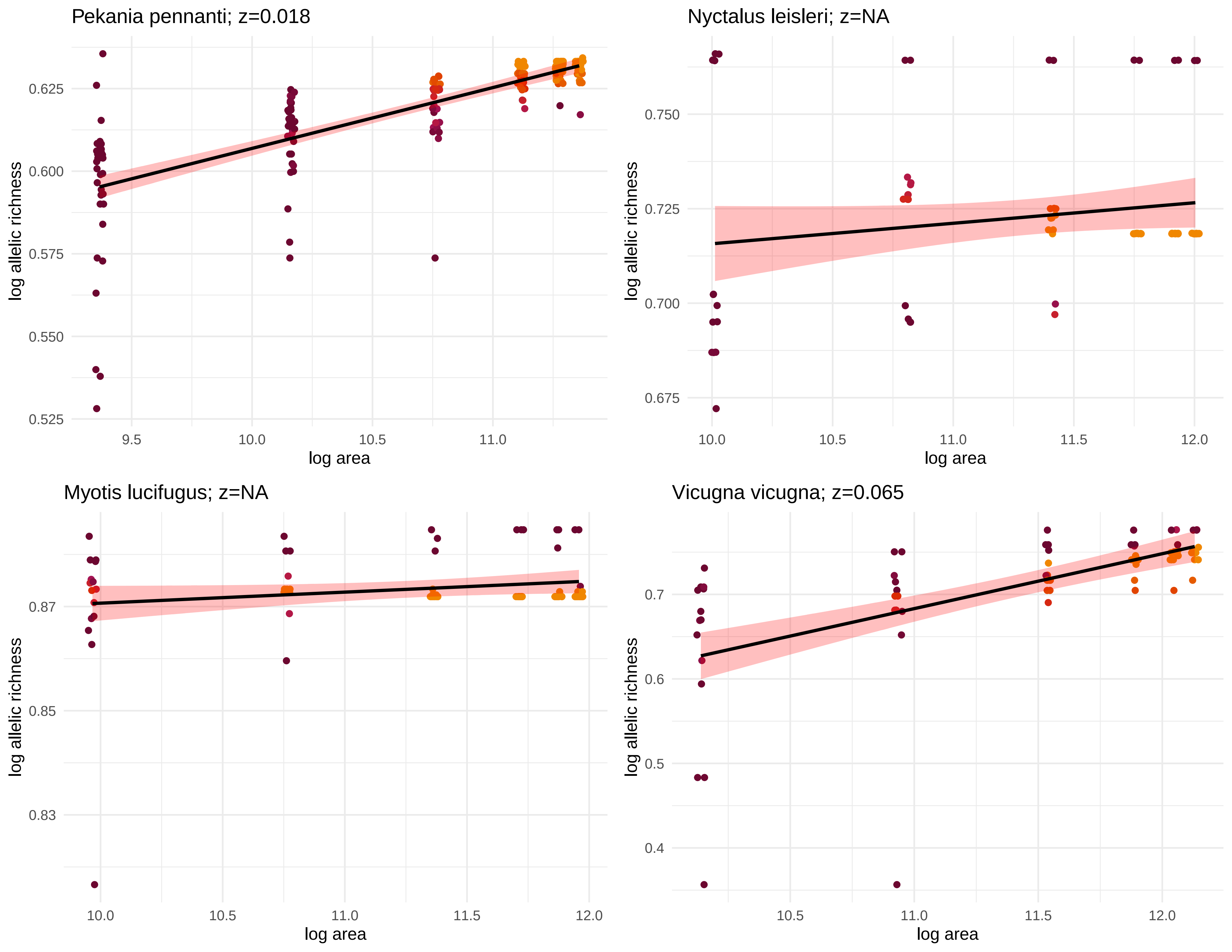
Pekania pennanti; z=0.018
Nyctalus leisleri; z=NA
Myotis lucifugus; z=NA
Vicugna vicugna; z=0.065
log allelic richness
log area
0.625
0.600
0.575
0.550
0.525
9.5
10.0
10.5
11.0
0.750
0.725
0.700
0.675
11.5
12.0
0.87
0.85
0.83
0.7
0.6
0.5
0.4

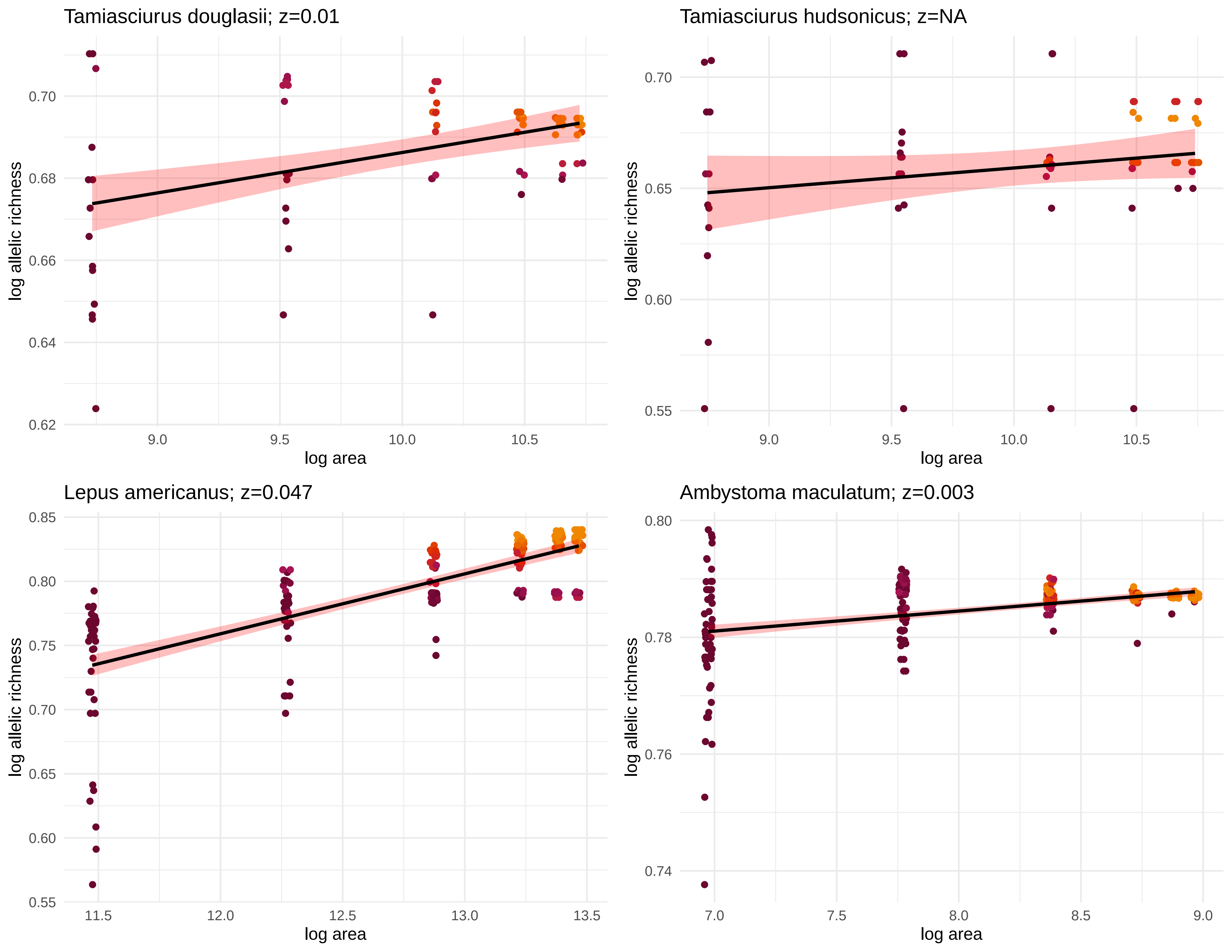
Tamiasciurus douglasii; z=0.01
Tamiasciurus hudsonicus; z=NA
Lepus americanus; z=0.047
Ambystoma maculatum; z=0.003
log allelic richness
log area
0.62
0.64
0.66
0.68
0.70
0.55
0.60
0.65
0.70
0.75
0.80
0.85
0.74
0.76
0.78
9.0
9.5
10.0
10.5
11.5
12.0
12.5
13.0
13.5
7.0
7.5
8.0
8.5

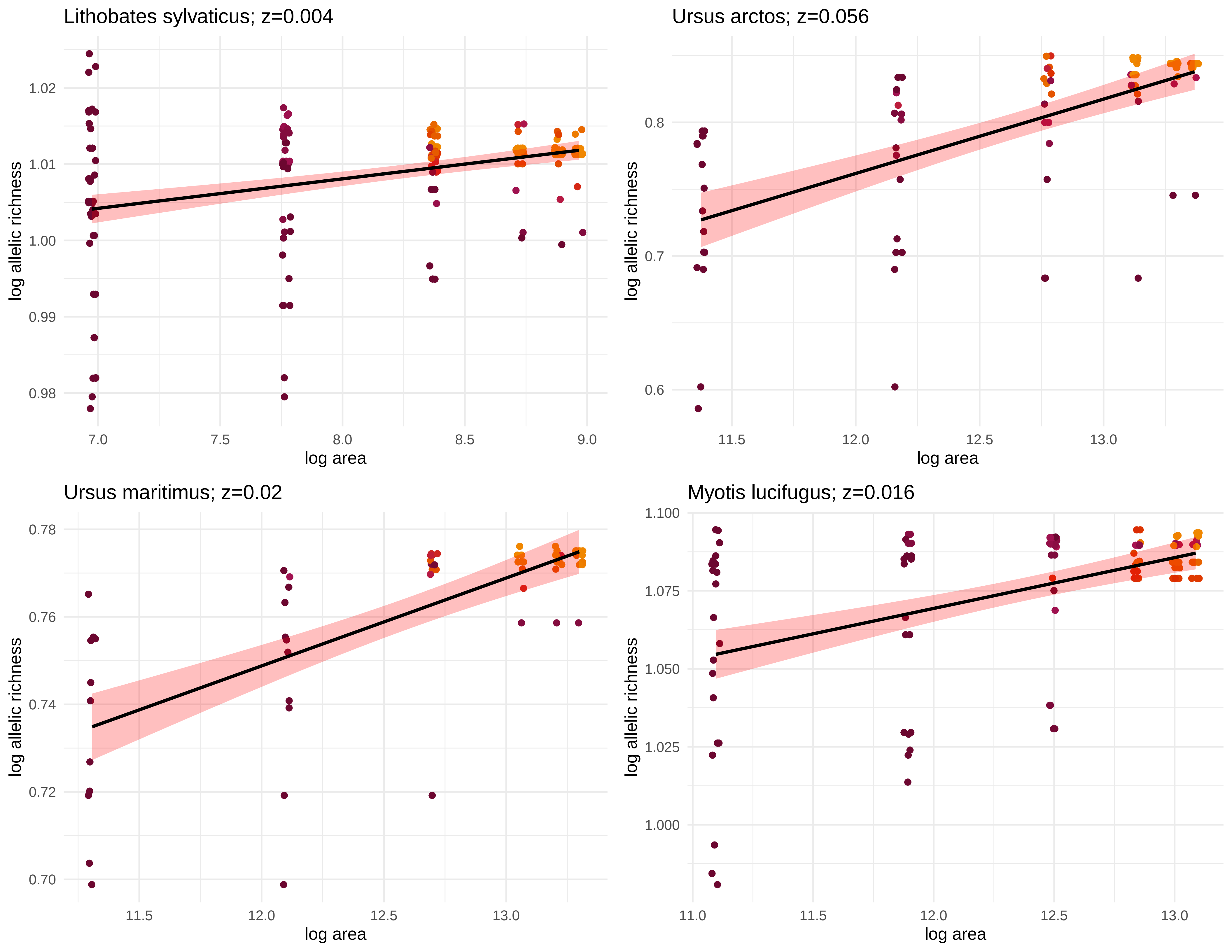
Lithobates sylvaticus; z=0.004
log allelic richness
1.02
1.01
1.00
0.99
0.98
7.0
7.5
8.0
8.5
9.0
log area
Ursus arctos; z=0.056
log allelic richness
0.8
0.7
0.6
11.5
12.0
12.5
13.0
log area
Ursus maritimus; z=0.02
log allelic richness
0.78
0.76
0.74
0.72
0.70
11.5
12.0
12.5
13.0
log area
Myotis lucifugus; z=0.016
log allelic richness
1.100
1.075
1.050
1.025
1.000
11.0
11.5
12.0
12.5
13.0
log area

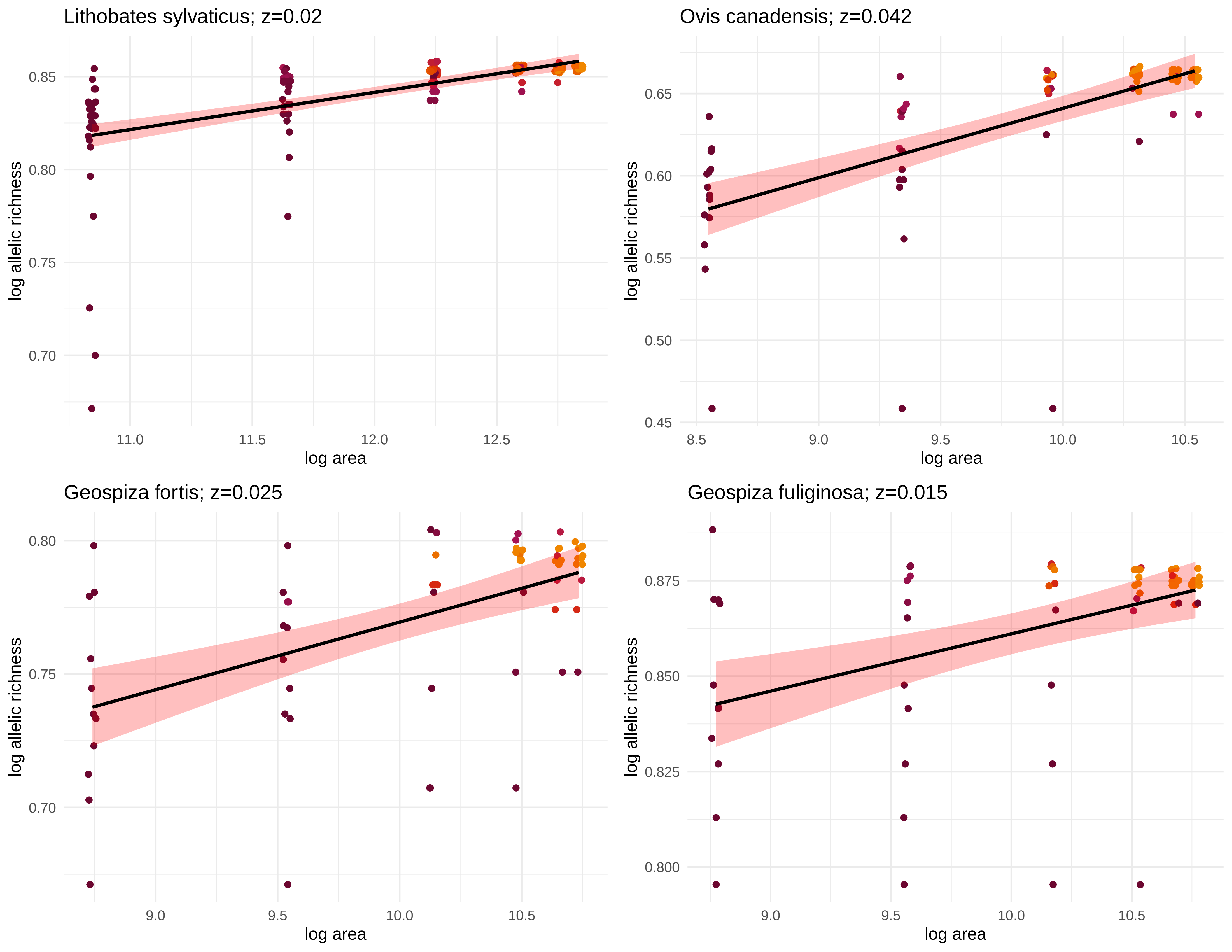
Lithobates sylvaticus; z=0.02
Ovis canadensis; z=0.042
Geospiza fortis; z=0.025
Geospiza fuliginosa; z=0.015
log allelic richness
log area
0.85
0.80
0.75
0.70
11.0
11.5
12.0
12.5
0.65
0.60
0.55
0.50
0.45
8.5
9.0
9.5
10.0
10.5
0.875
0.850
0.825
0.800

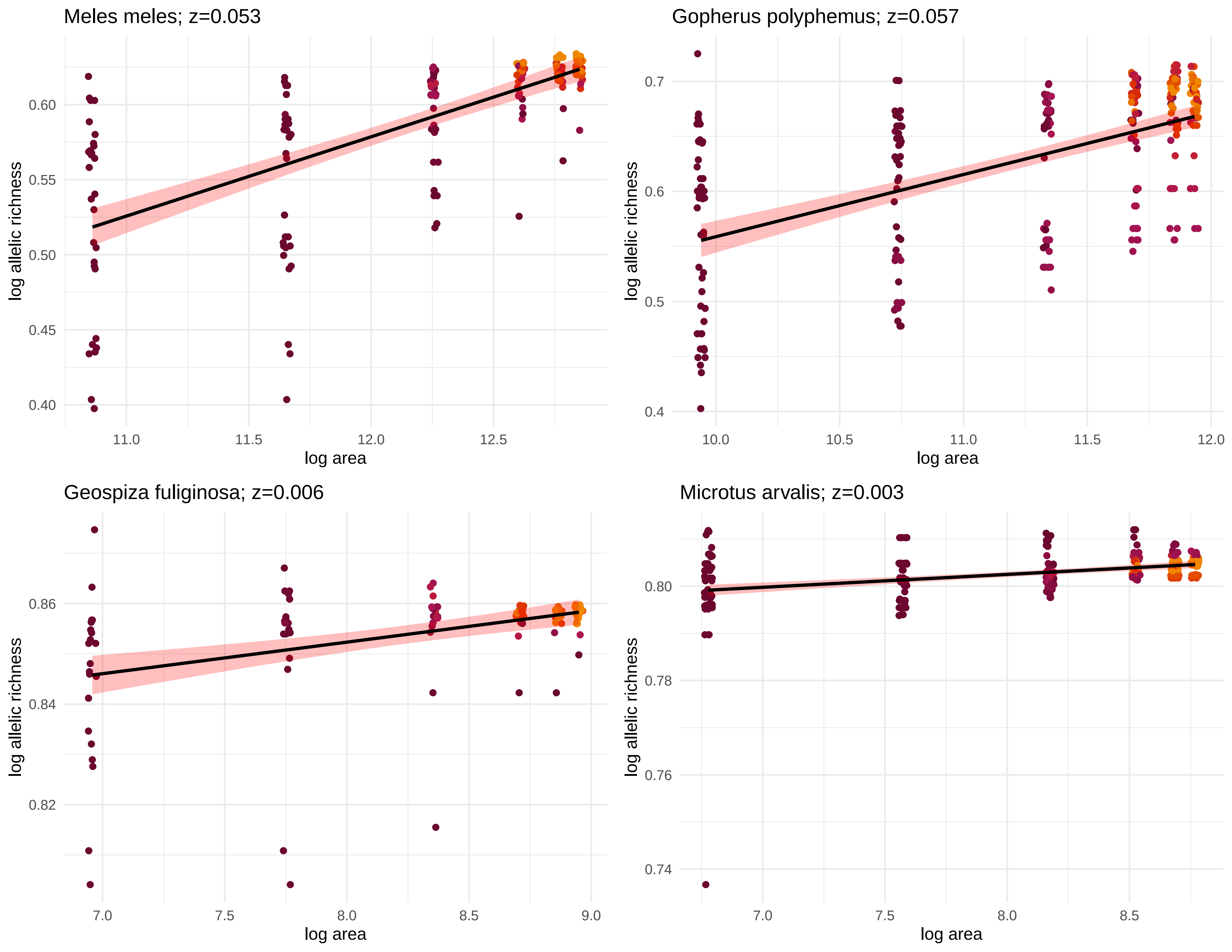
Meles meles; z=0.053
Gopherus polyphemus; z=0.057
Geospiza fuliginosa; z=0.006
Microtus arvalis; z=0.003
log allelic richness
log area
0.40
0.45
0.50
0.55
0.60
11.0
11.5
12.0
12.5
0.4
0.5
0.6
0.7
10.0
10.5
11.0
11.5
12.0
0.82
0.84
0.86
7.0
7.5
8.0
8.5
9.0
0.74
0.76
0.78
0.80
7.0
7.5
8.0
8.5

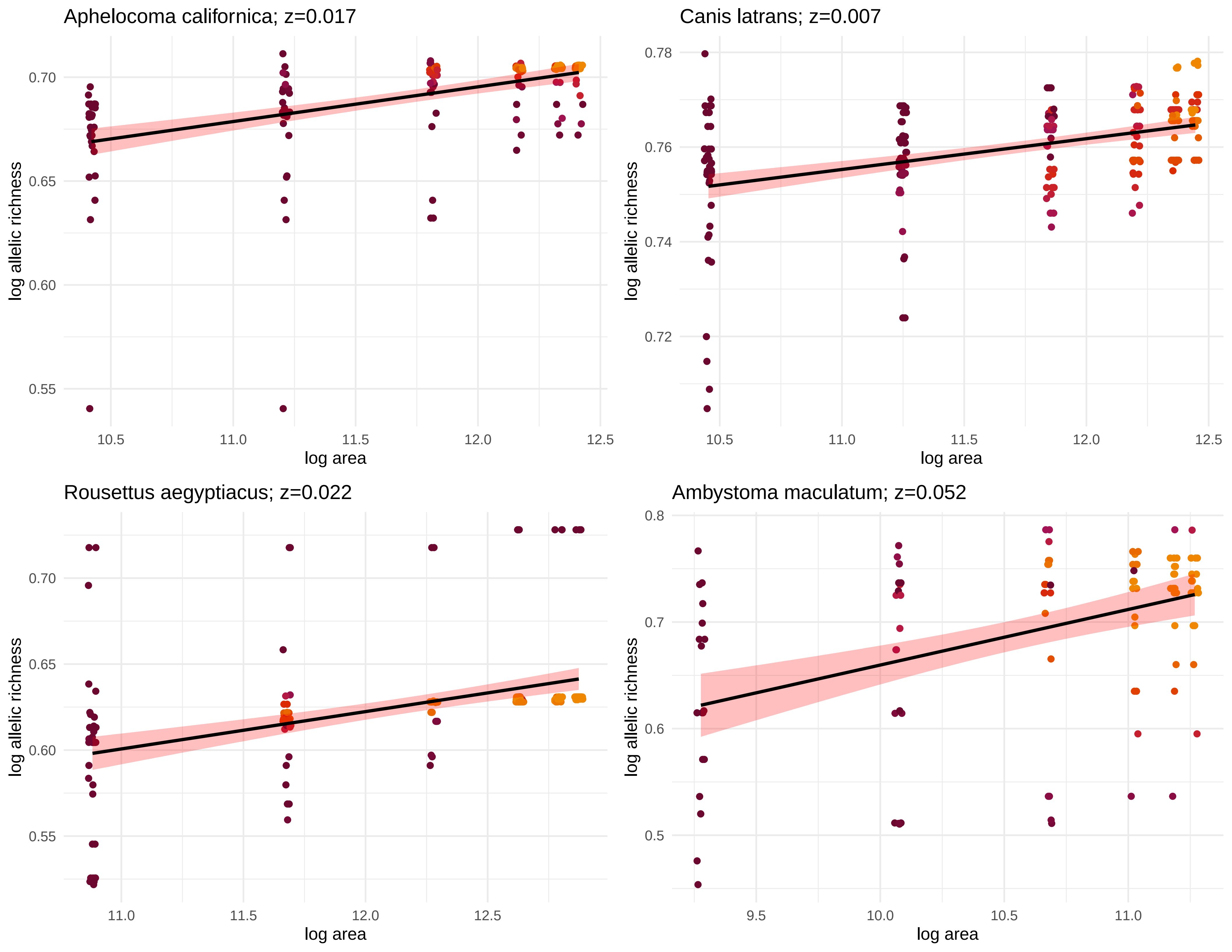

Aphelocoma californica; z=0.017
Canis latrans; z=0.007
Rousettus aegyptiacus; z=0.022
Ambystoma maculatum; z=0.052
log allelic richness
log area
0.70
0.65
0.60
0.55
10.5
11.0
11.5
12.0
12.5
0.78
0.76
0.74
0.72
0.8
0.7
0.6
0.5
9.5
10.0

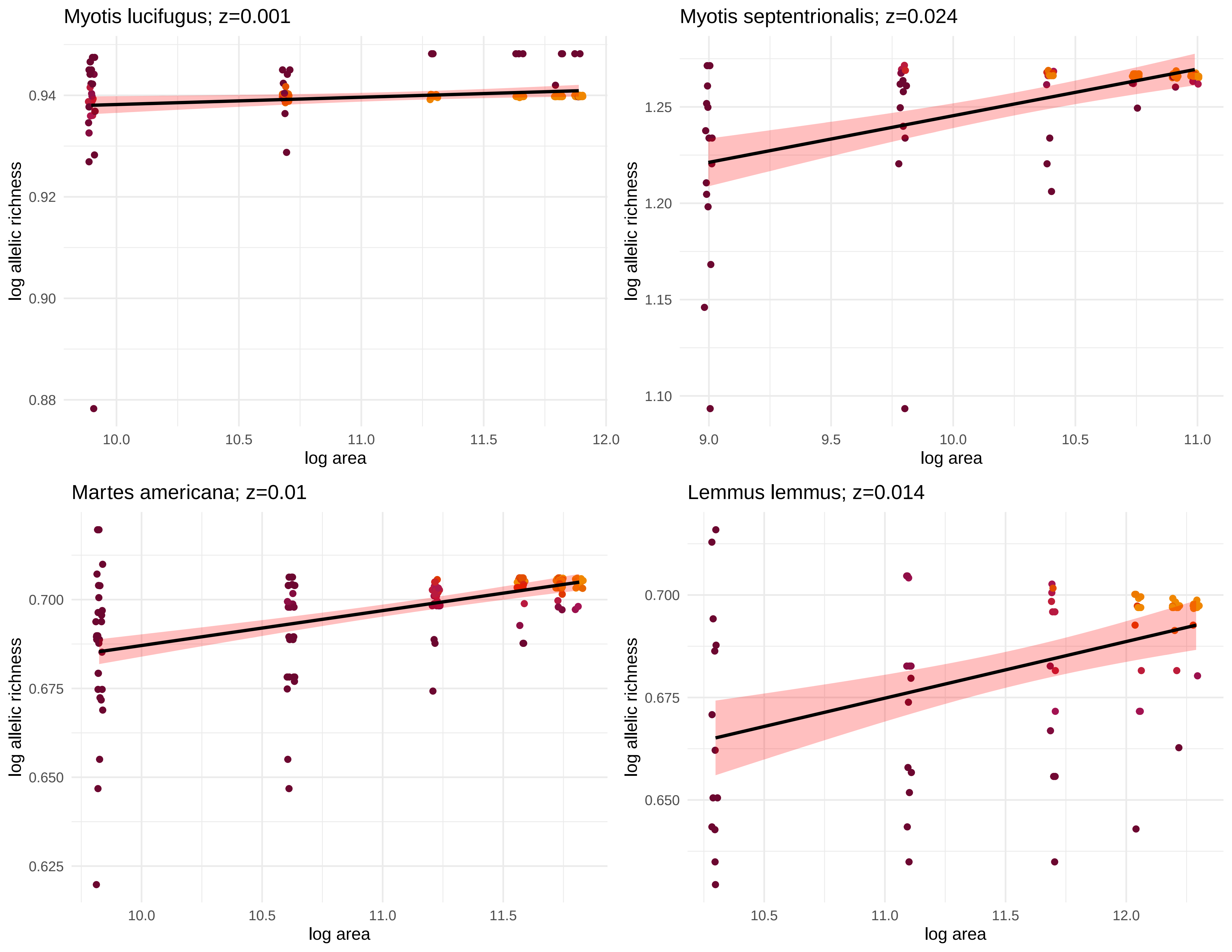
Myotis lucifugus; z=0.001
Myotis septentrionalis; z=0.024
Martes americana; z=0.01
Lemmus lemmus; z=0.014
log allelic richness
log area
0.88
0.90
0.92
0.94
10.0
10.5
11.0
11.5
12.0
1.10
1.15
1.20
1.25
9.0
9.5
0.625
0.650
0.675
0.700

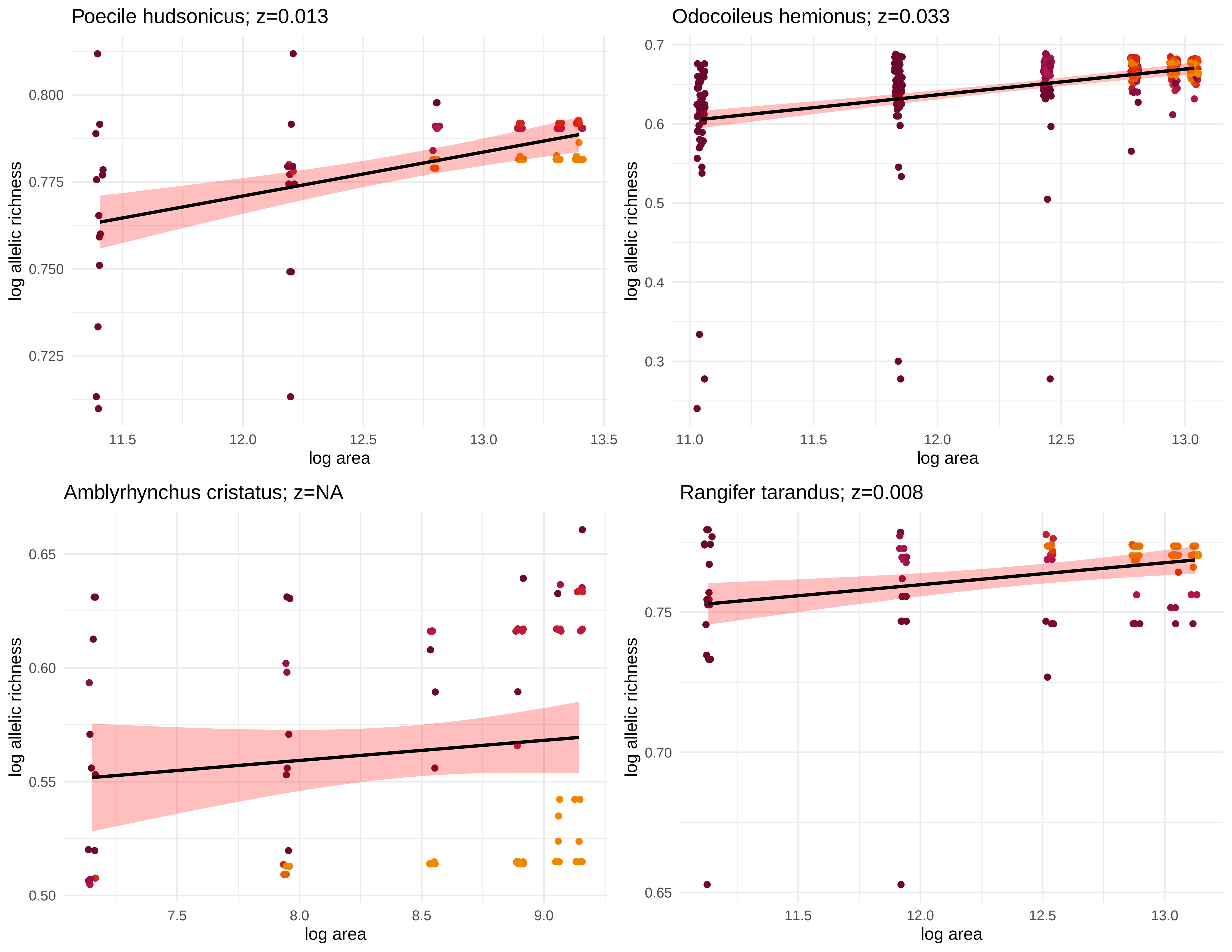

Poecile hudsonicus; z=0.013
Odocoileus hemionus; z=0.033
Amblyrhynchus cristatus; z=NA
Rangifer tarandus; z=0.008
log allelic richness
log area
0.800
0.775
0.750
0.725
11.5
12.0
12.5
13.0
13.5
0.7
0.6
0.5
0.4
0.3
11.0
0.65
0.60
0.55
0.50
7.5
8.0
8.5
9.0
0.75
0.70

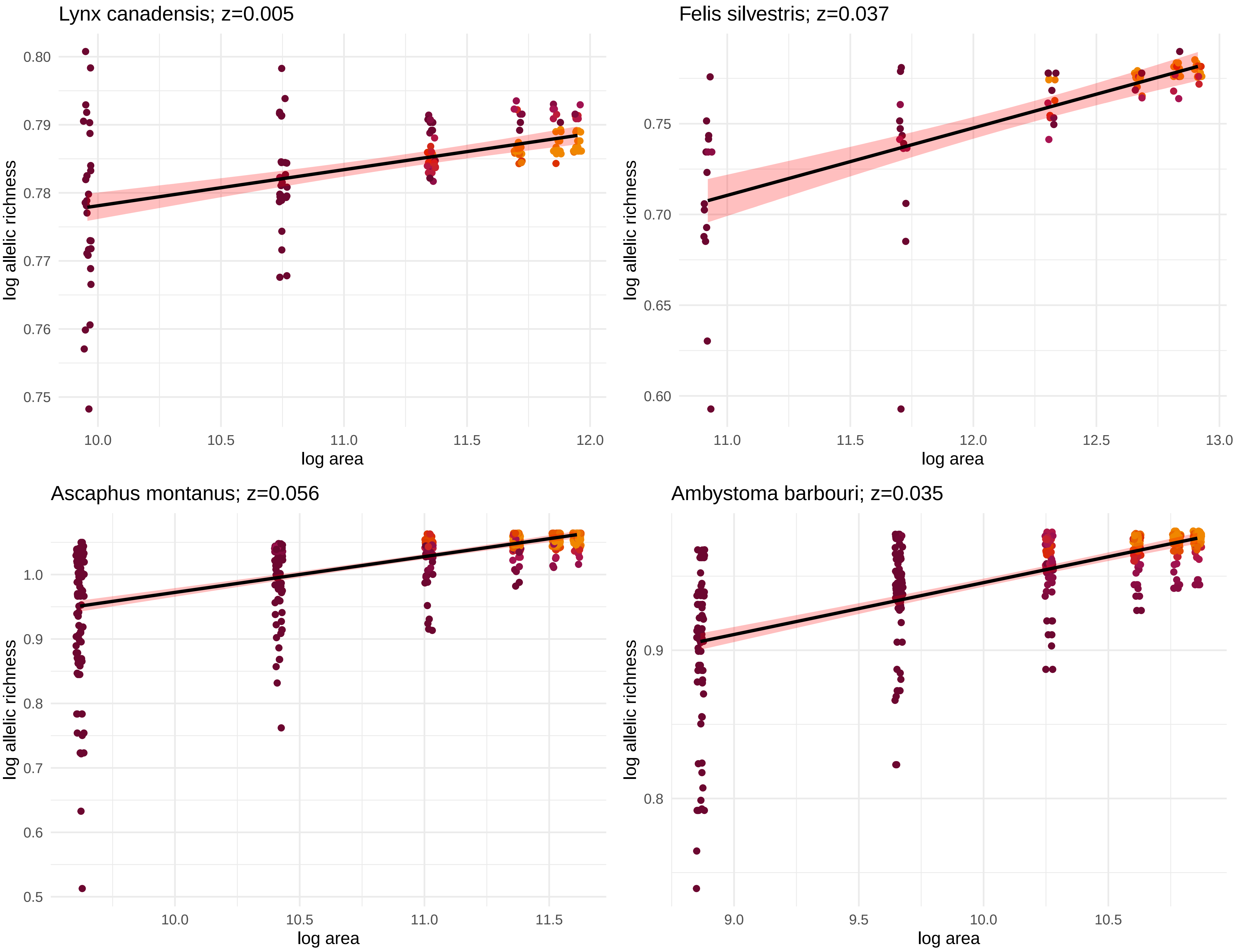
Lynx canadensis; z=0.005
0.80
0.79
0.78
0.77
0.76
0.75
log allelic richness
10.0
10.5
11.0
11.5
12.0
log area
Felis silvestris; z=0.037
0.75
0.70
0.65
0.60
log allelic richness
11.0
11.5
12.0
12.5
13.0
log area
Ascaphus montanus; z=0.056
1.0
0.9
0.8
0.7
0.6
0.5
log allelic richness
10.0
10.5
11.0
11.5
log area
Ambystoma barbouri; z=0.035
0.9
0.8
log allelic richness
9.0
9.5
10.0
10.5
log area

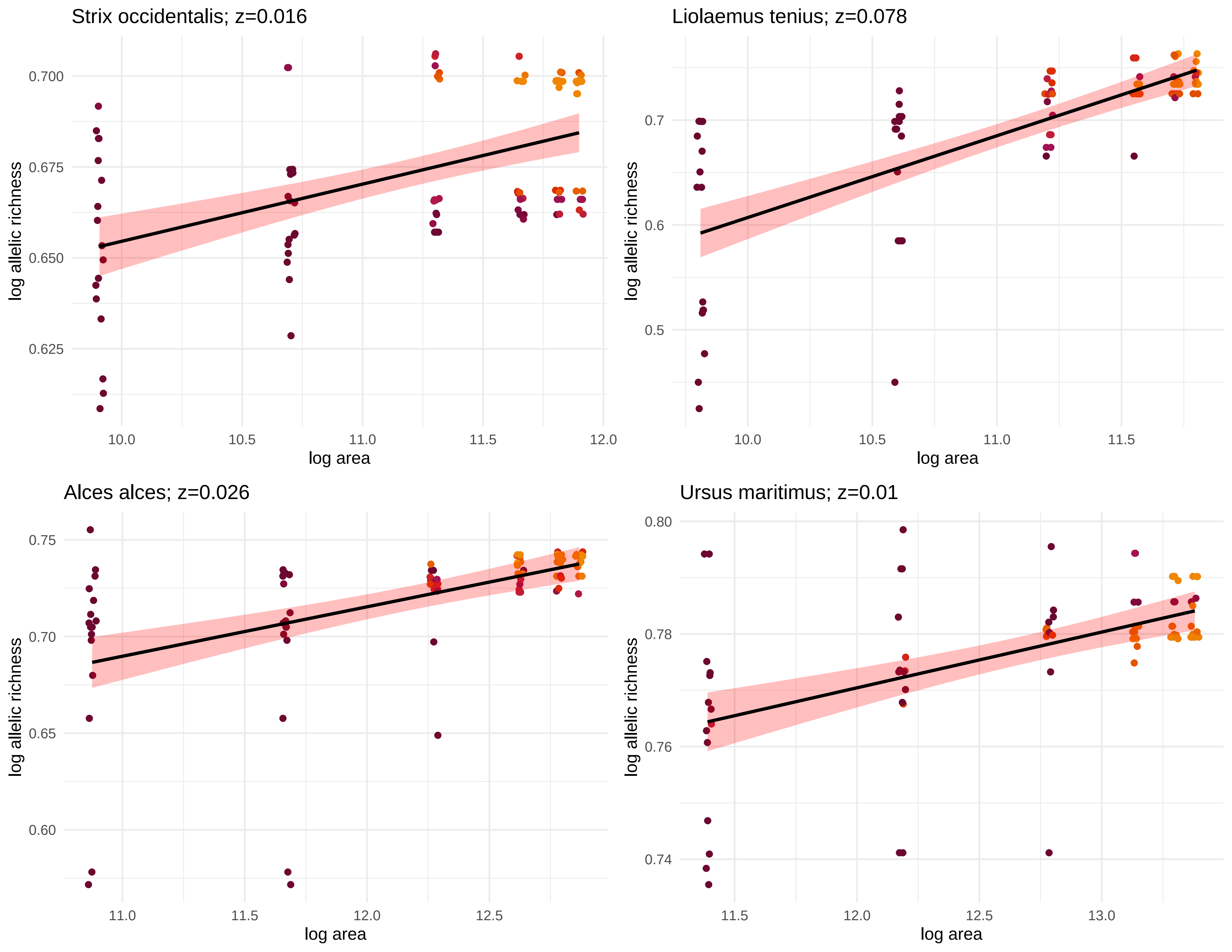
Strix occidentalis; z=0.016
Liolaemus tenius; z=0.078
Alces alces; z=0.026
Ursus maritimus; z=0.01
log allelic richness
log area
0.700
0.675
0.650
0.625
10.0
10.5
11.0
11.5
12.0
0.7
0.6
0.5
0.75
0.70
0.65
0.60
12.5
0.80
0.78
0.76
0.74
13.0

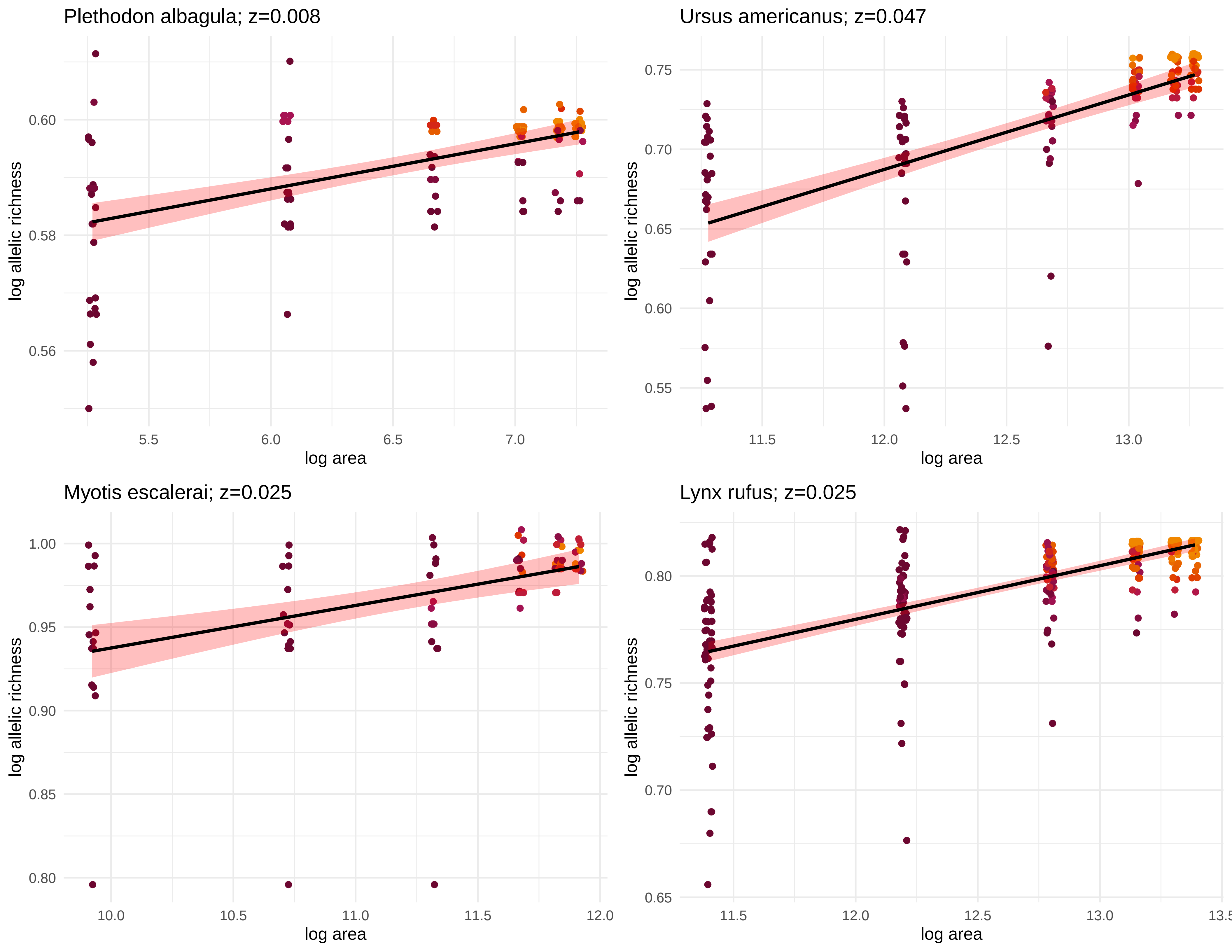

Plethodon albagula; z=0.008
0.60
0.58
0.56
log allelic richness
5.5
6.0
6.5
7.0
log area
Ursus americanus; z=0.047
0.75
0.70
0.65
0.60
0.55
log allelic richness
11.5
12.0
12.5
13.0
log area
Myotis escalerai; z=0.025
1.00
0.95
0.90
0.85
0.80
log allelic richness
10.0
10.5
11.0
11.5
12.0
log area
Lynx rufus; z=0.025
0.80
0.75
0.70
0.65
log allelic richness
11.5
12.0
12.5
13.0
log area

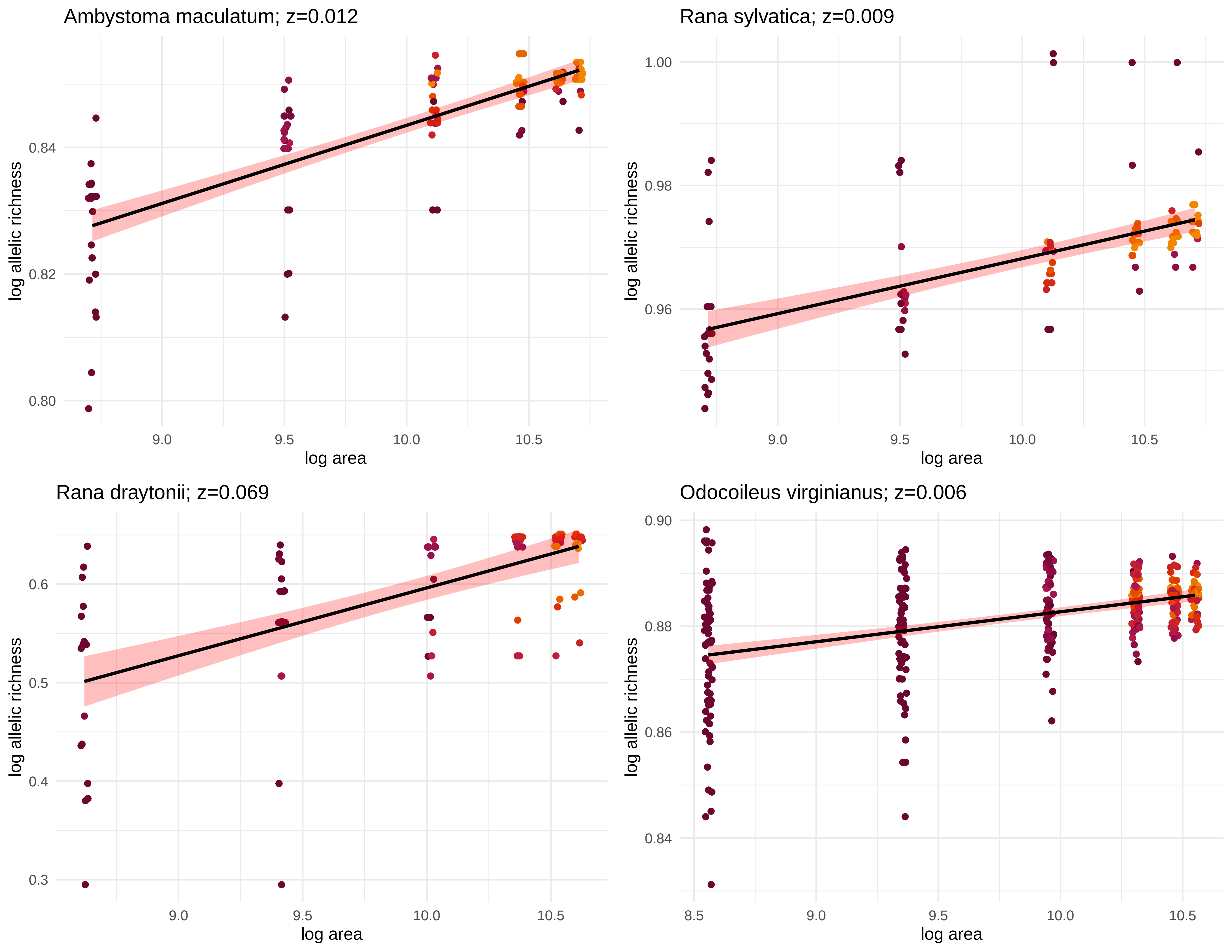
Ambystoma maculatum; z=0.012
Rana sylvatica; z=0.009
Rana draytonii; z=0.069
Odocoileus virginianus; z=0.006
log allelic richness
log area
0.80
0.82
0.84
0.96
0.98
1.00
0.3
0.4
0.5
0.6
0.84
0.86
0.88
0.90
8.5
9.0
9.5
10.0
10.5

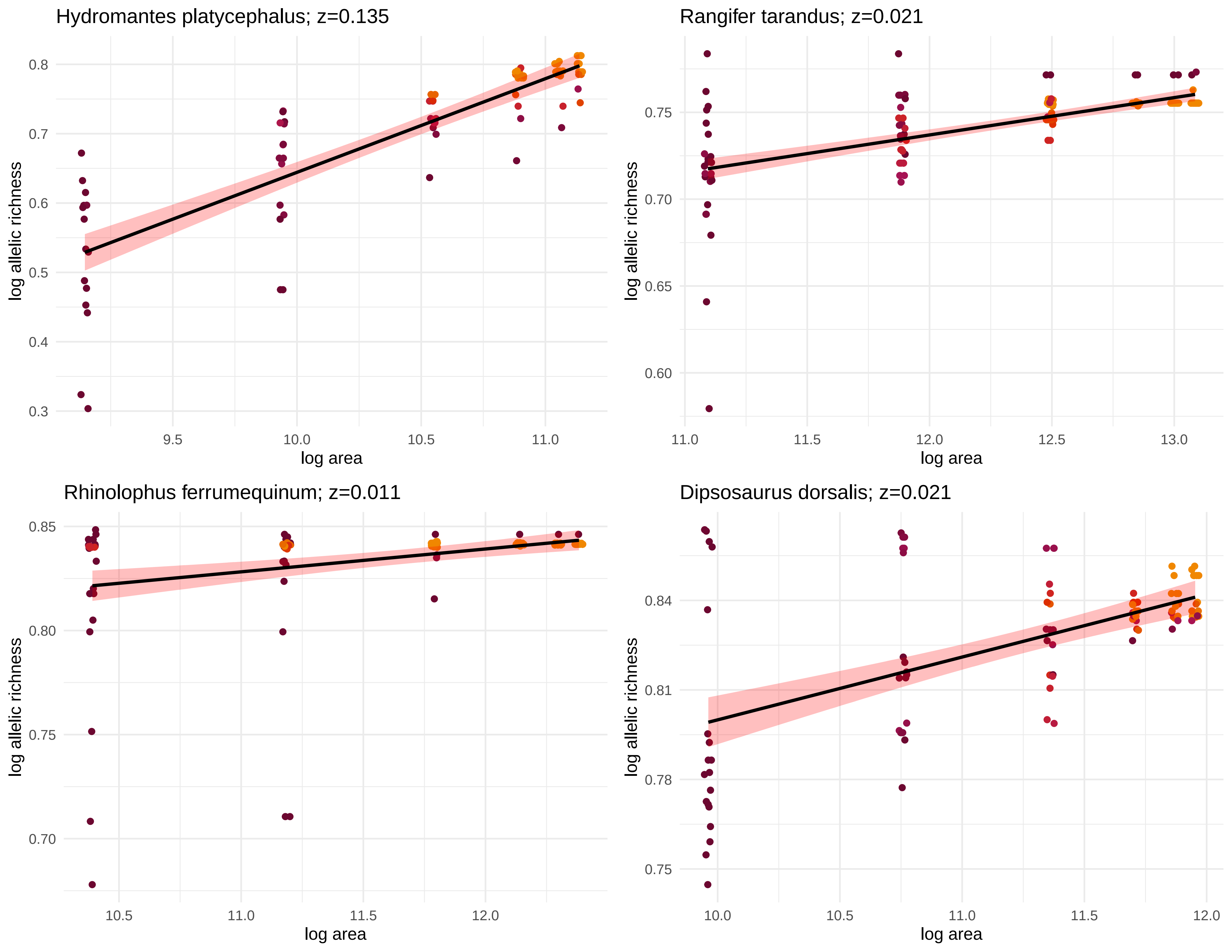
Hydromantes platycephalus; z=0.135
log allelic richness
0.3
0.4
0.5
0.6
0.7
0.8
9.5
10.0
10.5
11.0
log area
Rangifer tarandus; z=0.021
log allelic richness
0.60
0.65
0.70
0.75
11.0
11.5
12.0
12.5
13.0
log area
Rhinolophus ferrumequinum; z=0.011
log allelic richness
0.70
0.75
0.80
0.85
10.5
11.0
11.5
12.0
log area
Dipsosaurus dorsalis; z=0.021
log allelic richness
0.75
0.78
0.81
0.84
10.0
10.5
11.0
11.5
12.0
log area

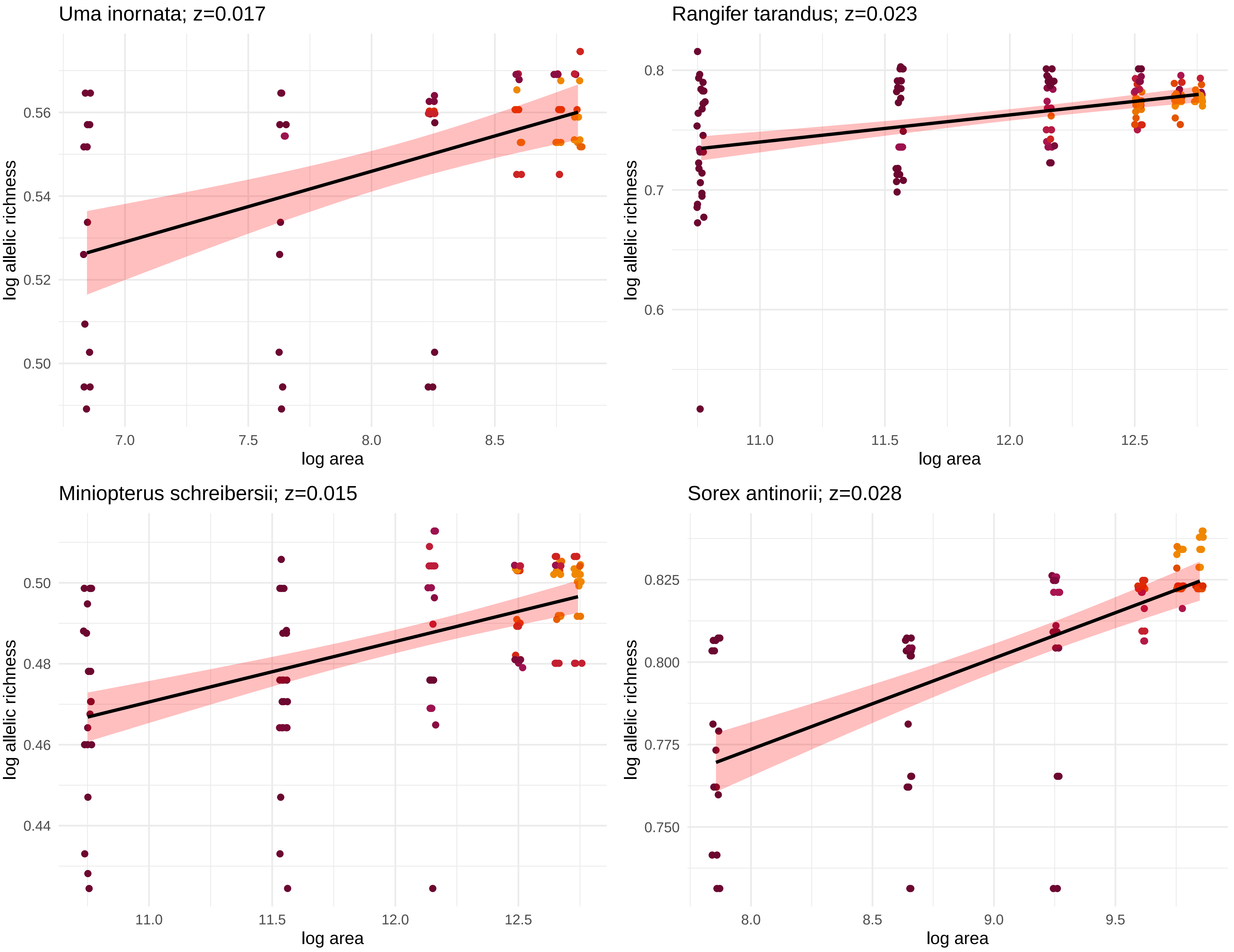
Uma inornata; z=0.017
log allelic richness
0.50
0.52
0.54
0.56
7.0
7.5
8.0
8.5
log area
Rangifer tarandus; z=0.023
log allelic richness
0.6
0.7
0.8
11.0
11.5
12.0
12.5
log area
Miniopterus schreibersii; z=0.015
log allelic richness
0.44
0.46
0.48
0.50
11.0
11.5
12.0
12.5
log area
Sorex antinorii; z=0.028
log allelic richness
0.750
0.775
0.800
0.825
8.0
8.5
9.0
9.5
log area

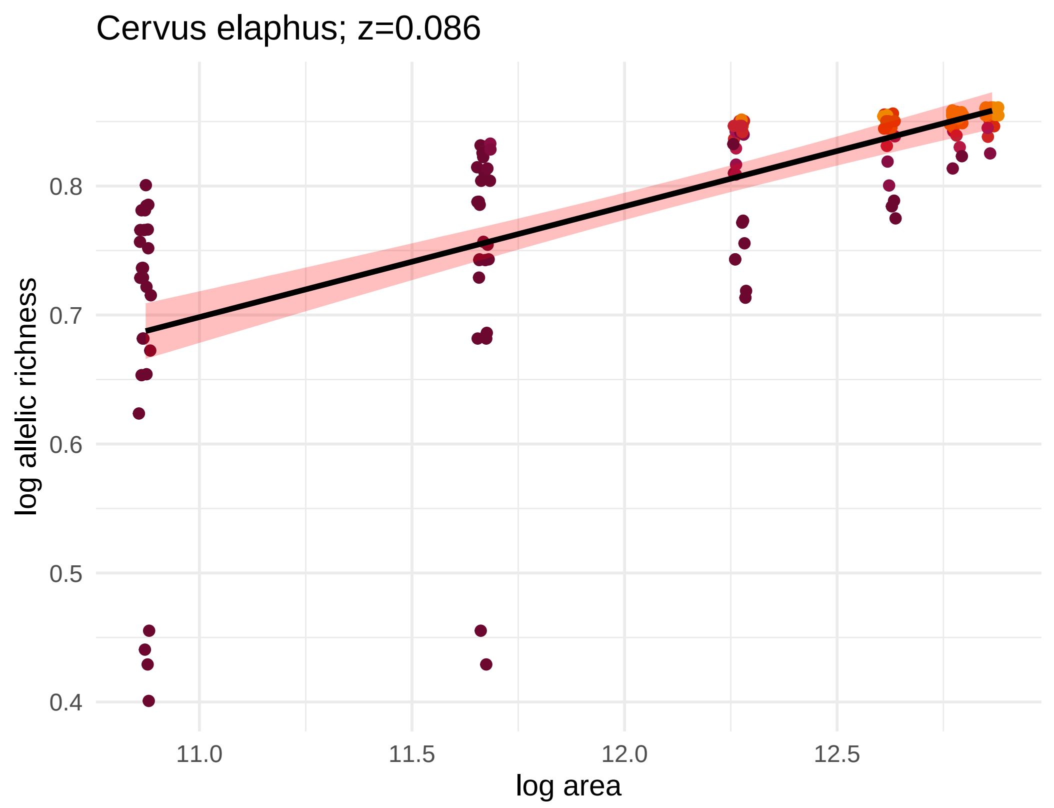
Cervus elaphus; z=0.086
log allelic richness
0.8
0.7
0.6
0.5
0.4
11.0
11.5
12.0
12.5
log area

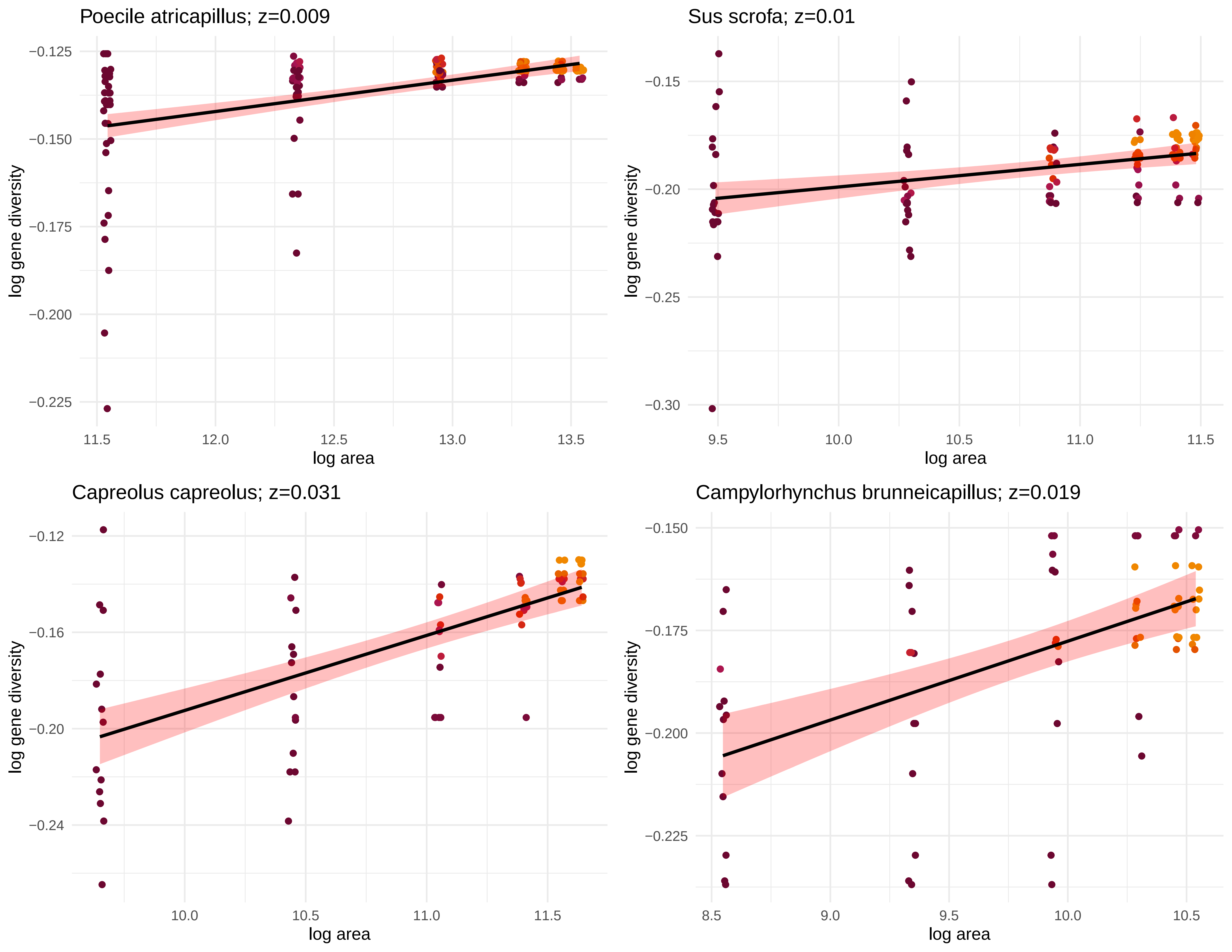
Poecile atricapillus; z=0.009
Sus scrofa; z=0.01
Capreolus capreolus; z=0.031
Campylorhynchus brunneicapillus; z=0.019
log gene diversity
log area
−0.125
−0.150
−0.175
−0.200
−0.225
11.5
12.0
12.5
13.0
13.5
−0.15
−0.20
−0.25
−0.30
9.5
10.0
10.5
11.0
11.5
−0.12
−0.16
−0.20
−0.24
10.0
10.5
11.0
11.5
−0.150
−0.175
−0.200
−0.225
8.5
9.0
9.5
10.0
10.5

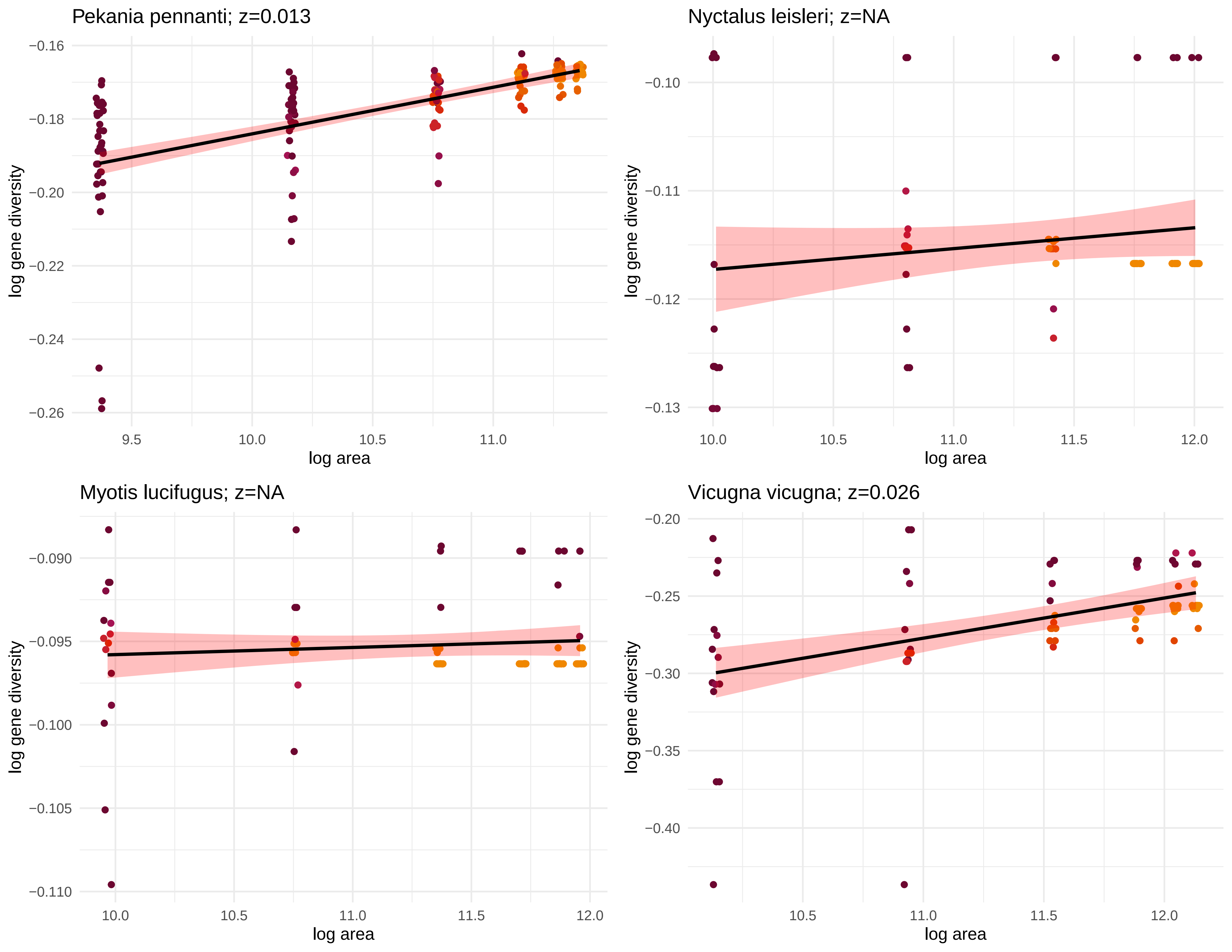
Pekania pennanti; z=0.013
log gene diversity
log area
−0.16
−0.18
−0.20
−0.22
−0.24
−0.26
9.5
10.0
10.5
11.0
Nyctalus leisleri; z=NA
log gene diversity
log area
−0.10
−0.11
−0.12
−0.13
10.0
10.5
11.0
11.5
12.0
Myotis lucifugus; z=NA
log gene diversity
log area
−0.090
−0.095
−0.100
−0.105
−0.110
10.0
10.5
11.0
11.5
12.0
Vicugna vicugna; z=0.026
log gene diversity
log area
−0.20
−0.25
−0.30
−0.35
−0.40
10.5
11.0
11.5
12.0

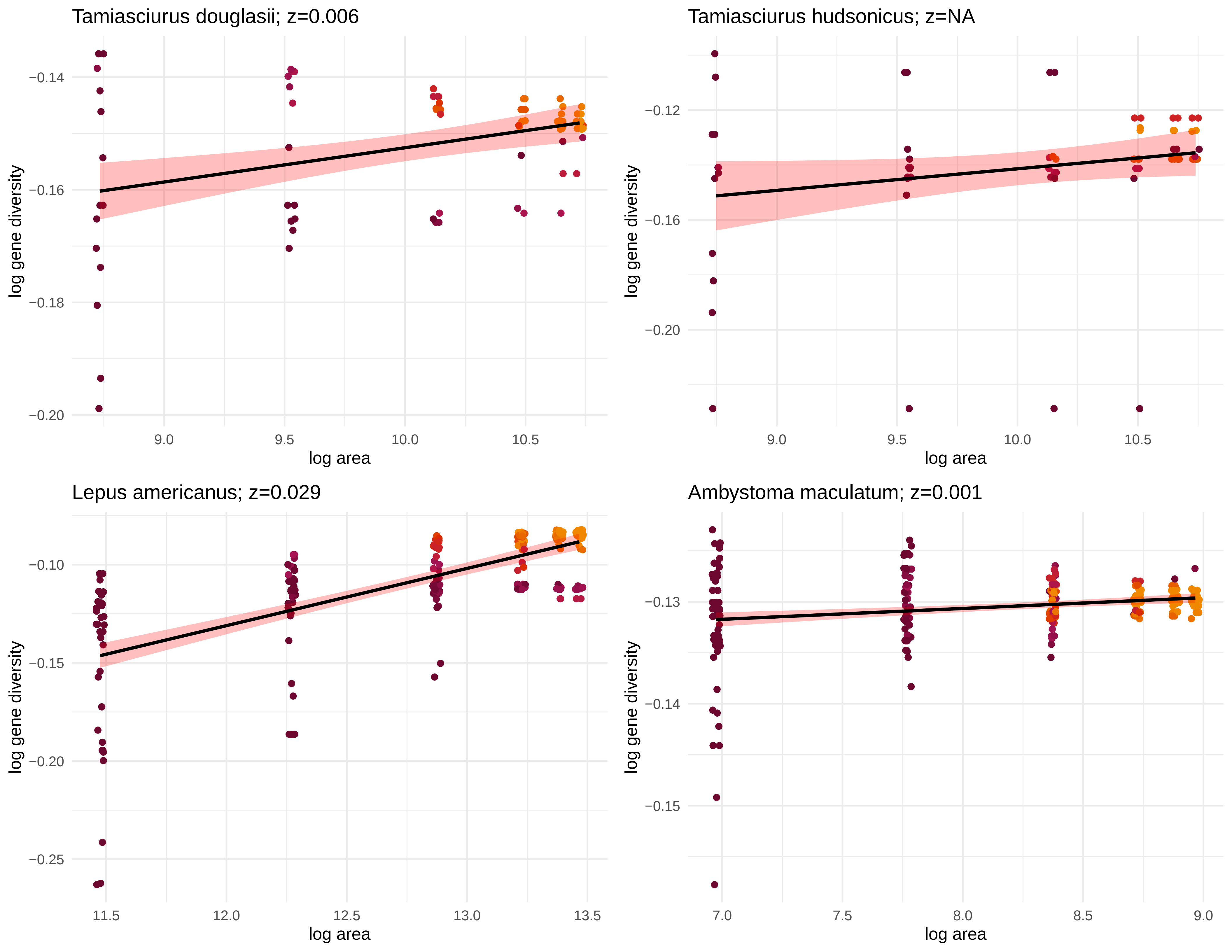

Tamiasciurus douglasii; z=0.006
Tamiasciurus hudsonicus; z=NA
Lepus americanus; z=0.029
Ambystoma maculatum; z=0.001
log gene diversity
log area
−0.14
−0.16
−0.18
−0.20
−0.12
−0.10
−0.15
−0.25
−0.13
9.0
9.5
10.0
10.5
11.5
12.0
12.5
13.0
13.5
7.0
7.5
8.0
8.5

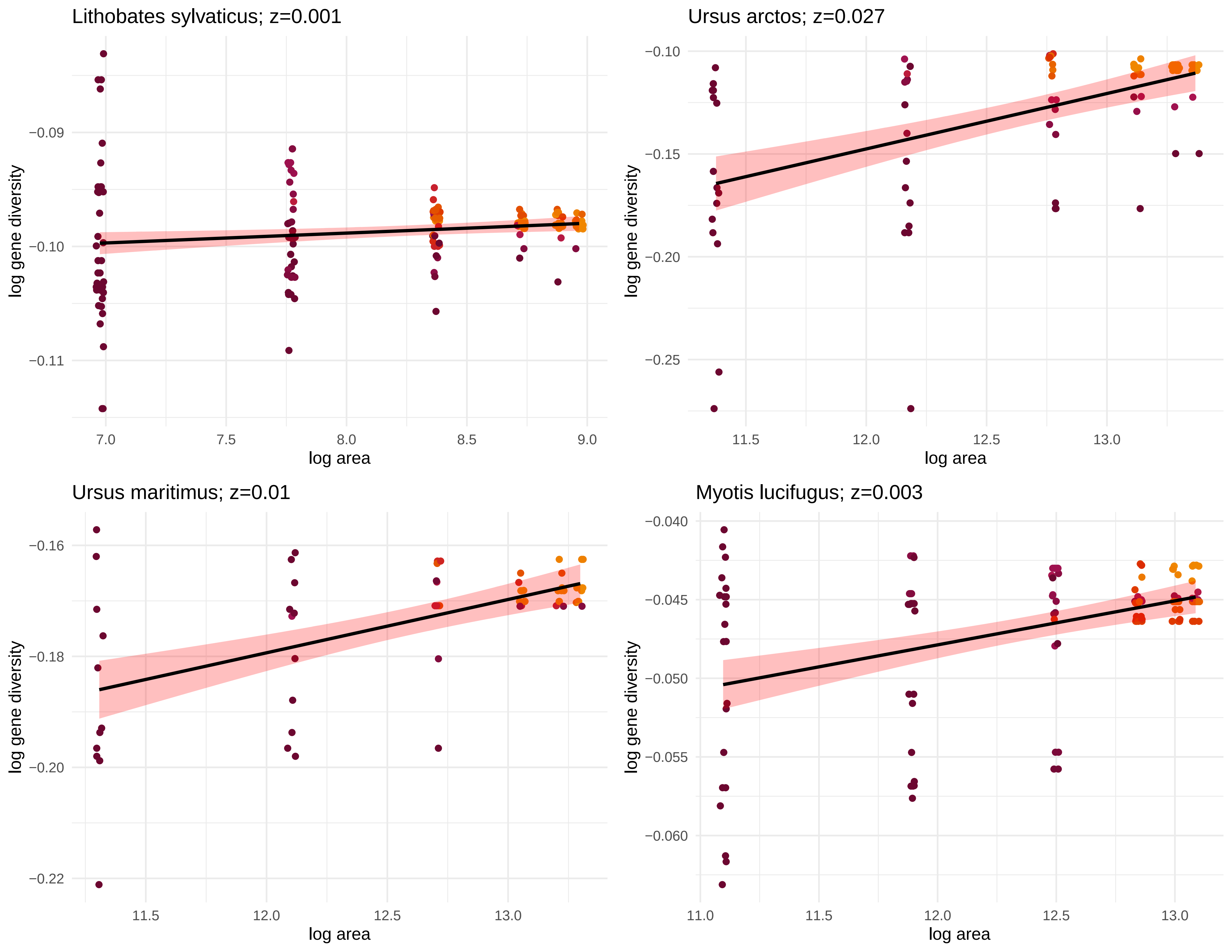

Lithobates sylvaticus; z=0.001
Ursus arctos; z=0.027
Ursus maritimus; z=0.01
Myotis lucifugus; z=0.003
log gene diversity
log area
−0.09
−0.10
−0.11
7.0
7.5
8.0
8.5
9.0
−0.10
−0.15
−0.20
−0.25
11.5
12.0
12.5
13.0
−0.16
−0.18
−0.20
−0.22
−0.040
−0.045
−0.050
−0.055
−0.060
11.0

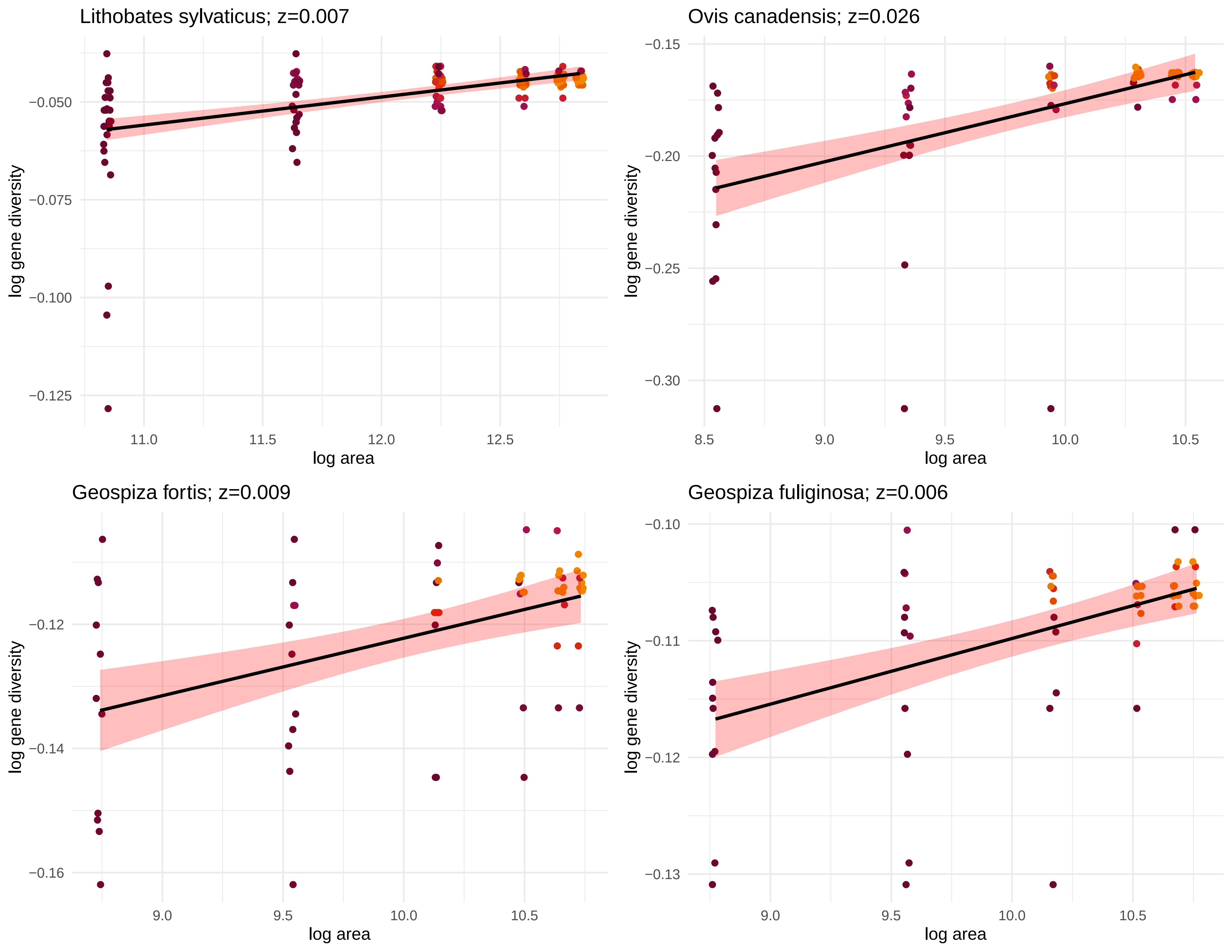
Lithobates sylvaticus; z=0.007
log gene diversity
−0.050
−0.075
−0.100
−0.125
11.0
11.5
12.0
12.5
log area
Ovis canadensis; z=0.026
log gene diversity
−0.15
−0.20
−0.25
−0.30
8.5
9.0
9.5
10.0
10.5
log area
Geospiza fortis; z=0.009
log gene diversity
−0.12
−0.14
−0.16
9.0
9.5
10.0
10.5
log area
Geospiza fuliginosa; z=0.006
log gene diversity
−0.10
−0.11
−0.12
−0.13
9.0
9.5
10.0
10.5
log area

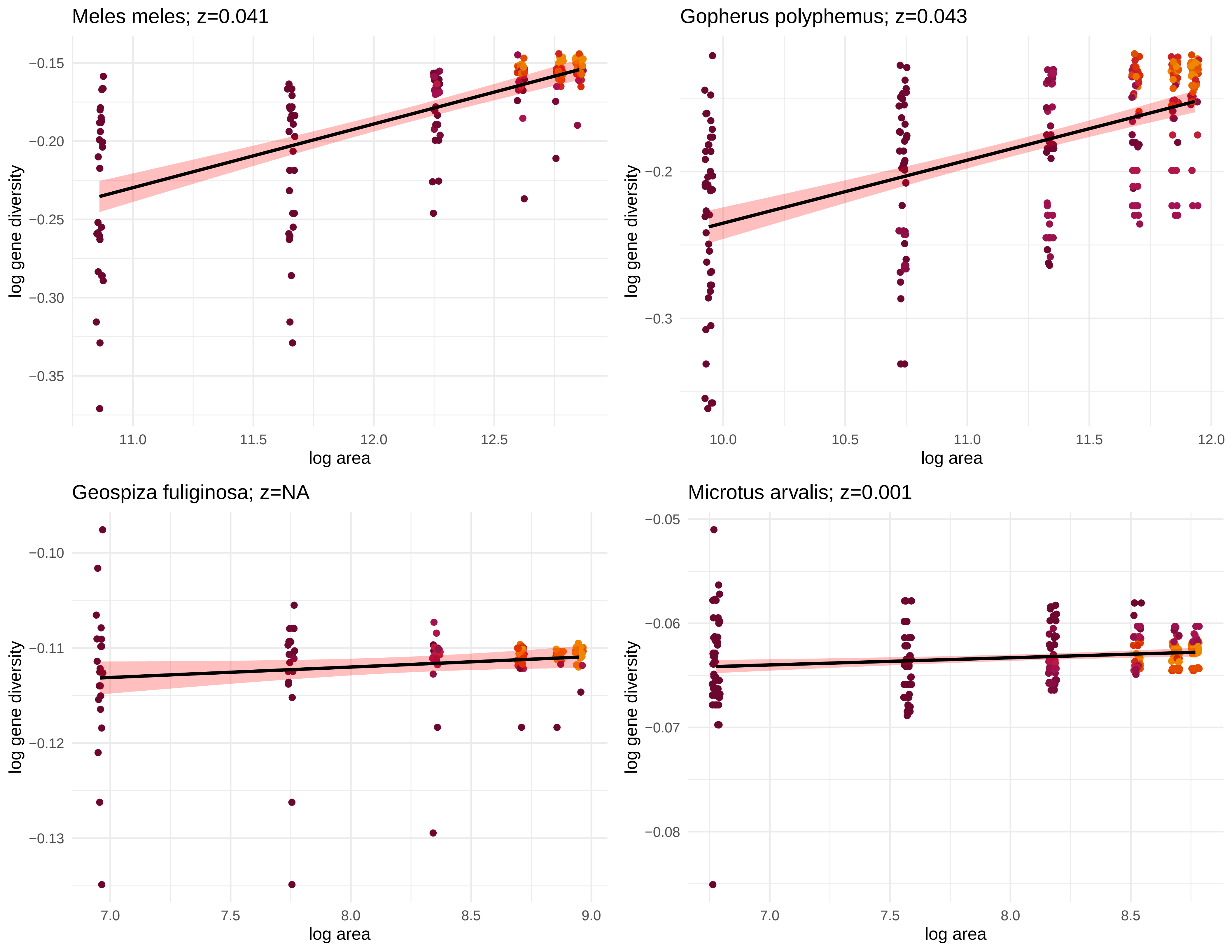

Meles meles; z=0.041
log gene diversity
−0.15
−0.20
−0.25
−0.30
−0.35
11.0
11.5
12.0
12.5
log area
Gopherus polyphemus; z=0.043
log gene diversity
−0.2
−0.3
10.0
10.5
11.0
11.5
12.0
log area
Geospiza fuliginosa; z=NA
log gene diversity
−0.10
−0.11
−0.12
−0.13
7.0
7.5
8.0
8.5
9.0
log area
Microtus arvalis; z=0.001
log gene diversity
−0.05
−0.06
−0.07
−0.08
7.0
7.5
8.0
8.5
log area

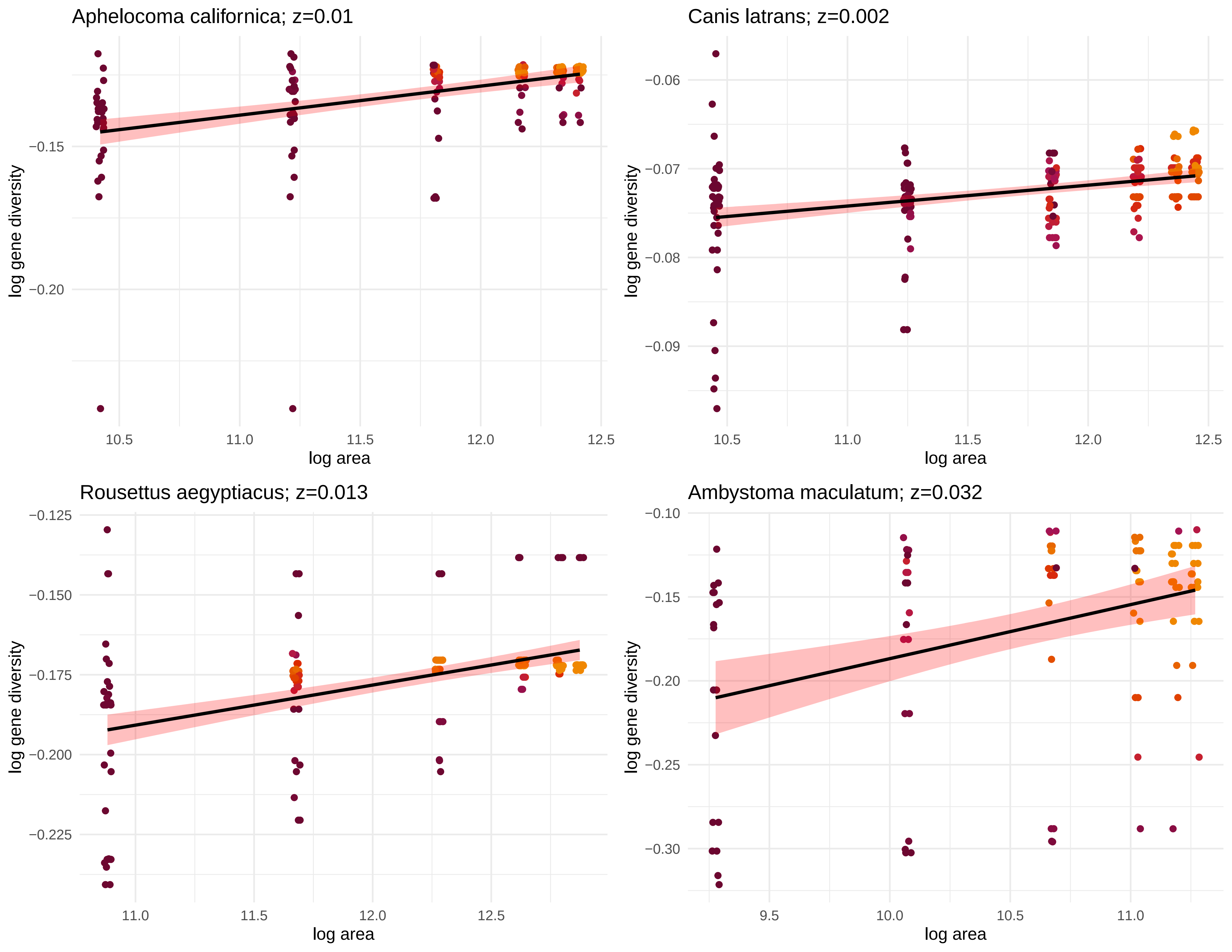

Aphelocoma californica; z=0.01
log gene diversity
−0.15
−0.20
10.5
11.0
11.5
12.0
12.5
log area
Canis latrans; z=0.002
log gene diversity
−0.06
−0.07
−0.08
−0.09
10.5
11.0
11.5
12.0
12.5
log area
Rousettus aegyptiacus; z=0.013
log gene diversity
−0.125
−0.150
−0.175
−0.200
−0.225
11.0
11.5
12.0
12.5
log area
Ambystoma maculatum; z=0.032
log gene diversity
−0.10
−0.15
−0.20
−0.25
−0.30
9.5
10.0
10.5
11.0
log area

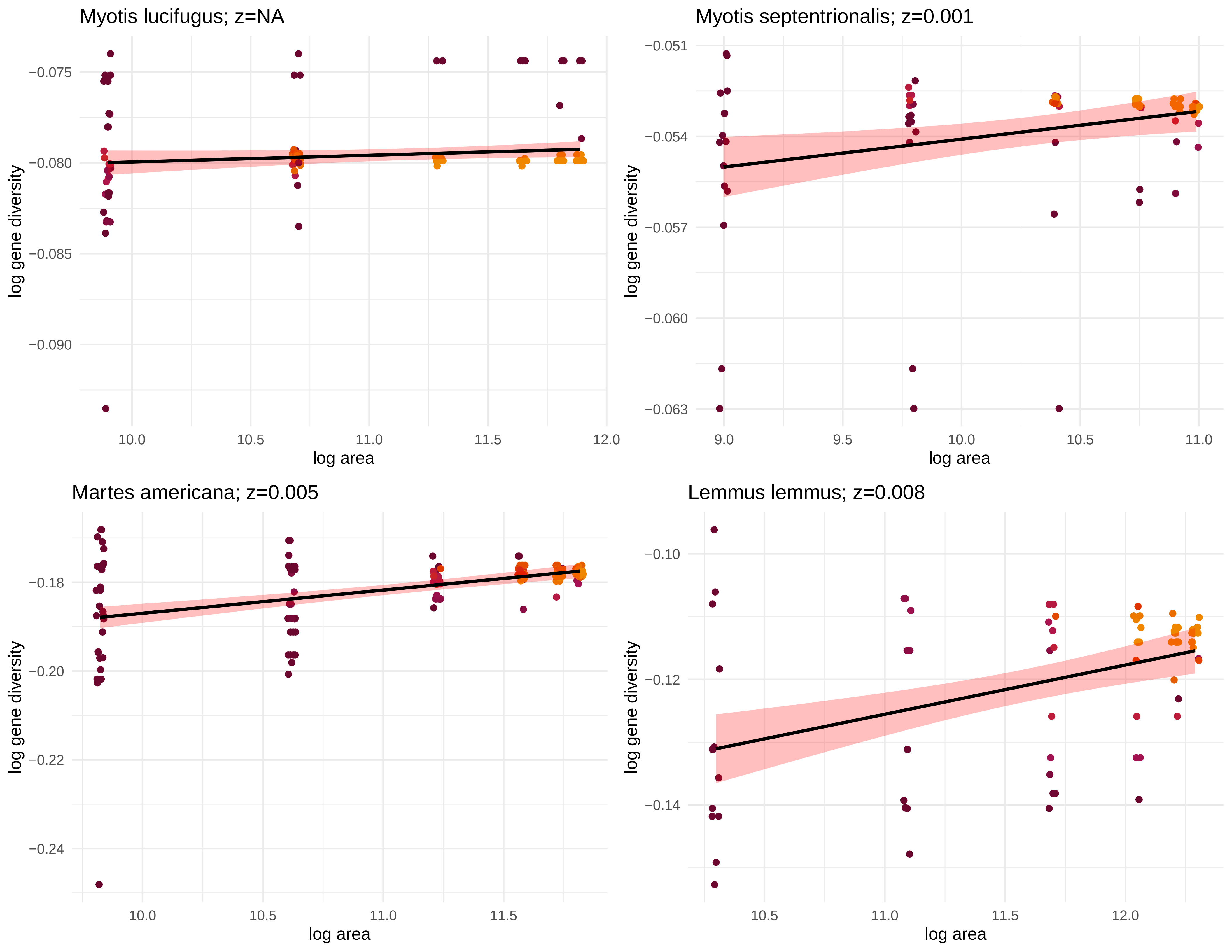
Myotis lucifugus; z=NA
Myotis septentrionalis; z=0.001
Martes americana; z=0.005
Lemmus lemmus; z=0.008
log gene diversity
log area
−0.075
−0.080
−0.085
−0.090
10.0
10.5
11.0
11.5
12.0
−0.051
−0.054
−0.057
−0.060
−0.063
9.0
9.5
10.0
10.5
11.0
−0.18
−0.20
−0.22
−0.24
10.0
10.5
11.0
11.5
−0.10
−0.12
−0.14
10.5
11.0
11.5
12.0

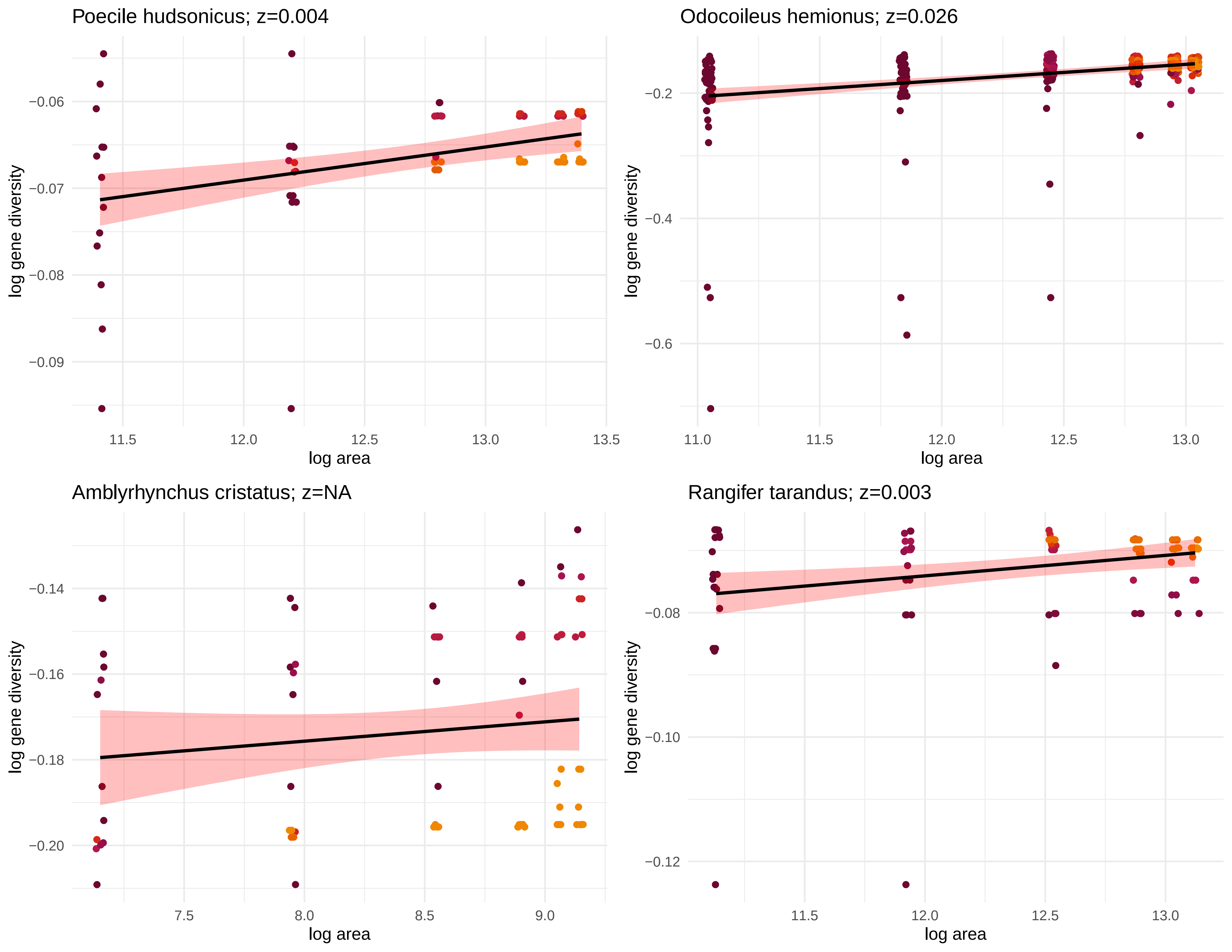

Poecile hudsonicus; z=0.004
Odocoileus hemionus; z=0.026
Amblyrhynchus cristatus; z=NA
Rangifer tarandus; z=0.003
log gene diversity
log area
−0.06
−0.07
−0.08
−0.09
11.5
12.0
12.5
13.0
13.5
−0.2
−0.4
−0.6
11.0
−0.14
−0.16
−0.18
−0.20
7.5
8.0
8.5
9.0
−0.08
−0.10
−0.12

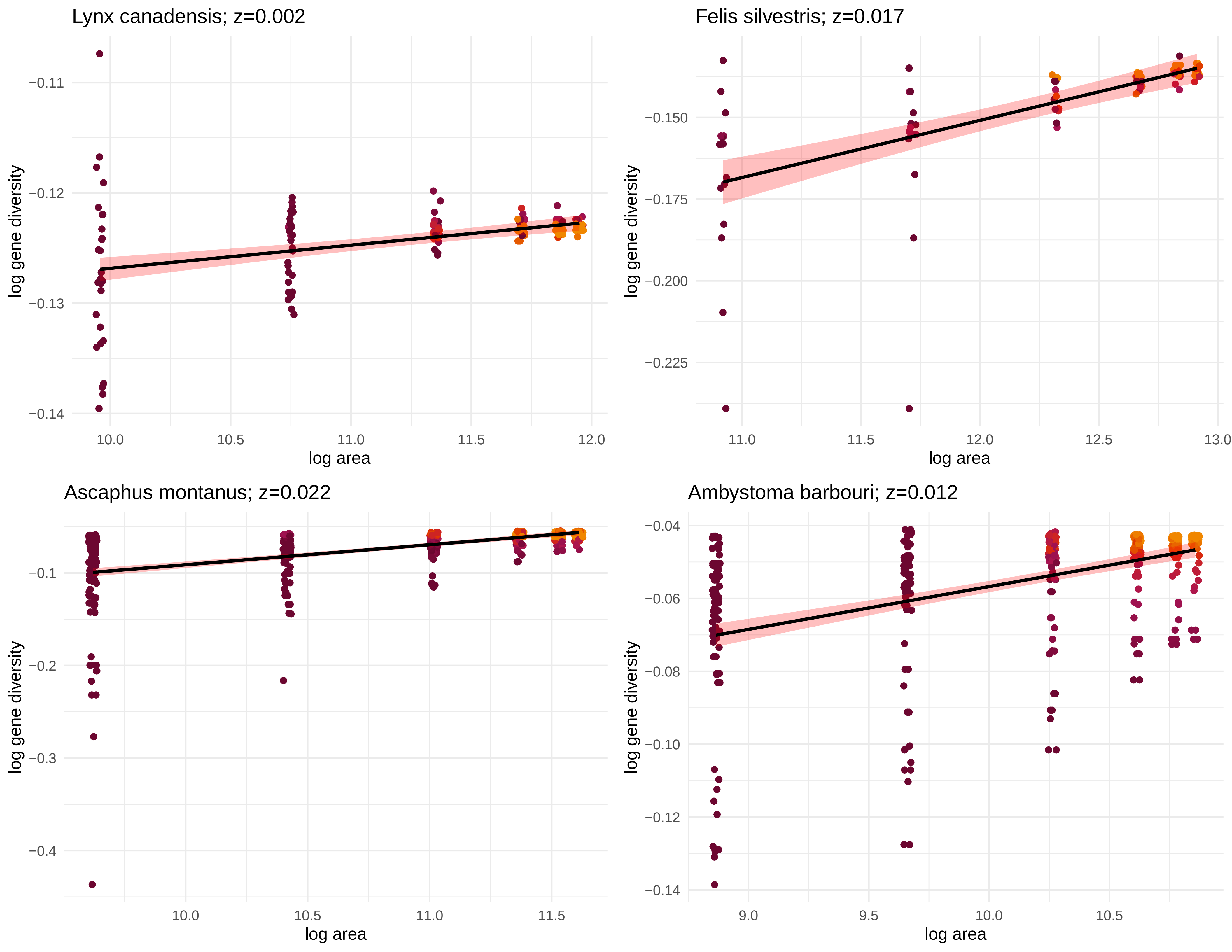
Lynx canadensis; z=0.002
log gene diversity
−0.11
−0.12
−0.13
−0.14
10.0
10.5
11.0
11.5
12.0
log area
Felis silvestris; z=0.017
log gene diversity
−0.150
−0.175
−0.200
−0.225
11.0
11.5
12.0
12.5
13.0
log area
Ascaphus montanus; z=0.022
log gene diversity
−0.1
−0.2
−0.3
−0.4
10.0
10.5
11.0
11.5
log area
Ambystoma barbouri; z=0.012
log gene diversity
−0.04
−0.06
−0.08
−0.10
−0.12
−0.14
9.0
9.5
10.0
10.5
log area

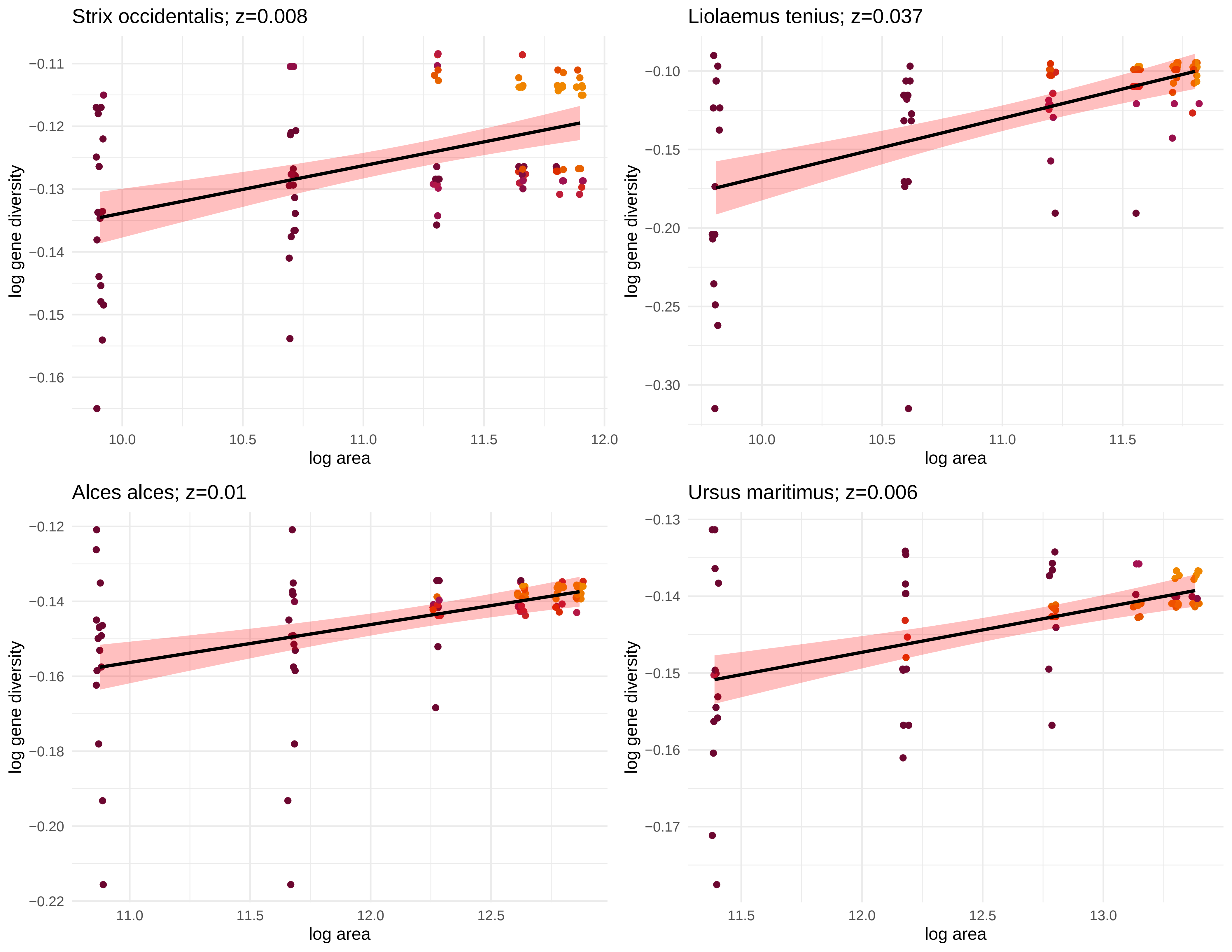
Strix occidentalis; z=0.008
Liolaemus tenius; z=0.037
Alces alces; z=0.01
Ursus maritimus; z=0.006
log gene diversity
log area
−0.11
−0.12
−0.13
−0.14
−0.15
−0.16
10.0
10.5
11.0
11.5
12.0
−0.10
−0.15
−0.20
−0.25
−0.30
−0.22
−0.18
−0.17
12.5
13.0

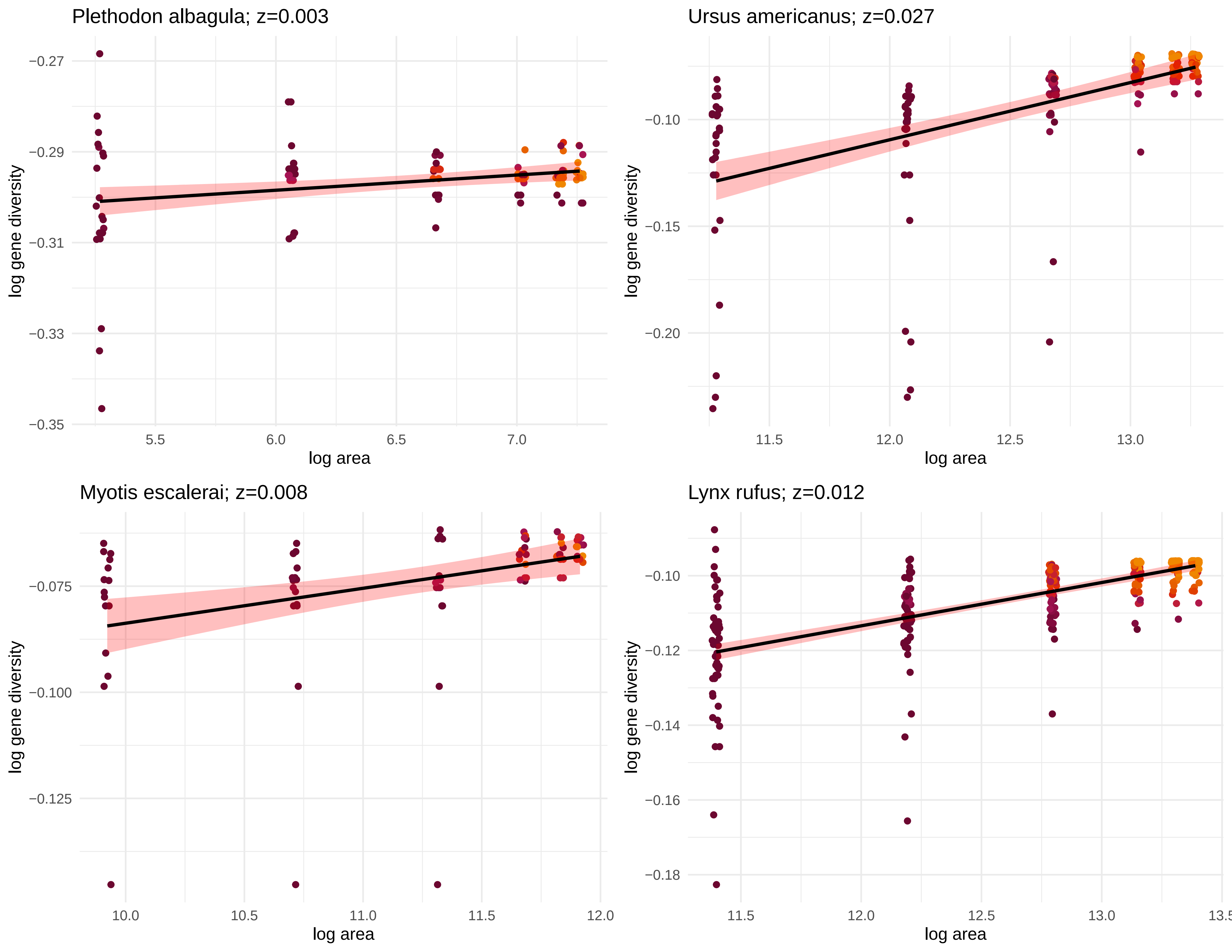
Plethodon albagula; z=0.003
log gene diversity
−0.27
−0.29
−0.31
−0.33
−0.35
5.5
6.0
6.5
7.0
log area
Ursus americanus; z=0.027
log gene diversity
−0.10
−0.15
−0.20
11.5
12.0
12.5
13.0
log area
Myotis escalerai; z=0.008
log gene diversity
−0.075
−0.100
−0.125
10.0
10.5
11.0
11.5
12.0
log area
Lynx rufus; z=0.012
log gene diversity
−0.10
−0.12
−0.14
−0.16
−0.18
11.5
12.0
12.5
13.0
log area

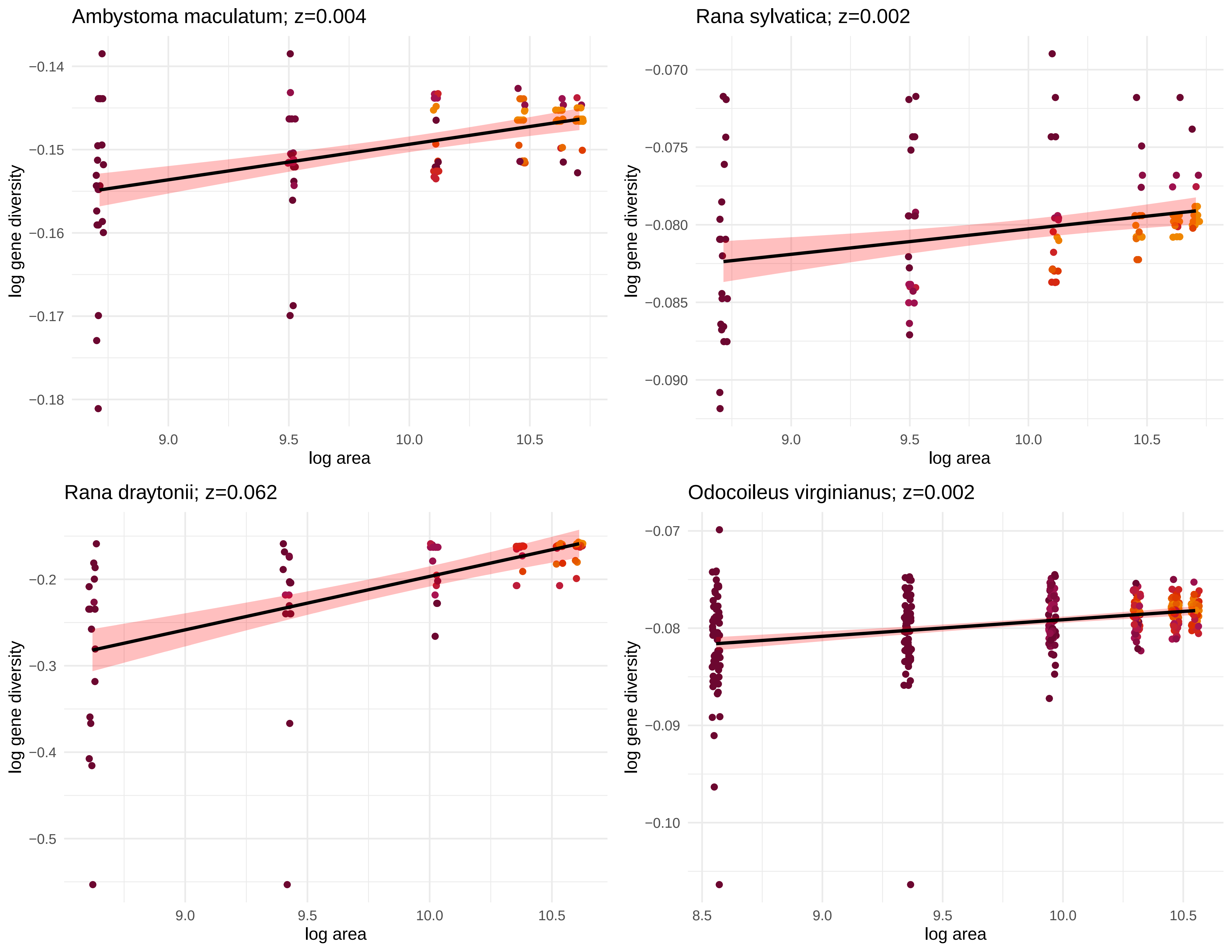

Ambystoma maculatum; z=0.004
Rana sylvatica; z=0.002
Rana draytonii; z=0.062
Odocoileus virginianus; z=0.002
log gene diversity
log area
−0.14
−0.15
−0.16
−0.17
−0.18
−0.070
−0.075
−0.080
−0.085
−0.090
−0.2
−0.3
−0.4
−0.5
−0.07
−0.08
−0.09
−0.10
8.5
9.0
9.5
10.0
10.5

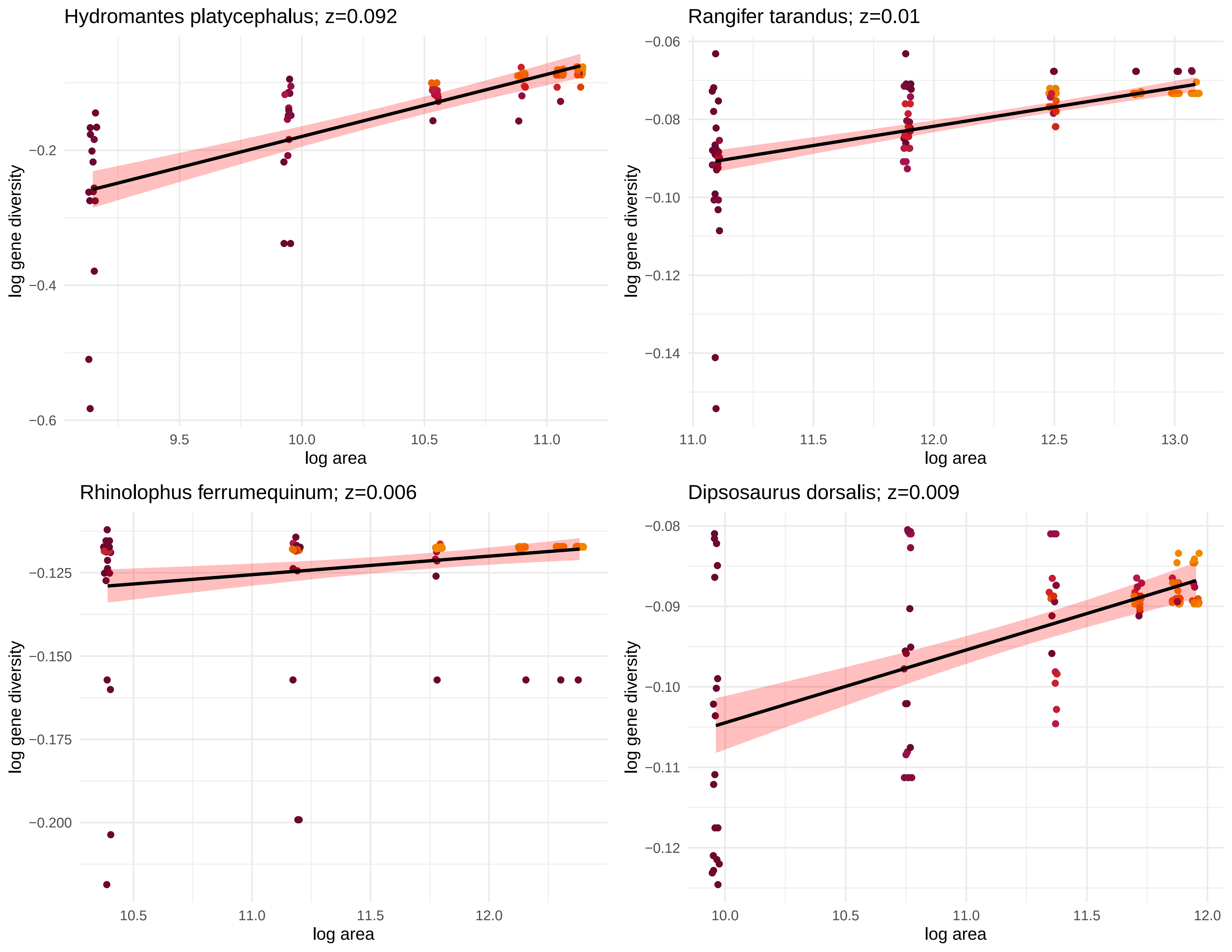
Hydromantes platycephalus; z=0.092
log gene diversity
log area
−0.2
−0.4
−0.6
9.5
10.0
10.5
11.0
Rangifer tarandus; z=0.01
−0.06
−0.08
−0.10
−0.12
−0.14
11.0
11.5
12.0
12.5
13.0
Rhinolophus ferrumequinum; z=0.006
−0.125
−0.150
−0.175
−0.200
10.5
11.0
11.5
12.0
Dipsosaurus dorsalis; z=0.009
−0.08
−0.09
−0.10
−0.11
−0.12
10.0
10.5
11.0
11.5
12.0

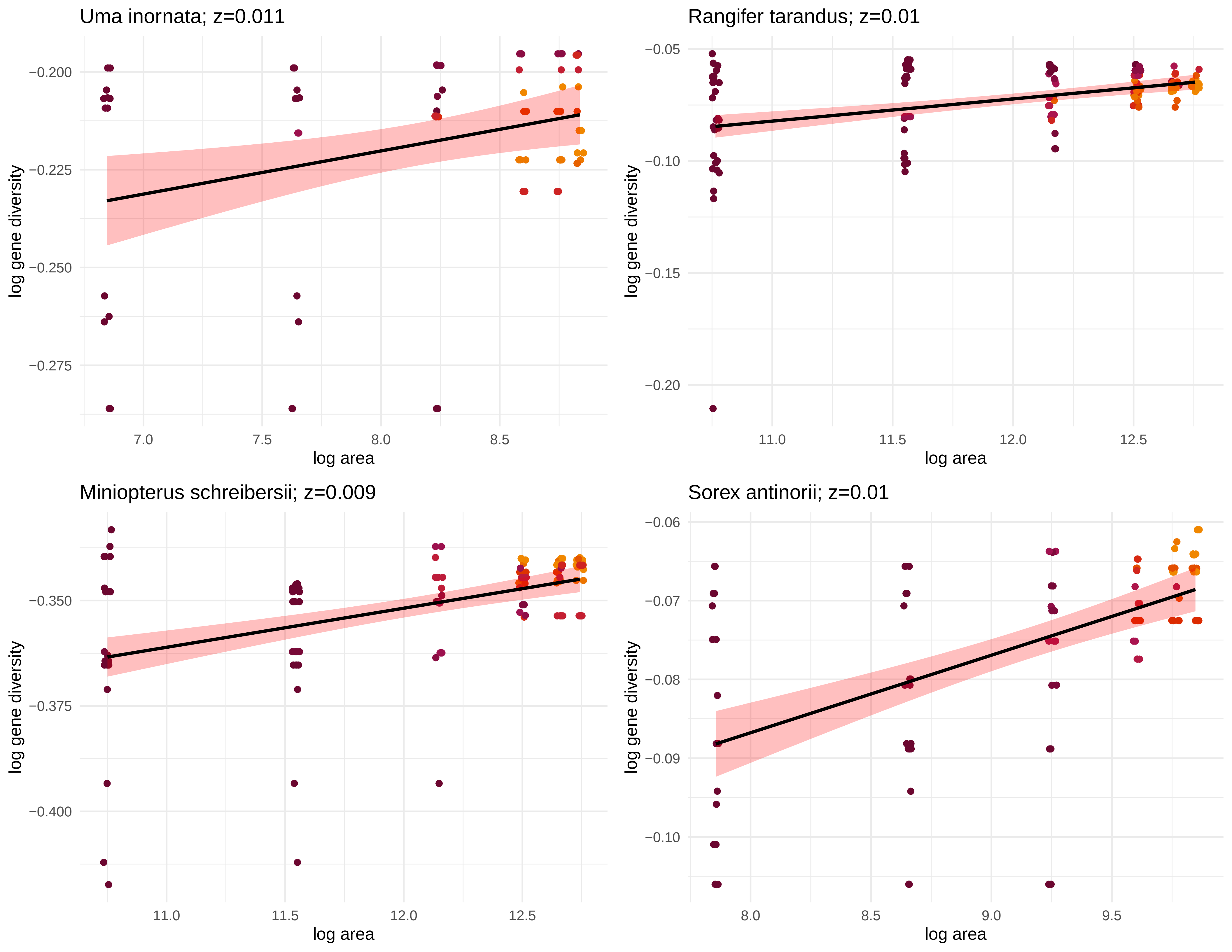
Uma inornata; z=0.011
Rangifer tarandus; z=0.01
Miniopterus schreibersii; z=0.009
Sorex antinorii; z=0.01
log gene diversity
log area
−0.200
−0.225
−0.250
−0.275
7.0
7.5
8.0
8.5
−0.05
−0.10
−0.15
−0.20
11.0
11.5
12.0
12.5
−0.350
−0.375
−0.400
−0.06
−0.07
−0.08
−0.09
−0.10
9.0
9.5

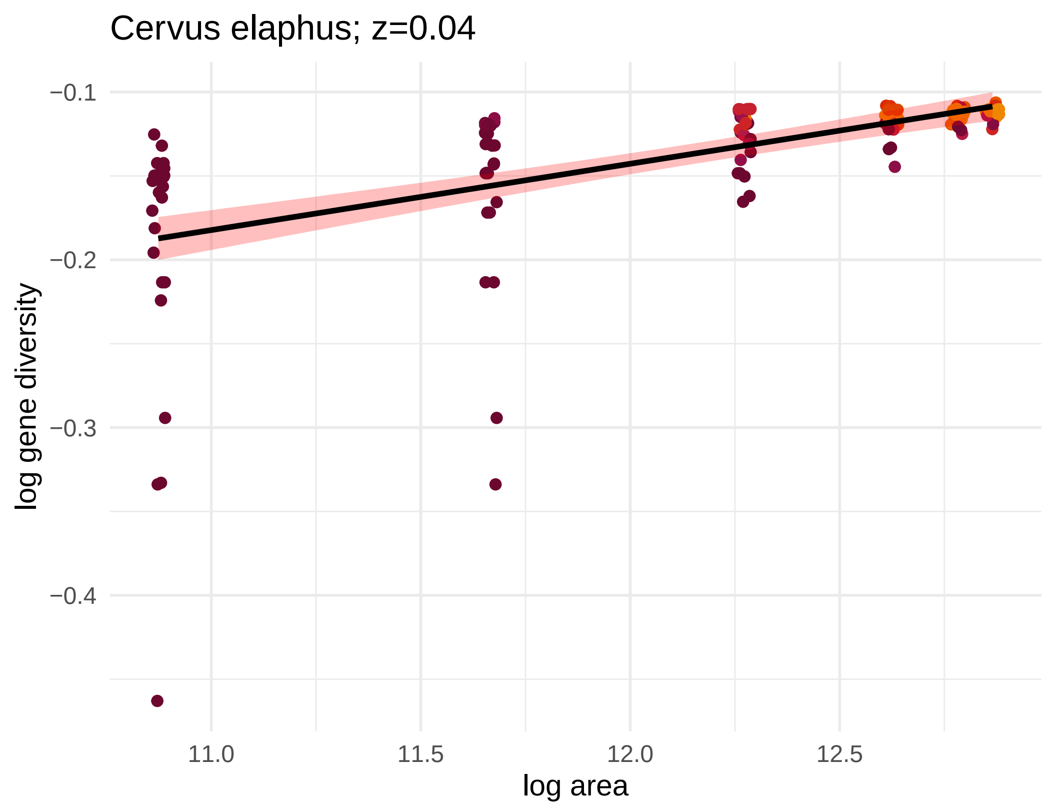

Cervus elaphus; z=0.04
−0.1
−0.2
−0.3
−0.4
log gene diversity
11.0
11.5
12.0
12.5
log area

Figure S3. Comparison of F_{ST} values from the data analyzed here versus mean F_{ST} estimates per species within the mammal MacroPopGen dataset for 12 species in common across both datasets. The red dashed line indicates a perfect correlation.

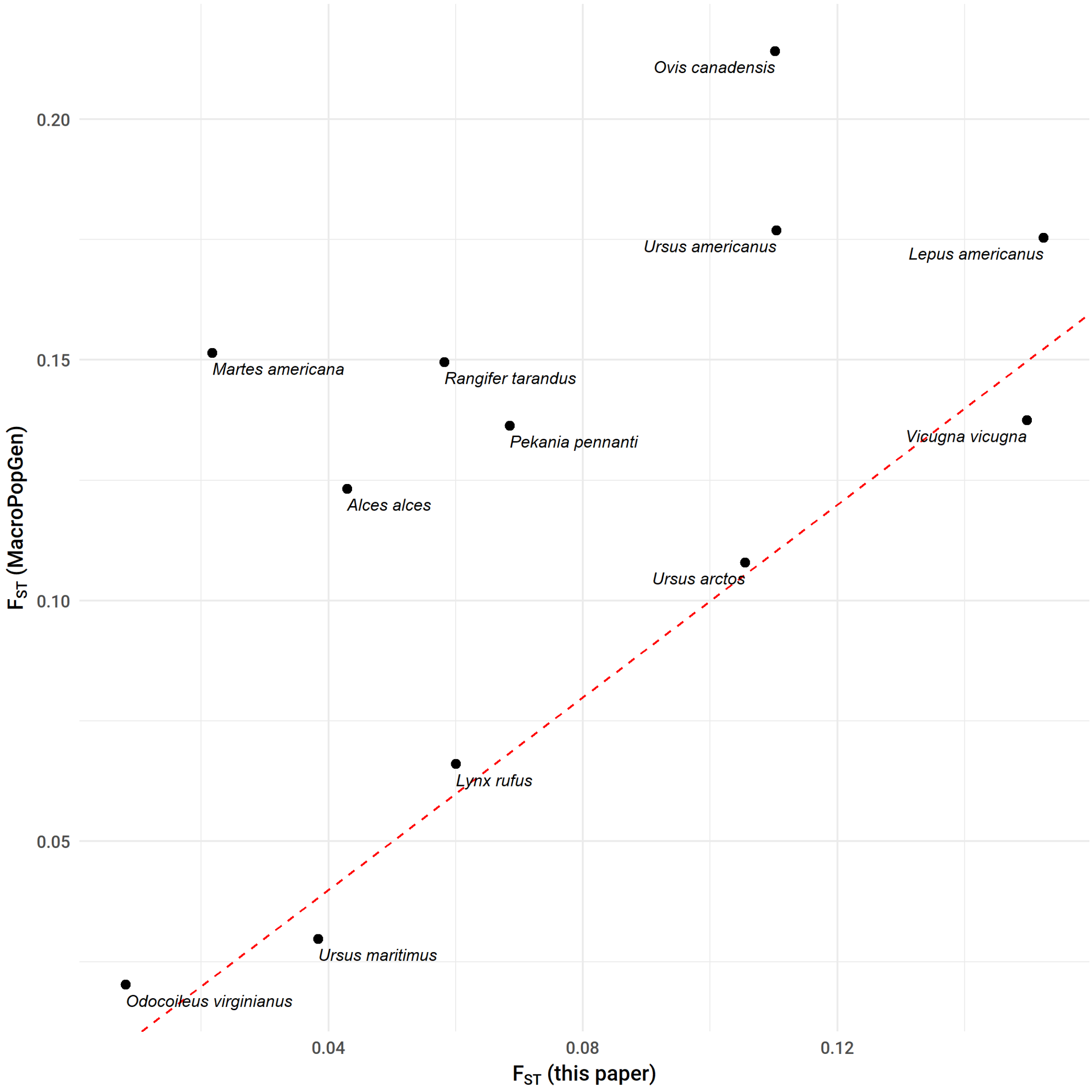

Figure S4. Observed MacroPopGen F_{ST} versus F_{ST} values predicted based on a model derived from the data analyzed here. Plots show predictions for individual datasets in the MacroPopGen database (left, points colored according to species; CV = 0.76) and predictions of the species mean F_{ST} averaged across datasets (right; CV = 0.69). The red dashed line indicates a perfect correlation.

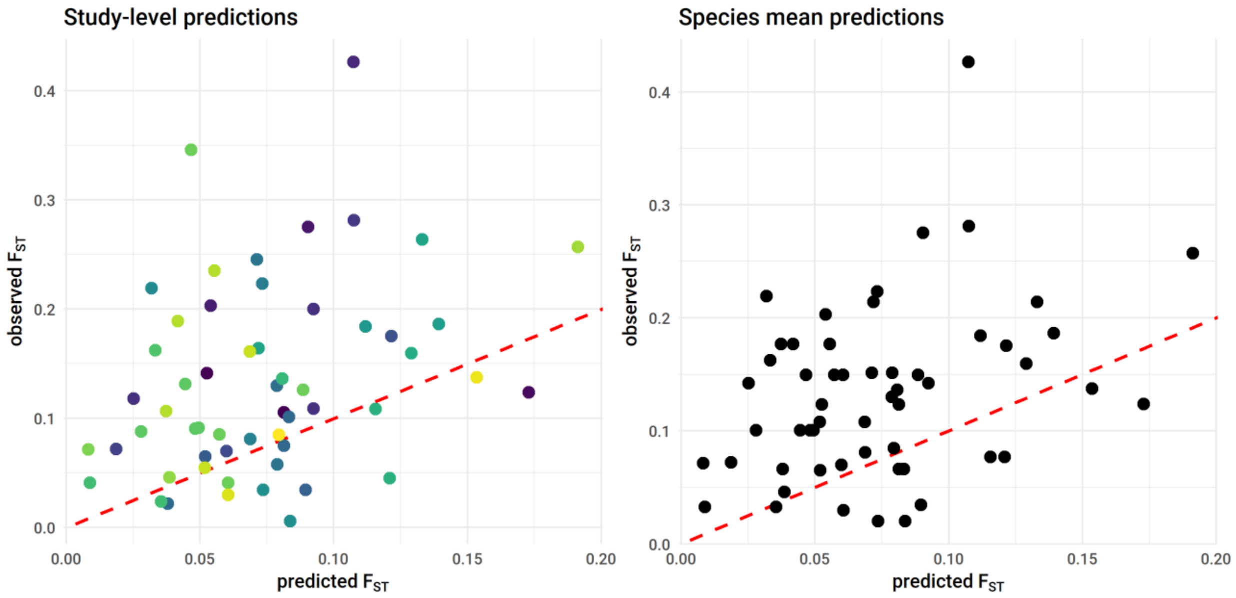

Figure S5. GDAR fit (R^2 of log-log relationships between area and each genetic metric) versus the number of loci and number of alleles per dataset. GDAR fit was unrelated to the number of loci and alleles sampled for most metrics except gene diversity, where GDAR fit was positively correlated with the number of loci. This positive correlation was dependent on the dataset with the largest number of loci.

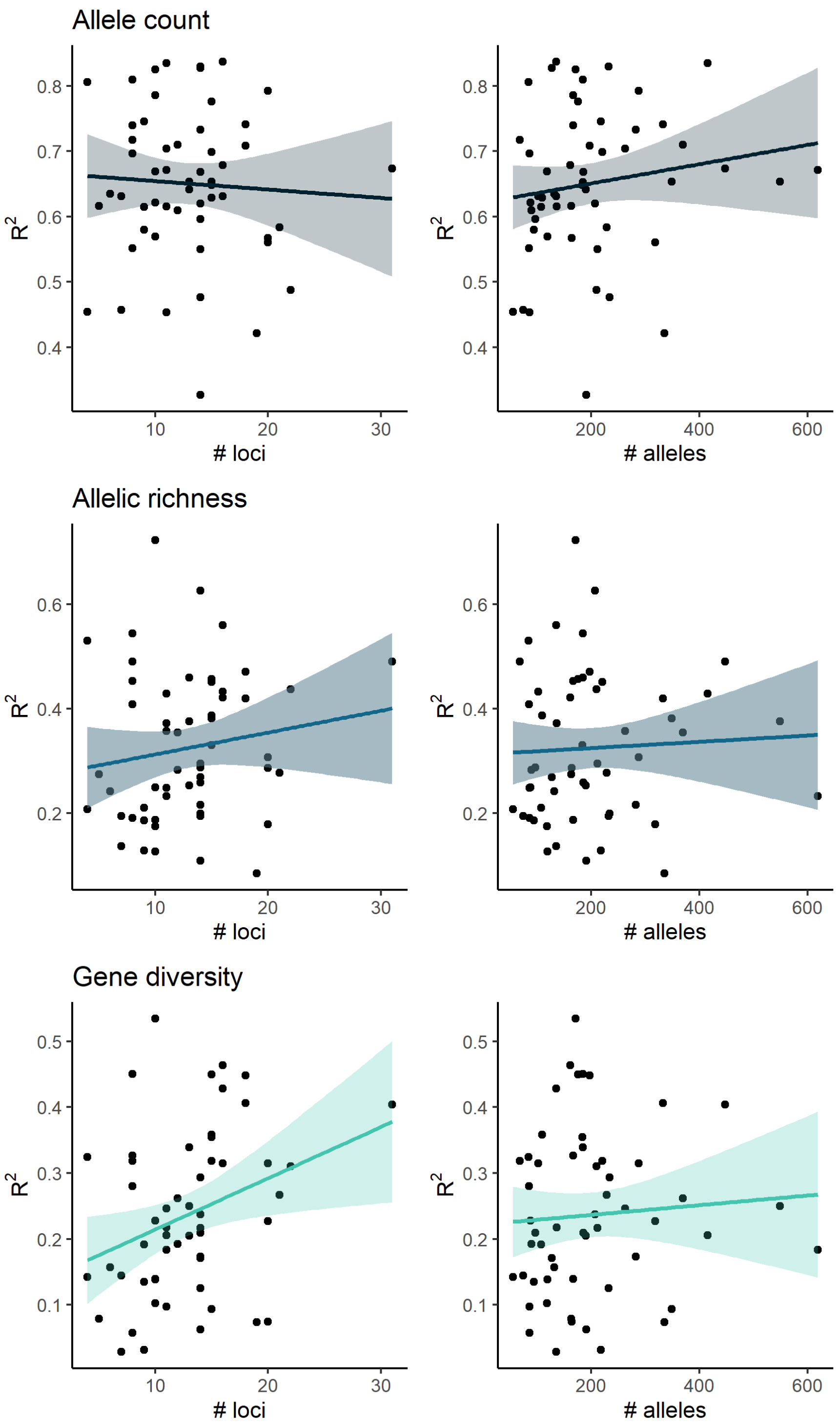

Supplement: Supplementary file 1 — Table S1: Data summary. The number of sites, total individuals across all sites, estimated global FST, estimated z values for each dataset (rows). Table S2:. GDAR scaling exponents (z‐values) for terrestrial vertebrates summarised across taxonomic groups (overall values) and for each taxonomic class. Table S3:. Model summaries testing whether F ST or z values differ from zero across taxa. Table S4: Comparison of area effect sizes between models with area alone, and global F ST and an area × F ST interaction as predictors. Table S5: Model summaries for relationships between F ST, z values derived from allele count (zAC), allelic richness (zAR), and gene diversity (zGD), and predictor variables including: home range size (km2), species range size (km2), species body mass (g), and the area of the spatial extent of the sample locations in each dataset (km2). Table S6: F ST estimates from the MacroPopGen database and associated trait data. Figure S1: Box plots of variance in genetic diversity explained by area (R 2) for each genetic metric. Figure S2: Genetic diversity versus area plotted on a log–log scale for all 61 datasets. Figure S3: Comparison of F ST values from the data analysed here versus mean FST estimates per species within the mammal MacroPopGen dataset for 12 species in common across both datasets. Figure S4: Observed MacroPopGen F ST versus FST values predicted based on a model derived from the data analyzed here. Figure S5: GDAR fit (R 2 of log–log relationships between area and each genetic metric) versus the number of loci and number of alleles per dataset. [file ELE-29-0-s001.pdf]
